# Analysis of proto-type Tarim Basin in the late Precambrian and the dynamic mechanism of its evolution

**DOI:** 10.1371/journal.pone.0286849

**Published:** 2023-06-07

**Authors:** Haining Chang, Guiting Hou, Shaoying Huang, Caiming Luo, Jinkai Xia, Ziqi Zhong, Xiang Li, Lunyan Wei

**Affiliations:** 1 The Key Laboratory of Orogenic Belts and Crustal Evolution, Ministry of Education, School of Earth and Space Sciences, Peking University, Beijing, China; 2 Institute of Petroleum Exploration and Development, Tarim Oilfield Company, Korla, China; Chinese Academy of Geological Sciences, CHINA

## Abstract

Tarim Basin has undergone an intricate tectonic evolution history ever since its formation from two discrete terranes in Neoproterozoic rather than in the Paleoproterozoic. More precisely, the amalgamation is assumed to happen during 1.0–0.8 Ga based on plate affinity. As the beginning of a unified Tarim block, studies of Tarim Basin in the Precambrian are basic and important. After the amalgamation of south and north paleo-Tarim terranes, Tarim block was experiencing a complicated tectonic process of being affected by mantle plume related to the breakup of Rodinia supercontinent in the south, and compressed by the Circum-Rodinia Subduction System in the north. The breakup of Rodinia supercontinent finished in the late Sinian Period, leading Kudi Ocean and Altyn Ocean to open and separating Tarim block from itself. According to the residual strata thickness, drilling data, and lithofacies distribution, the proto-type basin and tectono-paleogeographic maps of Tarim Basin in the late Nanhua Period and Sinian Period are reconstructed. With these maps, the characteristics of the rifts are revealed. Two rift systems were developed inside the unified Tarim Basin in the Nanhua Period and Sinian Period, one back-arc rift system in the northern margin and the other aulacogen system in the southern margin. The azimuth distribution of the rifts in Quruqtagh showed a predominant NE-SW trend, and the rifts in Aksu trended mainly NW-SE, while the rifts in Tiekelike trended SW-NE. With a three-dimensional elastic FEM (Finite Element Method) model that includes all rifts and deposited areas in Tarim Basin, applying the southern subduction and northern mantle upwelling properly to get the paleotectonic mian stress axes and the differential stress field, the dynamic mechanisms of rifts evolution are proved to be related to the peripheral tectonic environment mentioned above.

## 1. Introduction

**Fig 1:**
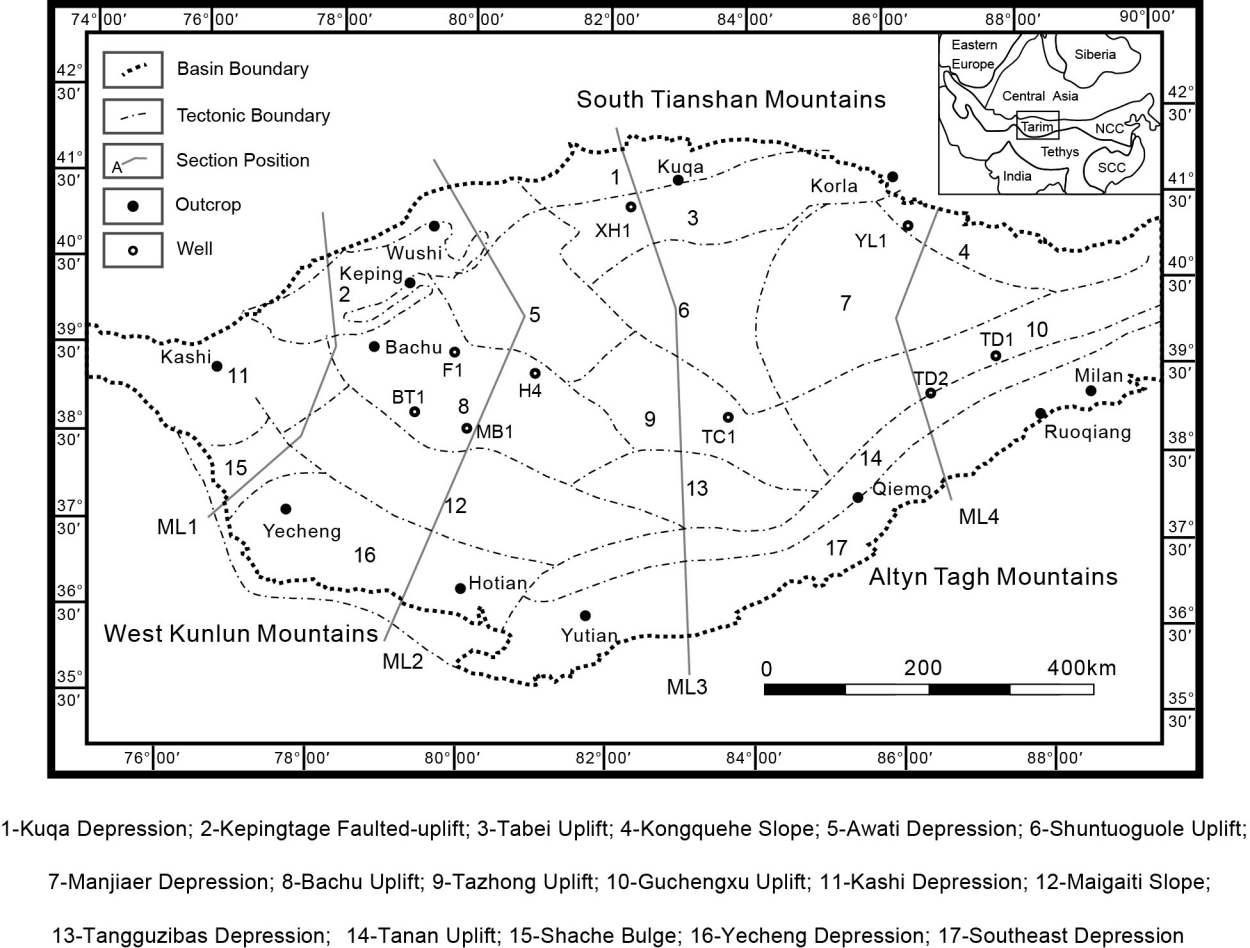
Tectonic framework of Tarim Basin (modified after [9, 10]). ML1, ML2, ML3, ML4 are measuring lines used to describe the extension of shortening of Tarim Basin. Republished from Zhong et al. (2023) under a CC BY license, with permission from *Frontiers in Earth Science*, original copyright 2023.

As the largest intraplate basin in China, Tarim Basin covers an area of over 560,000 km^2^, containing abundant oil and gas resources. In order to guide the exploration and exploitation of oil and gas in Tarim Basin, it’s important to analyze the evolution of proto-type Tarim Basin and its surrounding plate tectonic configuration in the late Precambrian. Knowing how Tarim Basin acted in the Precambrian Period and what motivated it could offer a detailed image of the early stage of its evolution and tectono-paleogeography. Besides, late Neoproterozoic era is the beginning of the entire history of a unified Tarim Basin, thus a thorough investigation of the Precambrian evolution and tectono-paleogeography of proto-type Tarim Basin could provide a solid foundation for the entire research. However, most of the craton is covered by Mesozoic and Cenozoic sediments. Precambrian outcrops are only exhumed on the margin of Tarim Basin in Quruqtagh (northeast of Tarim), Aksu, West Kunlun and Altyn Tagh mountain belts (Fig 1) [1–4]. Due to the difficulties in accessibility, the Precambrian research of Tarim Basin has not reached a consensus. For example, the proto-type basin and tectono-paleogeography of Tarim Basin have not been solved perfectly. Many studies have only focused on Tarim Basin itself or even a part of it [5, 6], neglecting its adjacent areas, which is not enough to analyze the dynamic factors that contributed to the evolution of Tarim Basin, or to provide an overview of the whole circumstances. Apart from these, some studies only provided analysis of the proto-type basin or rifting development in the late Neoproterozoic, but never came up with a thorough presentation of its distribution [7, 8].

From the residual stratum thickness of Tarim Basin in the Nanhua Period and Sinian Period (Bureau of Geophysical Prospecting INC.), combining the lithofacies from wells and outcrops [11], a detailed map of the proto-type Tarim Basin in the Nanhua Period and Sinian Period are figured out. Based on the proto-type Tarim Basin distribution and the investigation of its adjacent areas, an overview of tectonic paleogeographic framework of Tarim Basin and its adjacent areas is proposed. These tectono-paleogeographic figures could provide an exhaustive image of Precambrian Tarim Basin and its adjacent areas and narrate the evolution of Tarim Basin. Based on the geological settings, a three-dimensional elastic Finite Element Method (FEM) model that includes all rifts and deposited areas in Tarim Basin is constructed to present the dynamic mechanisms of rifts evolution in this paper as well.

## 2. Geological setting and plate tectonic configuration

### 2.1 Regional setting

Located in the northwestern China, Tarim Basin is quite complicated as one of the oldest continental blocks in China (Fig 1). It is surrounded by orogenic belts, which are the South Tianshan Mountains to the north, the West Kunlun Mountains to the south, and the Altyn Tagh Mountains to the southeast [12–16].

Tarim Basin was formed from the amalgamation of the southern Tarim terrane and northern Tarim terrane during 1.0–0.8 Ga [13, 17, 18]. Around the same time, Rodinia supercontinent was experiencing a breakup [19–21], which lead to rifts in both southern and northern Tarim block [17, 22–24]. The basement of Tarim Basin is composed of Archean and Proterozoic igneous and metamorphic rocks, interpreted as a fragment of the Rodinia supercontinent [25, 26]. Consequently, the late Neoproterozoic tectonic movements could significantly affect the basin formation and material transportation of Tarim Basin in its early stage, which could play a pivotal role in the possible distribution of resources in Precambrian and Cambrian Period [5, 12, 20].

### 2.2 Tarim block in the global plate tectonics and its evolution

Tarim block had experienced several stages of tectonic evolution in the Precambrian, with both similarities and dissimilarities to the south and north Tarim blocks in many aspects [2, 17, 18, 22, 24, 27]. Figuring out the plate tectonic history would help to understand how Tarim Basin came into being and how the subbasins inside Tarim Basin have evolved [20, 28, 29].

There are two things worth paying attention to: the amalgamation of south and north paleo-Tarim terranes, the relationship between Tarim and Rodinia supercontinent.

#### (1) The amalgamation of south and north paleo-Tarim terranes

The amalgamation of south and north paleo-Tarim terranes is one of the most controversial problems during the evolution of Tarim Basin [2, 24]. To make this process clear would offer a solid fundamental for the throughout stage research of Tarim Basin.

**Fig 2:**
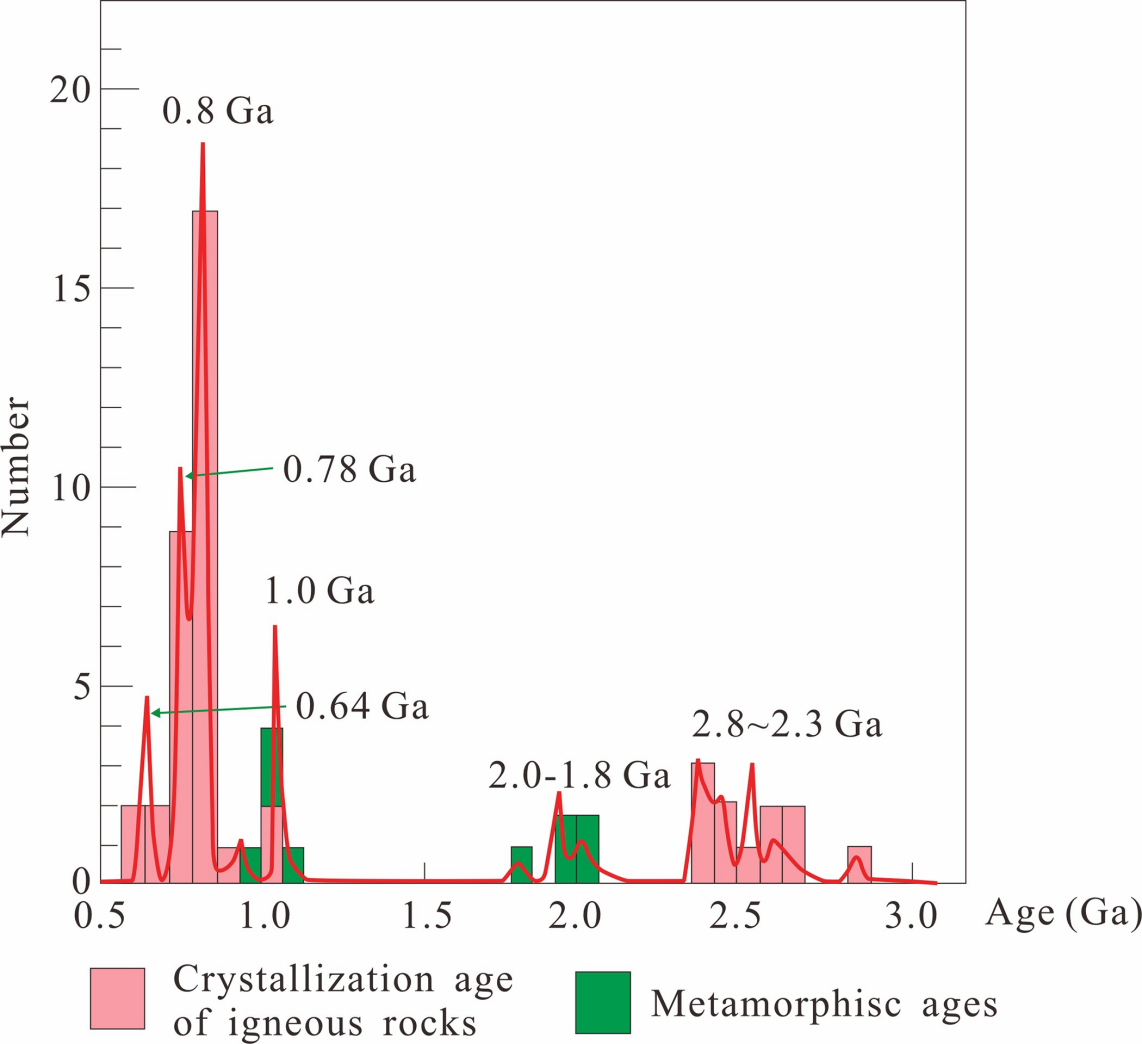
Frequency of thermo-tectonic events in Tarim since 2.6 Ga (modified after [31]). (Quruqtagh and Keping-Asku are located in the Northern Tarim, while Altyn and West Kunlun are in the Southern Tarim).

According to the frequency of thermo-tectonic events (Fig 2), it’s not synchronous between south and north paleo-Tarim terranes until 1.0 Ga. Between 2.6 Ga and 1.6 Ga, there were no thermo-tectonic events in Keping-Aksu, few in Quruqtagh, but quite a few in Southern Tarim (Altyn and West Kunlun) [30]. During 1.6–1.0 Ga, thermo-tectonic events happened a lot in South Tarim, while barely in Northern Tarim (Quruqtagh and Keping-Aksu). However, after 1.0 Ga, thermo-tectonic events were happening frequently both in south and north Tarim, which implies these two parts had collided as a whole since 1.0 Ga [2, 17, 18, 24, 31].

**Fig 3:**
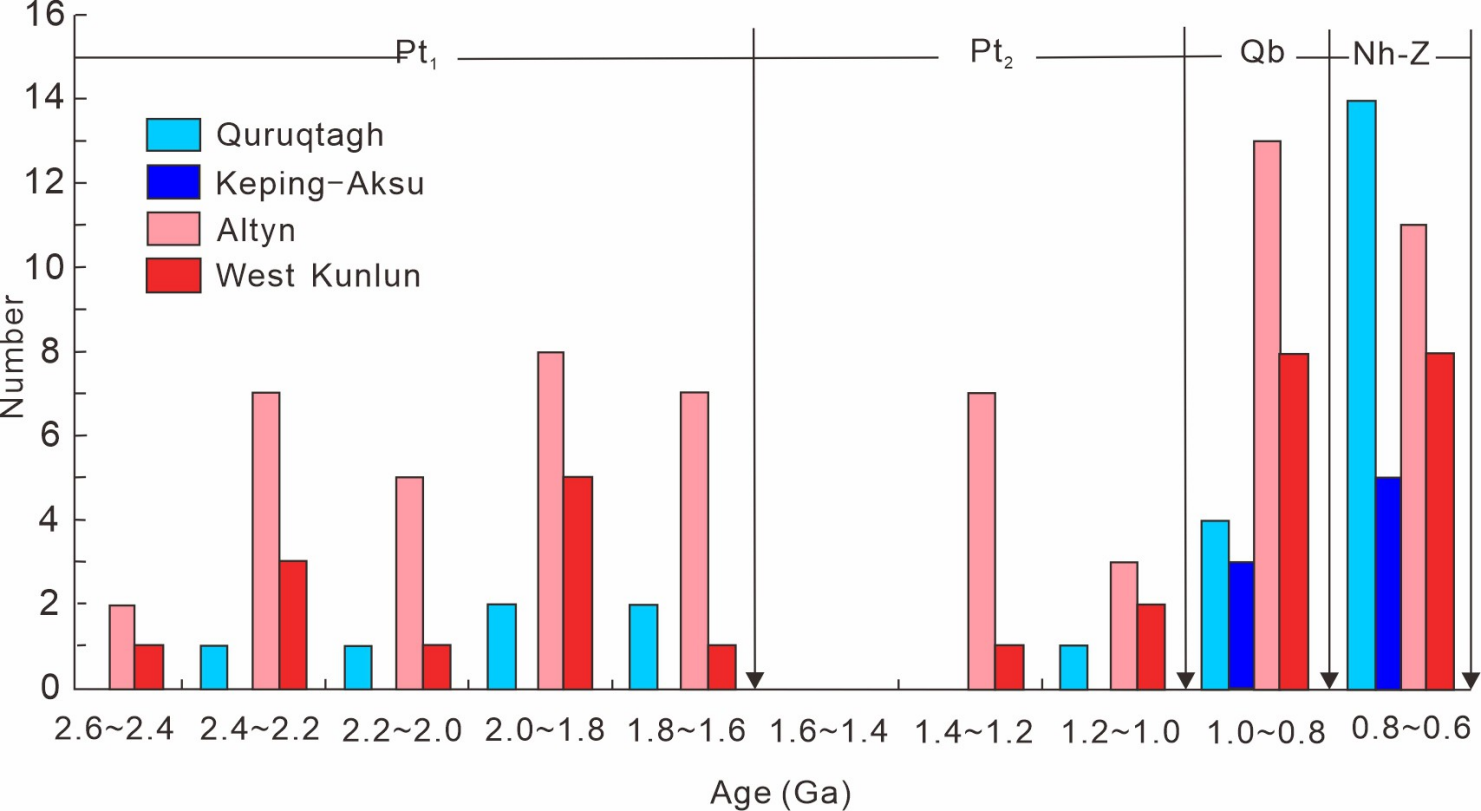
Magmatism and metamorphism events in Tarim since 3.0 Ga, the red line describes the trend of the events (modified after [34, 35]).

During 2.0–1.8 Ga, the first regional metamorphism happened in Tarim (Fig 3). Due to the aforementioned analysis, it was interpreted as caused by the subduction between south and north paleo-Tarim terranes but not combined yet. Instead, the last regional metamorphism (ca. 0.9 Ga) marked the collision and amalgamation of south and north paleo-Tarim terranes. This event coincided with the global Grenvillian orogenic movement, which collapsed Yangtze and Cathaysia together to form the South China Craton (SCC) [25, 32, 33].

New evidence showed that the blueschist in Aksu group in the northeast of Tarim was formed by plate subduction during 0.73–0.8 Ga [1, 3, 22, 36, 37]. As a result, it was not related to the subduction between south and north paleo-Tarim terranes, but formed along the southward subduction between the northern margin of the already combined Tarim and the South Tianshan Ocean crust due to the assembly of Rodinia [38, 39]. Thus, the deduction that the suture zone of south and north paleo-Tarim terranes in 1.0–0.8 Ga turned to the northwest was problematic [40].

#### (2) The relationship between Tarim and Rodinia supercontinent based on plate affinity

Previous studies on the relationship between Tarim block and Rodinia supercontinent have not reached a consensus. It is inappropriate to neglect the affinity between North China Craton (NCC) and Siberia, and put the paleolatitude data of Tarim in a minor position [4, 41]. Besides, due to the lack of high-confidence paleomagnetic data, the NCC was assumed to be fixed, while the bigger Laurentia and Siberia were drifty, which is unreasonable. Also, placing North China far from Siberia, which two shared a strong plate affinity, is improper as well [20].

**Fig 4:**
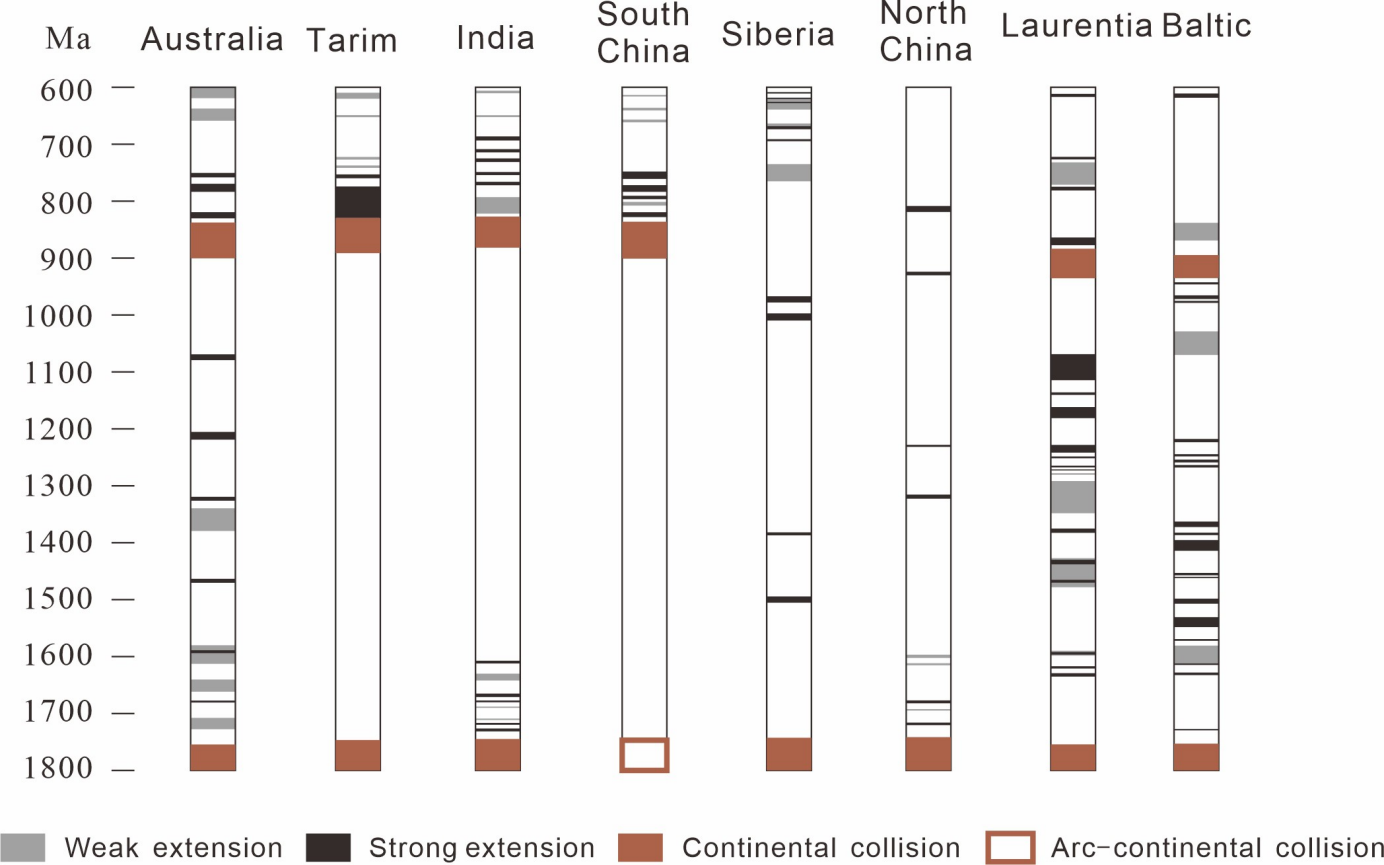
Comparison of thermo-tectonic events among Australia, Tarim, India, South China, Siberia, North China, Laurentia and Baltic between 1.8 Ga and 0.6 Ga (modified after [20, 46–48]).

The geological affinity of plates is very important in reconstruction of global plate tectonic configuration. Apparently, plate tectonic activities were not confined to Tarim and its surrounding areas, which were common in other plates [14, 23, 25, 42]. It should be of consideration whether there were connections among these plates. Firstly, according to the thermo-tectonic events comparison between Tarim, NCC, South China Craton (SCC) and other cratons all over the world (Fig 4), it could be noticed that Tarim and SCC were more related to India before 0.9 Ga, but Tarim and SCC were more related to Australia as one part of Gondwana supercontinent after 0.9 Ga [20, 27, 43–45].

**Fig 5:**
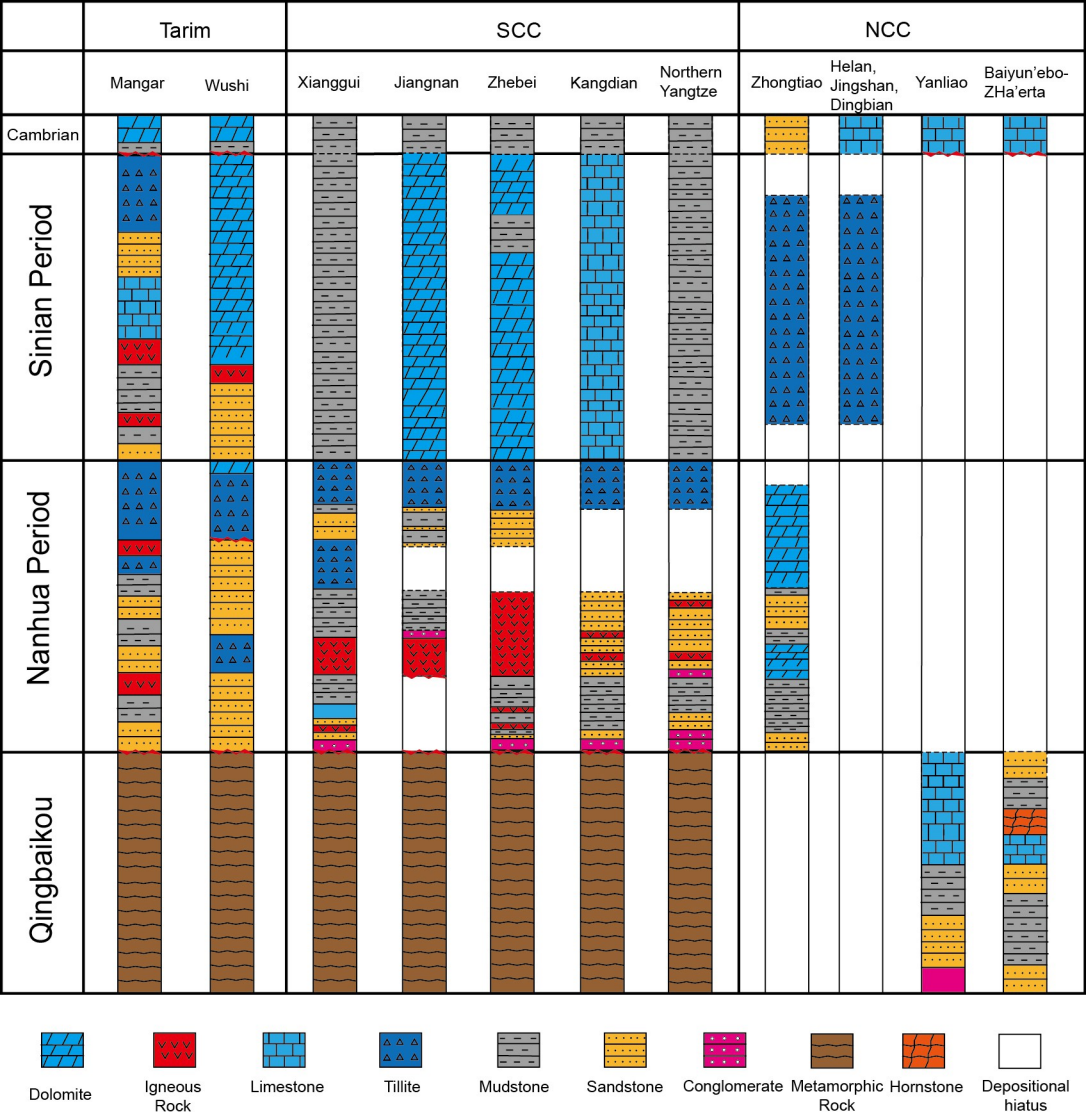
Precambrian strata of Tarim Craton, South China Craton (SCC) and North China Craton (NCC) (modified after [41, 48]).

Apart from the thermo-tectonic events, the plate affinity between Tarim, SCC and NCC through strata are further compared. Aligning the stratigraphic histogram (Fig 5), it is noticeable that Tarim Basin and SCC shared a greater affinity: they both developed Neoproterozoic strata sequence of rifts [43, 49–52]. NCC was quite different, developing Mesoproterozoic strata sequences of rifts but lacking Neoproterozoic sequences [43, 53, 54]. This is embodied further by, in Tarim and SCC, similar strata were developed in similar periods: igneous rock developed in the late Nanhua Period and the late Sinian Period, tillite developed in early Nanhua Period, and glacier developed in the early Sinian Period [43, 49].

**Fig 6:**
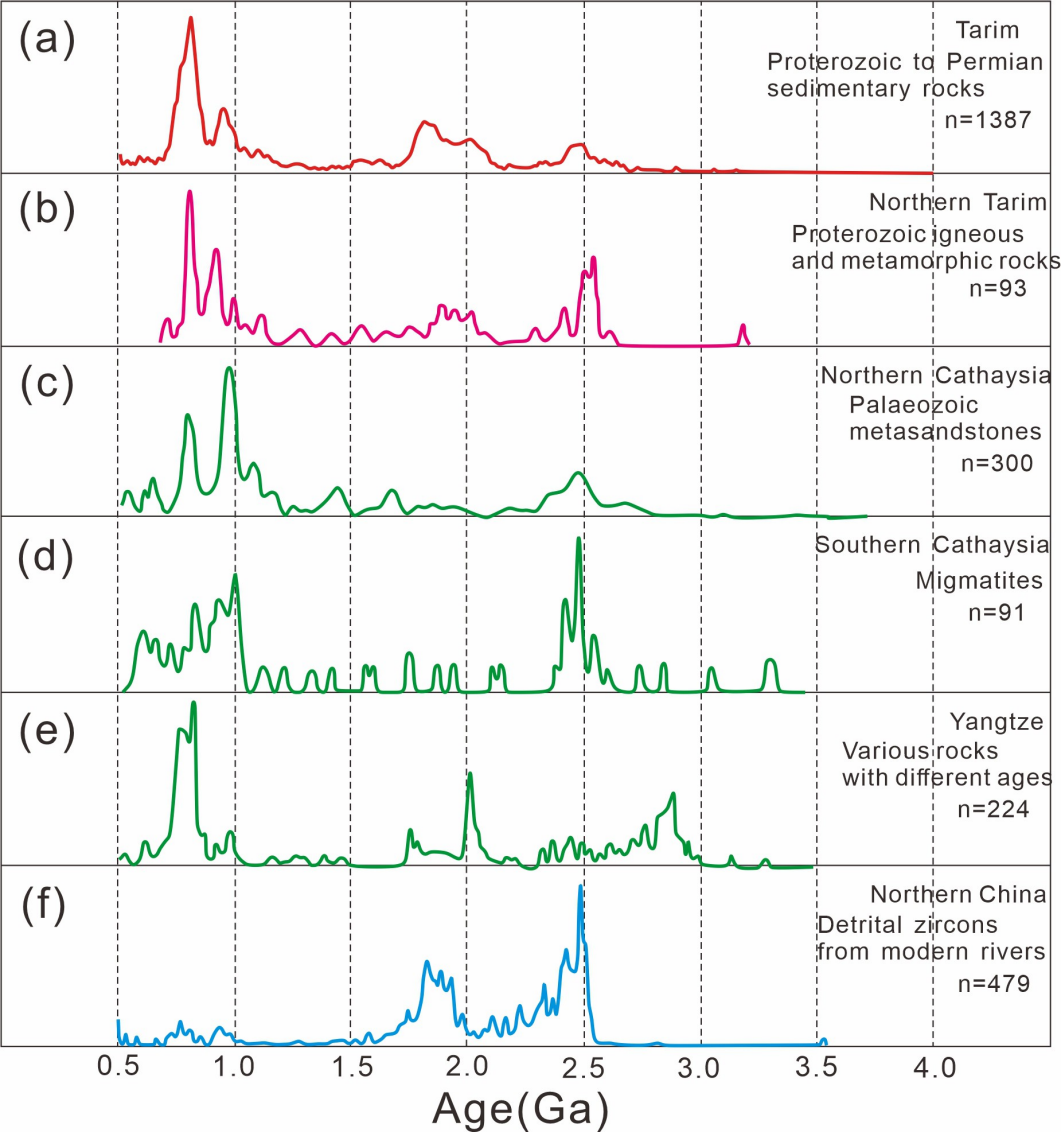
Detrital zircon age spectra of Tarim, Northern Tarim, South and North Cathaysia, Yangtze and Northern China since 4.0 Ga (modified after [43]).

The geological affinity between main cratons in Neoproterozoic can be proved by detrital zircon age spectra as well (Fig 6). The thermo-tectonic events in Tarim, Northern Tarim, Northern Cathaysia, Southern Cathaysia and Yangtze were synchronous before 1.0 Ga, especially during ca. 1.5–2.5 Ga. However, the zircon age population of NCC was totally different from the other five, which also implies Tarim shares a great affinity with SCC but not NCC [5, 43, 55].

Once straightening up the geological affinity among those plates, Tarim block was assumed to locate near the middle and low latitudes which were favorable for hydrocarbon generation. More precisely, Tarim block was near the equator (~27°) ca. 0.6 Ga based on paleomagnetic data [56], close to Australia as one part of Gondwana supercontinent [39]. The rifts in southwest of Tarim and Australia were of quite similarity, implying a close relationship between southwestern Tarim and Australia [18]. It is worth mentioning that the NCC was near Siberia at that time [18, 57–59].

According to previous studies on the spatial distribution of Rodinia supercontinent [7, 19, 20, 28, 29, 60–64], India, Tarim, Australia, SCC and East Antarctica were considered as the western group, while Laurentia, Baltic, Siberia and NCC as the eastern group of paleocontinents [44, 65, 66]. A super mantle plume was developed under the western group of paleocontinents, whose core was located in the junction of Australia, SCC and East Antarctic [39, 67–74].

**Fig 7:**
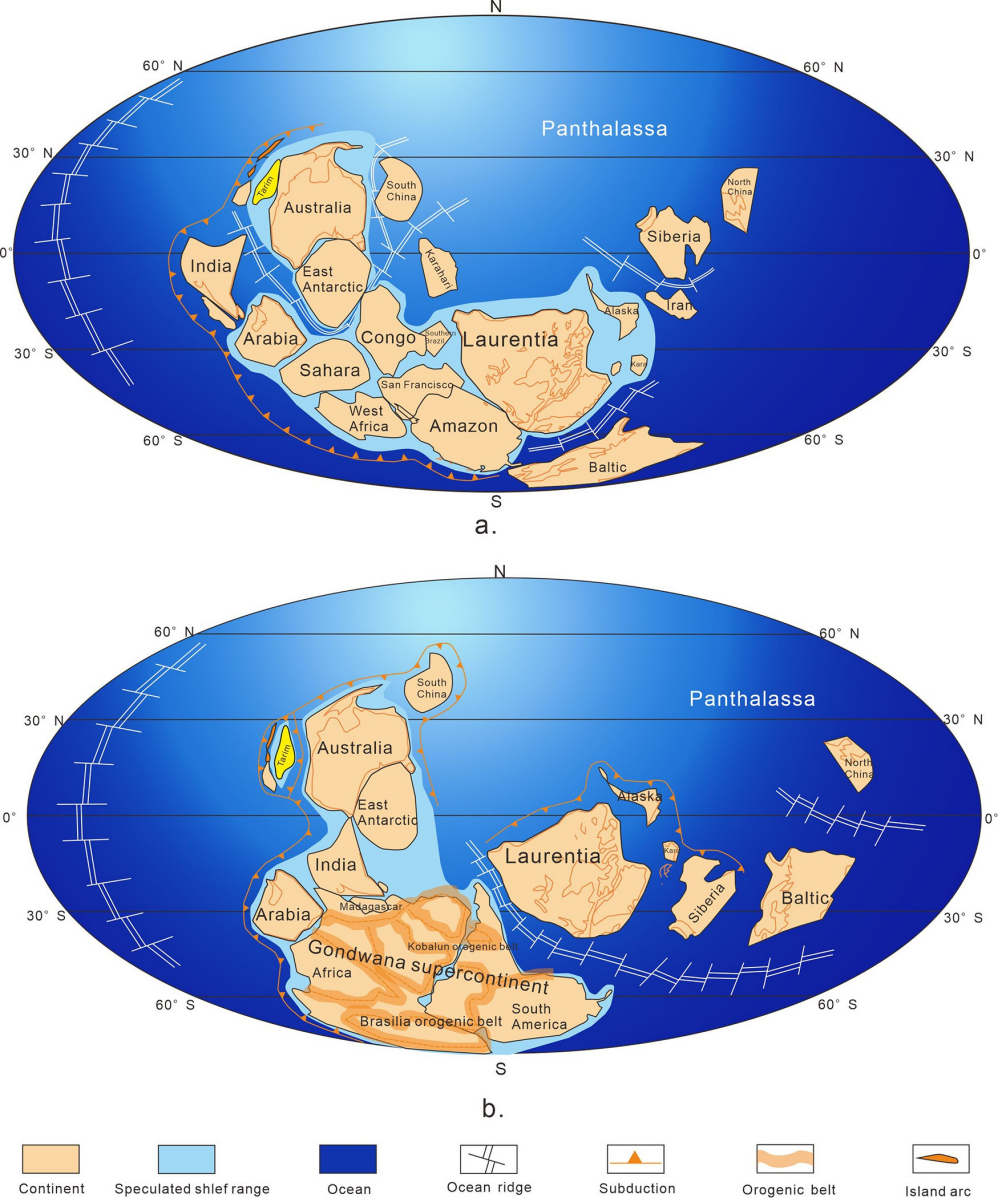
Reconstructing of global plates distribution in the Nanhua Period (a) and Sinian Period (b) (modified after [48]). Republished from Hou et al. (2008) under a CC BY license, with permission from *Gondwana Research*, original copyright 2008.

Based on abovementioned data and geological affinity, a new model of global plates configuration at ca. 0.6–0.8 Ga (the Nanhua Period and the Sinian Period) (Fig 7) is proposed. The long axis of Tarim is also adjusted to coincide with the rift sequence.

## 3. Database and methods

### 3.1 Database

To figure out the evolution of Tarim Basin and attain an overview of the tectono-paleogeographic framework of its adjacent area in the late Neoproterozoic, it is necessary to reconstruct the proto-type basin and tectono-paleogeographic maps. When it comes to Tarim Basin and its surrounding plates in the Precambrian, numerous investigations have been conducted such as (1)distribution of subbasins inside Tarim and their evolution [3, 12, 23, 32, 75–79], (2) paleogeographic distribution in Australia and Kazakhstan [80–83], (3) evolution history of Rodinia assembly and breakup in the late Neoproterozoic and the global plates pattern [15, 20, 48, 84–88]. The data, including thermo-tectonic events, lithostratigraphic sequences and deformation amounts of the basin range since Cenozoic [10], are collected and synthesized in this paper. In addition to these previously published data, the residual strata thickness, 30 seismic cross-sections and data from exploration wells and outcrops are attained, which could give a more sophisticated proto-type basin distribution.

### 3.2 Methods

#### (1) Restoration of proto-type basin

There are three principles we need to obey when reconstructing the proto-type basin maps. Firstly, reconstruct its location, which means figuring out the paleogeographic position. Secondly, reconstruct the isopach maps of proto-type basins and the distribution of sedimentary lithofacies. Finally, reconstruct the original range of the proto-type basin by calculating the deformation amounts. Detailed procedures are explained as follows.

1. Based on the residual strata thickness from Bureau of Geophysical Prospecting INC., according to the characteristics of different basins, restore the intact isopach map. For a marginal basin, the isopach should be open, outward and asymmetric. While for an intra-cratonic basin, the isopach should be concentric and closed. If encountering a platform or a surficial sea, then the isopach should be symmetrical but with openings connected to the shelf or marginal sea. For other kinds, their isopach maps should also be restored in accordance with their characteristics.
2. The deformation amounts are measured from the late Nanhua Period and the late Sinian Period to Cenozoic. Utilizing 20 seismic cross-sections in Tarim Basin in the late Nanhua Period and the late Sinian Period, the balanced geological transects are restored for calculating the deformation amounts. After obtaining the deformation amounts along the transects, the data are assigned to these four measuring lines (ML1, ML2, ML3, ML4) to get a weighted average uniformly distributed inside Tarim Basin (Fig 1). Combining the deformation amounts since Cenozoic from [10], we figure out the actual deformation amounts of Tarim Basin since the late Nanhua Period and the late Sinian Period along those four measuring lines. Since the deformation mainly existed along the margin of Tarim Basin, the deformation amounts are allocated mainly in the northern and southern margins (Table 1).
3. The lithofacies distribution is drawn according to the isopach map, wells and outcrops. When the lithofacies obtained from wells and outcrops contradict the isopach maps, take the former as correct. Replendish the surrounding plate environments according to the literature investigated before and then we obtain the proto-type basin maps in the late Nanhua Period and the late Sinian Period.

In order to get the tectono-paleogeographic maps, we rotated Tarim Basin to fit its paleogeographic distribution, then added a rough lithofacies distribution of Tarim Basin and its adjacent plates obtained from investigations before.

**Table 1:** The elastic properties used in this model.

Material	E(GPa)	*ν*
Faults	10	0.40
Deposited areas	70	0.27
Crystalline basement	80	0.25

#### (2) Numerical modelling

To study the mechanisms of the rift evolution inside Tarim Basin in the late Precambrian, we developed a three-dimensional elastic FEM model that includes all faults, deposited areas and its Archean-early Neoproterozoic metamorphic crystalline basement.

This model was conducted using COMSOL Multiphysics 6.1. It is 1395 kilometers long, covering the whole unified Tarim block, and 10 kilometers deep, which is the depth of the brittle upper crust layer, prone to rupture. There are three materials used in this model: faults, deposited areas and metamorphic crystalline basement, whose properties are summarized in Table 1. As describing elastic rock deformation in this model, the mechanical behavior here is controlled by Hooke’s law:

$$\varepsilon_{x}=\frac{1}{E}(\sigma_{x}-\nu \sigma_{y}-\nu \sigma_{z})\;\gamma_{xy}=\frac{2(1+\nu )}{E}\tau_{xy},$$

$$\varepsilon_{y}=\frac{1}{E}(\sigma_{y}-\nu \sigma_{x}-\nu \sigma_{z})\;\gamma_{yz}=\frac{2(1+\nu )}{E}\tau_{yz},$$

$$\varepsilon_{z}=\frac{1}{E}(\sigma_{z}-\nu \sigma_{x}-\nu \sigma_{y})\;\gamma_{xz}=\frac{2(1+\nu )}{E}\tau_{xz},$$

where E is the Young’s modulus, *ν* is the Poisson’s ratio, *σ* and *ε* represent the main stress and strain, *γ* and *τ* represent the shear stress and strain.

Besides the basic properties of the elastic model, the mantle upwelling was applied vertically on the bottom of the model with a magnitude of ~75 MPa [89, 90], and the subduction was considered to be applied along the northern margin of Tarim block with a magnitude of ~100 MPa [89, 90].

## 4. Results

### 4.1 Shortening amount of Tarim Basin in the late Precambrian

This study recovers 10 profiles to get a more precise deformation amounts of Tarim proto-type basin from the late Nanhua Period and the late Sinian Period to Cenozoic. The deformation amounts of Tarim Basin from Cenozoic to present are obtained from Laborde et al. [10].

**Fig 8:**
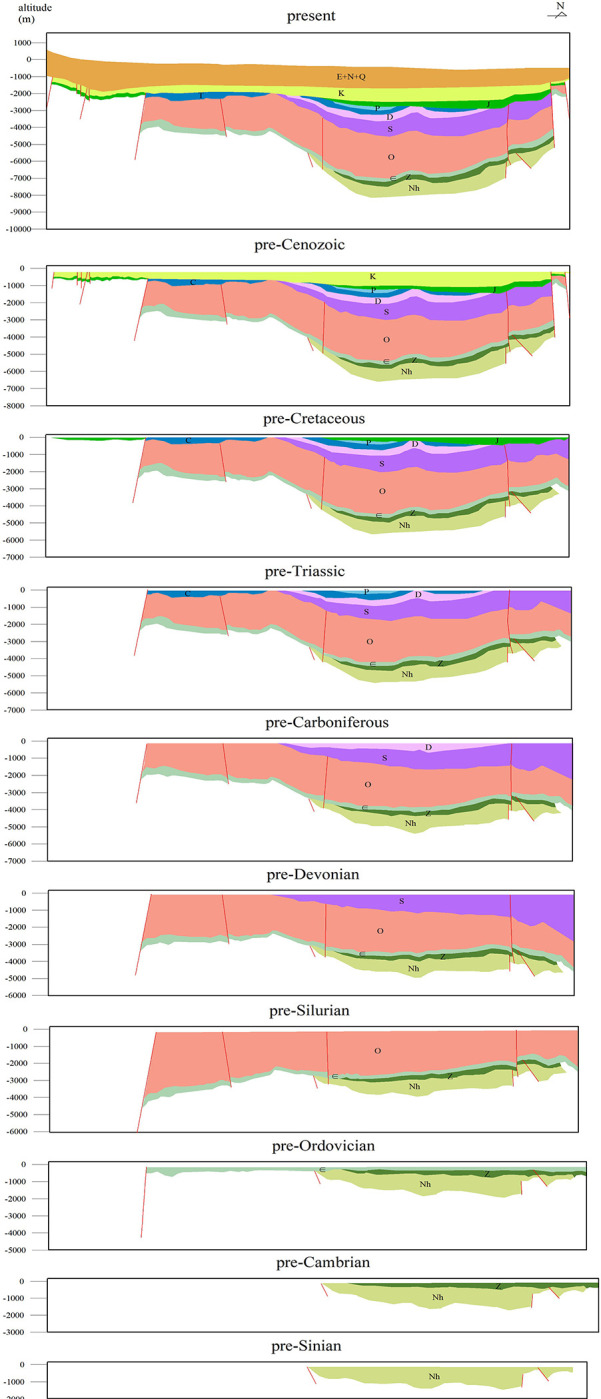
Restored balanced geological transect of NS21 from an already interpreted seismic profile.

2D-MOVE, a software designed to restore the balanced geological transects particularly, was used to restore the 10 seismic profiles. During this process, linear balance was chosen as the principle to restore the balanced geological transects (Fig 8) and further calculate the deformation amounts.

Since the deformation amounts were obtained directly in the process of restoration in 2D-MOVE, the uneven distribution of the data needs to be reallocated to make them more even. As the deformation mainly happened around the margin rather than inside the basin, this study attributed the deformation amounts to four evenly distributed measuring lines in Tarim Basin (Fig 1) came up by Laborde et al. [10], to display the deformation amounts reasonably. The deformation amounts of these four measuring lines were obtained by assigning more weight to its nearby data and less weight to its distant data to get a weighted average (Table 2).

**Table 2:** Shortening amounts of Tarim Basin between the Cenozoic, Sinian, Nanhua Periods and present (/km).

Distribution of the shortening amount	Cenozoic (Laborde et al., [10])	Sinian Period (This study)	Nanhua Period (This study)
Northern margin of ML1	36.0	46.0	42.0
Southern margin of ML1	32.0	42.0	40.0
Northern margin of ML2	21.0	43.0	39.0
Southern margin of ML2	35.0	41.0	40.0
Northern margin of ML3	22.0	36.0	33.0
Southern margin of ML3	0.9	10.9	7.9
Northern margin of ML4	0.0	17.0	9.0
Southern margin of ML4	0.3	23.3	16.3

### 4.2 Reconstruction of proto-type Tarim Basin in the late Precambrian

#### (1) Proto-type Tarim Basin in the late Nanhua Period

The bimodal volcanic rocks and the dike swarms in the Nanhua Period (before ca. 735 Ma), found in Yecheng, southeast of Tarim, indicated the existence of a pre-rift stage of continent rifting [23]. The blueschist in Aksu also indicated a deep subduction in the northwest margin of Tarim [1, 37], while NW-trending dike swarms indicated an extension after the subduction in the northwest of Tarim [28, 29].

**Fig 9:**
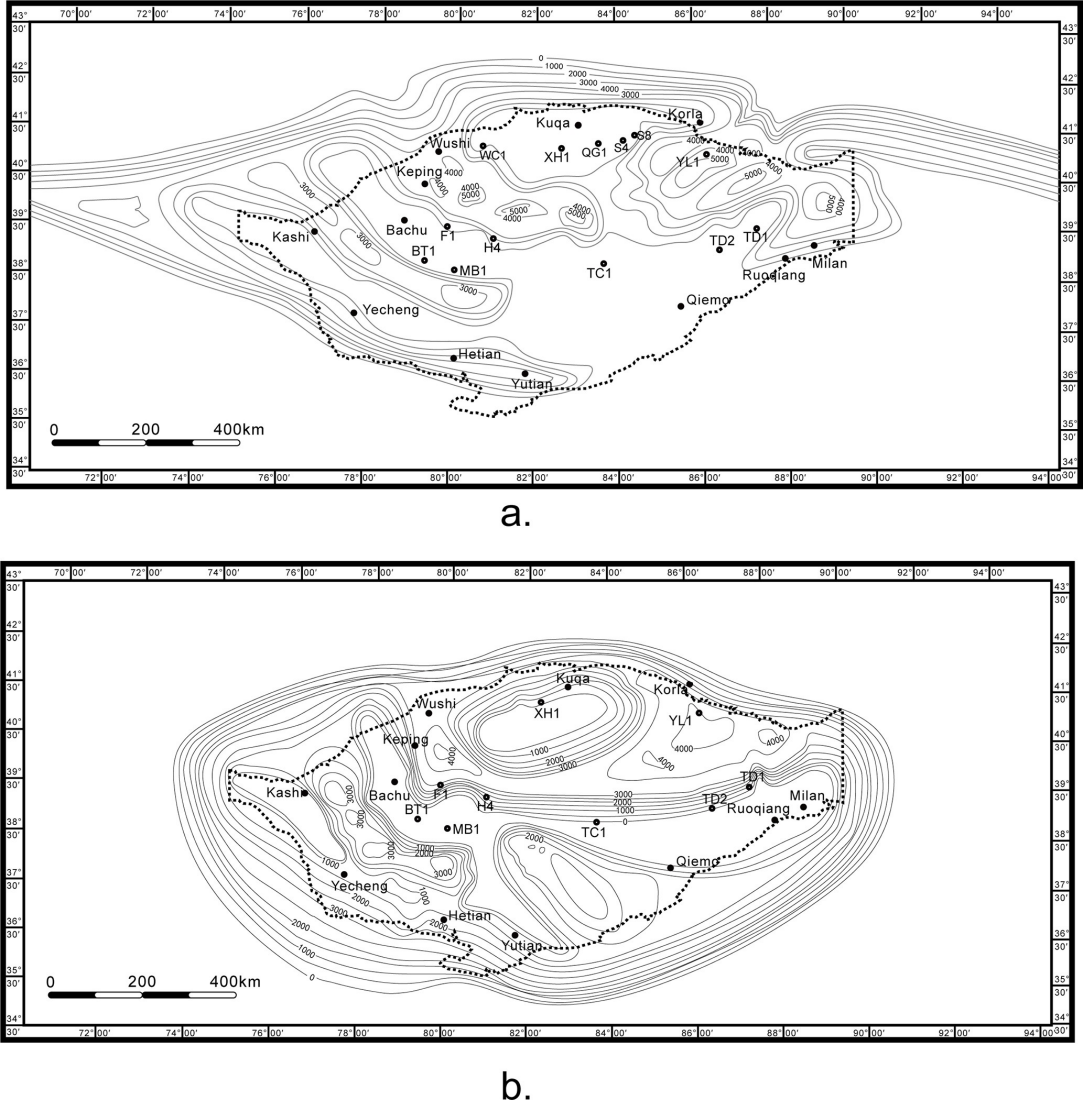
Residual strata thickness of Tarim Basin in the late Nanhua Period (a) and the late Sinian Period (b). Republished from Zhong et al. (2023) under a CC BY license, with permission from *Frontiers in Earth Science*, original copyright 2023.

Since the Nanhua Period, the rifts in Tarim Basin had developed further due to the breakup of Rodinia supercontinent and the subduction of Rodinia Ocean. In the Nanhua Period, Tarim Basin mainly developed sedimentary centers in Quruqtagh, Aksu and Tiekelike (known as Hetian and Yecheng) (Fig 9A). In detail, during the Nanhua Period, the azimuth distribution of the rifts in Quruqtagh showed a predominant NE-SW trend, with the depth becoming smaller towards central Tarim Basin thus narrowing the coverage. The rifts in Aksu trended mainly NW-SE, sharing a similar pattern to those in Quruqtagh. The pattern of rifts in Tiekelike was similar too, except that they trended SW-NE.

**Fig 10:**
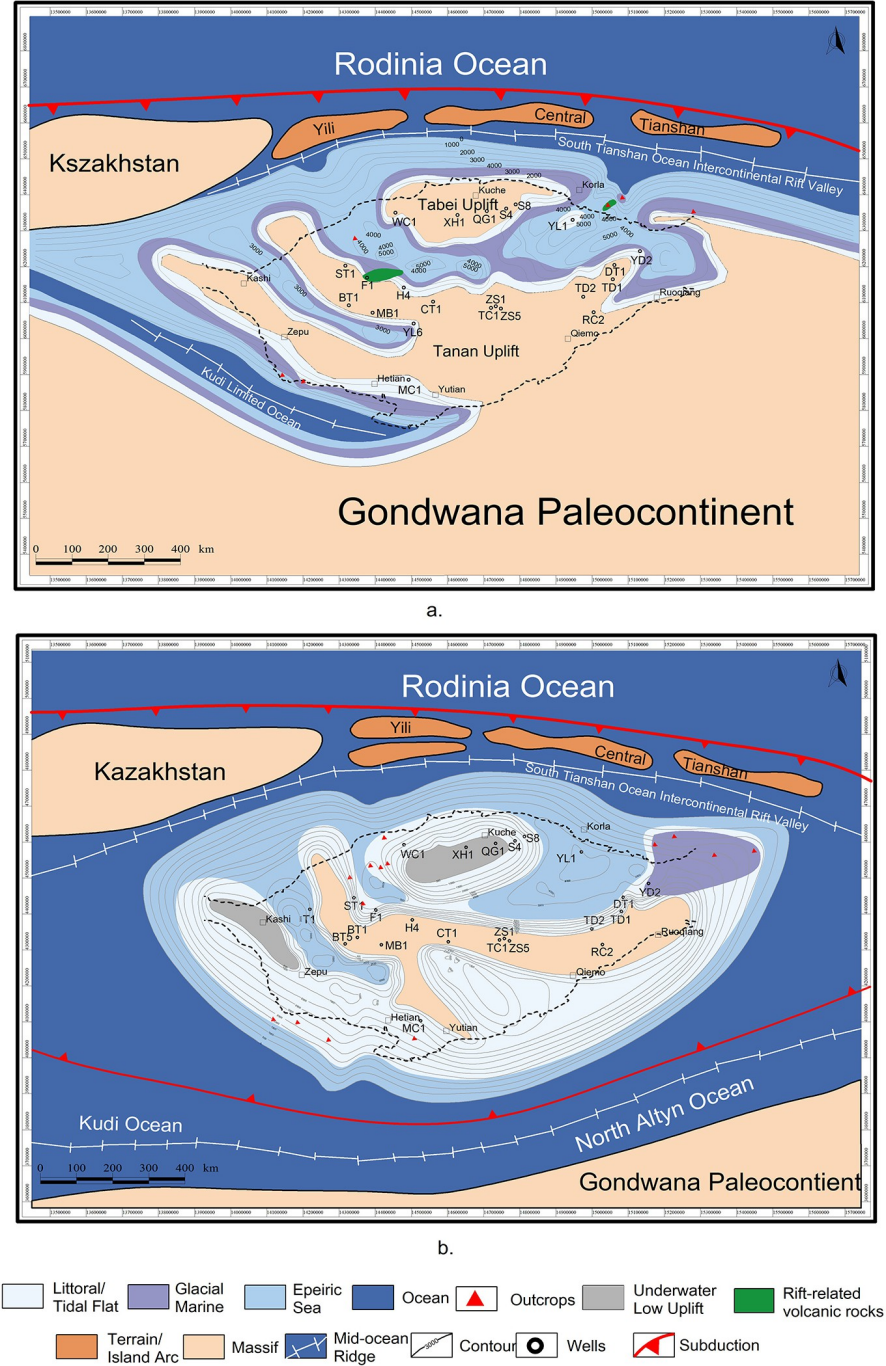
Proto-type basin of Tarim and adjacent areas in the late Nanhua Period (a) and Sinian Period (b). Republished from Zhong et al. (2023) under a CC BY license, with permission from *Frontiers in Earth Science*, original copyright 2023.

In the early Nanhua Period, the periphery of Tarim Basin was composed mainly of shallow marine and semi-deep sea, accompanied by a small amount of glacial marine and volcanic rocks in the rift valley (Fig 10A). Then in the late Nanhua Period, deltas have emerged. Correspondingly, glacial-marine and shore have dominated a bigger area [11].

#### (2) Proto-type Tarim Basin in the late Sinian Period

The pattern of rifts distribution in the Sinian Period was inherited from the Nanhua Period, and thus was similar except that these rifts were beginning to shrink. However, the uplift-subsidence pattern of Tarim Basin has changed in the late Sinian Period. Compared with the late Nanhua Period, Tarim Basin in the Sinian Period has transformed from a pattern of two uplifts and one depression into one uplift in the south and one depression in the north. The northern Tarim became a low uplift underwater due to glacier melting [91].

**Fig 11:**
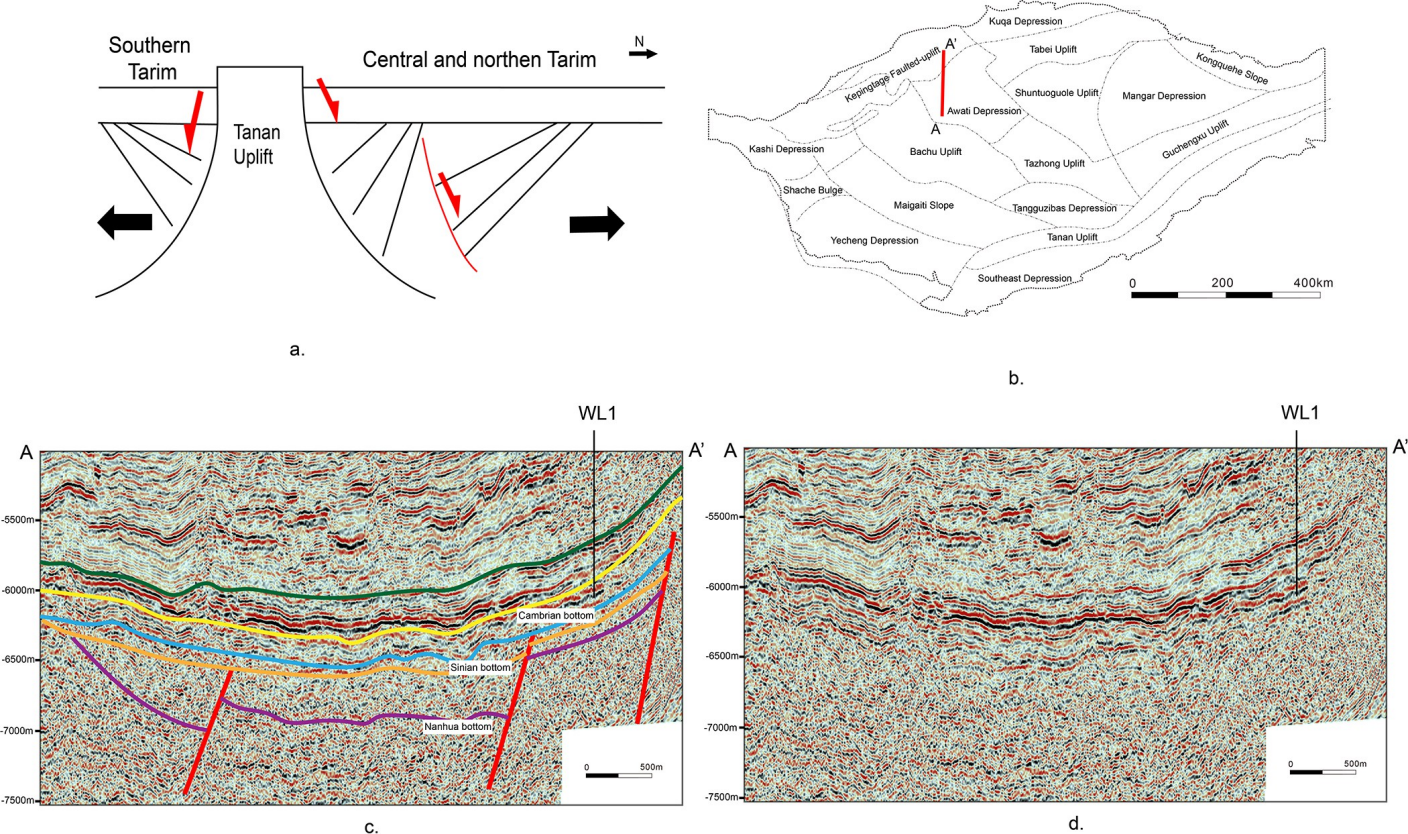
Parallel unconformity between the Cambrian and Sinian Period: (a) An ideal model of explanation for differential uplift and subsidence, (b) Location of the seismic profile, (c) Seismic profile showing a similar pattern to the ideal model, A-A’ is the same as which in (b), (d) Original seismic profile of (c). Republished from Zhong et al. (2023) under a CC BY license, with permission from *Frontiers in Earth Science*, original copyright 2023.

The uplift-subsidence pattern was not only limited to being caused by orogenic uplift, but also could be caused by extensional activities [4, 49, 92]. The angular unconformity may indicate orogenic events, where underlying folds, reverse faults, compression shortening and metamorphism occur. However, the angular unconformity would indicate rifting and extension if there are underlying semi-grabens, overlapping and positive growth faults but without folds. The boundary between Cambrian and Sinian Period was obvious, while the boundary between Sinian and Nanhua Period, and the boundary between Nanhua and the basement were both obscure. Besides, rifts were widely developed in Nanhua and Sinian Period. As a result, the lowest obvious boundary with rifts underlying was recognized as the boundary between Cambrian and Sinian Period. According to the contact relationship between the upper and lower strata of Cambrian bottom boundary in Tarim Basin, it can be found that the majority of the contact relationship between the Lower Cambrian and the Upper Sinian Period wass parallel unconformity (Fig 11C). Combined with other data, it can be found that the parallel unconformity has inherited lithofacies and thickness from its neighboring strata without strong fold or metamorphism [93], thus should have nothing to do with orogenic events. Based on the above, a model accounting for the uplift-subsidence pattern is put forward (Fig 11A), fitting for the stratigraphic contact relationship interpreted from the seismic profile well (Fig 11C).

To sum up, it can be conducted that the expansion around the northern margin of Tarim in ca. 540 Ma led to differential uplift in the middle Tarim Basin, and formed local angular unconformity. Besides, the tectonic setting of Tarim Basin in the Nanhua Period and the Sinian Period was extensional, which directly contradicted orogenic events. Therefore, the uplift-subsidence pattern in Tarim Basin during the Nanhua Period and the Sinian Period was not caused by Pan-African orogenic events [18, 94], but formed by extension events related to the breakup of Rodinia supercontinent.

In the early Sinian Period, deltas further developed, increased, and gradually connected with each other. Glacial marine retreating, the periphery of Tarim Basin displayed as shallow marine [11]. In the late Sinian Period, deltas submerged as well. Tarim Basin was composed mainly of littoral and tidal flat zones (Fig 10B), which responded to the shrinking stage of rifts.

It is also worth noticing that the glacial marine was concentrated in the eastern Tarim Basin. For the polarity of Tarim Basin in the Precambrian had a difference of 100 degrees from that at present [41], thus the east of present Tarim Basin was to the north in the Sinian Period. After the Snowball Earth, glaciers melted and ice sheets retreated to the north, leaving glacial-marine concentrated in the high-latitude area then.

### 4.3 Reconstruction of tectono-paleogeography around Tarim Basin in the late Precambrian

**Fig 12:**
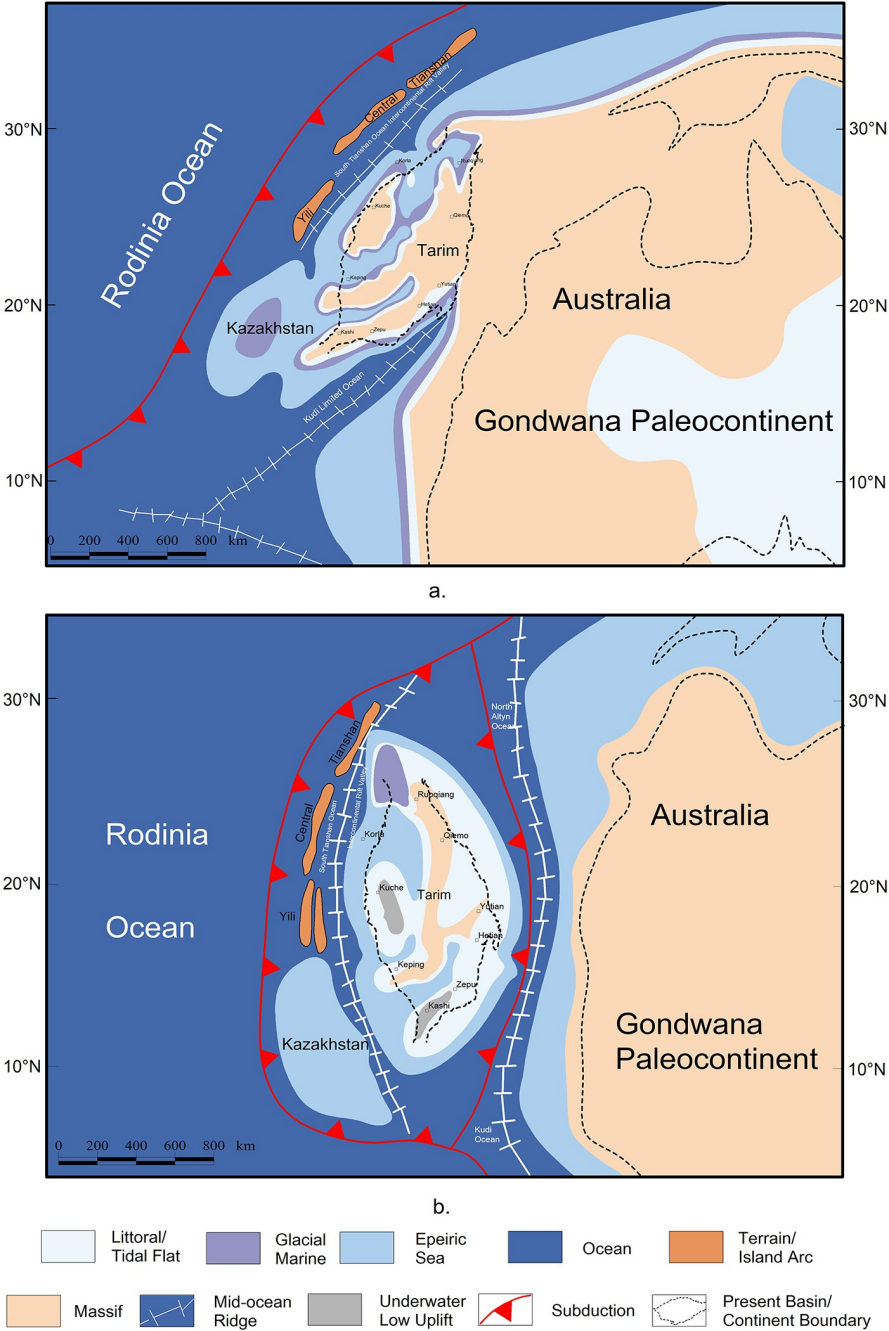
Tectono-paleogeography of Tarim and adjacent areas in the late Nanhua Period (a) and Sinian Period (b). Republished from Zhong et al. (2023) under a CC BY license, with permission from *Frontiers in Earth Science*, original copyright 2023.

Tectono-paleogeography is an important part of the research in multicycle superimposed sedimentary basins. Sorting out the Precambrian tectono-paleogeography evolution of Tarim Basin could offer an intuitive picture of how Tarim Basin and its adjacent area evolved and what drove the evolution (Fig 12).

#### (1) Tectono-paleogeography around Tarim Basin in the late Nanhua Period

In the Nanhua Period, Rodinia supercontinent was just beginning to break up, thus Tarim was not separated from Gondwana paleocontinent based on the reconstruction of proto-type basin and geological affinity between the southeast of Tarim Basin and Australia (Fig 12A). Around Tarim Basin, Kudi ocean on the southwest began to open [45, 74]. Due to the supercontinent breakup, there emerged a triple junction in the southwest of Tarim Basin. The three arms were two as Kudi ocean, which continued to develop and expand, and one as aulacogens extending to the southwest of Tarim Basin, which failed eventually [6]. In the north, the South Tianshan Ocean had just begun to expand, leaving a back-arc expansion environment in the northern margin of Tarim Basin, transforming into a limited ocean of back-arc intercontinental rifts [39].

In the late Nanhua Period, the whole planet was undergoing glacial events [60]. It can be inferred that there would be a certain range of glacial-marine and shallow marine distributed around Tarim Basin and its adjacent areas. Australia was roughly distributed as marine facies in the southeast and continental facies in the northeast and southwest [81]. Further, because of the breakup of Rodinia, rifts developed in the southeast of Australia, depositing shale and sandstone, showing littoral facies [80]. In the northwest, it was continental area where denudation developed. And outside the whole continent, glacial-marine was distributed.

There were occasionally magmatic arc volcanic rocks in Kazakhstan, but the stratigraphic sequence showed abundant limestone and tillite distribution [83]. In the Neoproterozoic, dolomite was formed in Lesser Karatau, while mixed sandstone and shale were deposited in Greater Karatau [82]. According to the distribution of the global lithofacies paleogeography map in Precambrian, the lithofacies of Kazakhstan at that time were all marine facies [18]. Therefore, it can be speculated that the more specific lithofacies distribution of Kazakhstan was shallow marine distributed outside while glacial marine distributed where tillites were formed.

#### (2) Tectono-paleogeography around Tarim Basin in the late Sinian Period

In the late Sinian Period, Kudi ocean in the southwest continued to develop. In the meantime, Tarim Basin finally broke up with Gondwana paleocontinent in the southeast. The Altyn ocean began to open, and connected with Kudi ocean [45, 74]. In the north, the south Tianshan ocean continued to open, and finally ran through the northern margin of Tarim Basin (Fig 12B).

Australia displayed a similar lithofacies distribution to that in the Nanhua Period. However, the shallow marine had expanded towards northwest. It was related to the cessation of the rift development and the beginning of deposition, as well as the melting of glaciers [81].

As for Kazakhstan, due to the melting of glacial marine, it was characterized by shallow marine completely [18, 83].

In the late Sinian Period, glacial marine had already retreated. The breakup of Rodinia supercontinent had also finished. It can be inferred that the range of glacial marine had decreased and the shallow marine expanded a lot.

### 4.4 Paleotectonic stress filed of Tarim Basin in the late Nanhua Period

**Fig 13:**
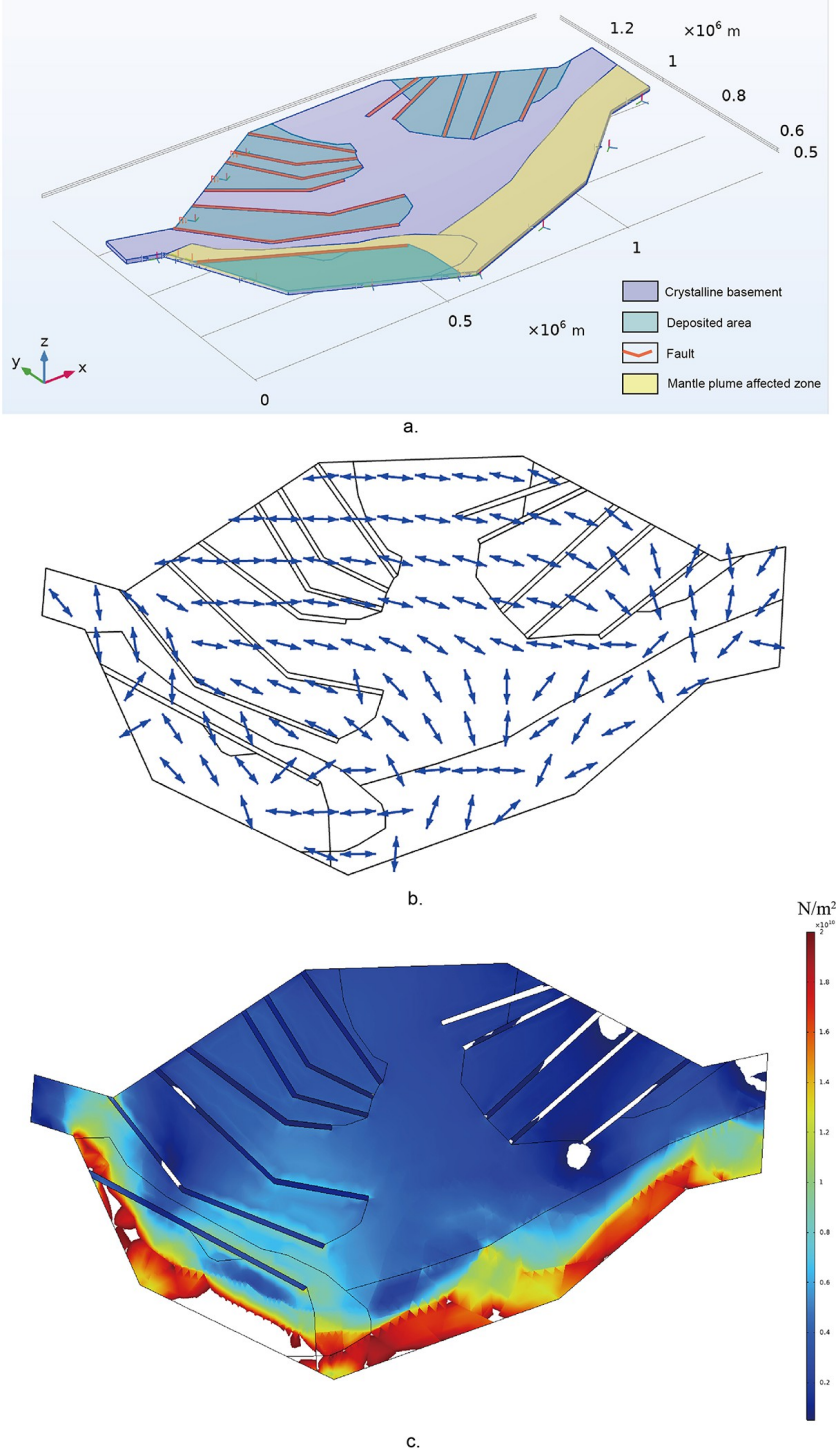
Paleotectonic stress filed of Tarim Basin in the late Nanhua Period: (a) main view of the 3-D model; (b) tensile stress trajectories map of Tarim block in the late Nanhua Period; (c) differential stress field map of Tarim block in the late Nanhua Period.

Given the abovementioned three-dimensional elastic model, we applied the boundary conditions on the model and calculated its results from a free beginning to get a best-fit model (Fig 12A). The horizontal tensile stress trajectory map and the differential stress field map of Tarim Basin in the late Nanhua Period are obtained and presented in Fig 13. The horizontal tensile stress trajectory map is obtained by projecting the 3-D tensile stress of the surface of the model to a horizontal plane.

From the horizontal tensile stress trajectory map (Fig 13B), the trajectories of the tensile stress in the three main rift zone are all consistent with the opening direction. In the northeastern Tarim, the trajectories of the tensile stress are NW-SE trending, which is consistent with the opening direction of the rifts there as can be seen in the map. In the northwestern Tarim, the trajectories show a NE-SW trend, and in the southwest, the trajectories trended NE-SW. They both conform to the opening direction of the rifts.

And in the differential stress field map (Fig 13C), apart from the systematic stress intensity anomaly caused by the boundary conditions applied to the model, the main stress concentration areas are located in the southern margin and alone the rift zone in the northwestern Tarim.

## 5. Discussion

### 5.1 Characters of rifts inside Tarim Basin in the late Precambrian

In Aksu, blueschist formed during 800–754 Ma [37] indicates a deep subduction in the northwestern margin of Tarim Basin as mentioned before. The Ar-Ar age of the hornblende from basaltic dike swarms was 759–744 Ma [73], which were formed in an extension environment immediately after the subduction. Therefore, rifts during the Nanhua Period in northwestern Tarim Basin were highly likely to be back-arc rifts experiencing a complex geodynamic evolution from subduction to back-arc extension [14, 32, 52, 95], rather than aulacogens caused by the mantle plume.

Ophiolites of about 800 Ma ago were developed in Quruqtagh area, the northeastern Tarim Basin [96]. In addition, rhyolites and associated syenite granites existed during Nanhua Period, of which U-Pb ages of zircons were 735.4 ± 10 Ma and 738.9 ± 5.4 Ma respectively, which means they were products of Neoproterozoic igneous activities during Nanhua Period [97]. These products show the characteristics of A-type granites [28], indicating that they were generated in a background of tectonic transformation from subduction to extension, which was rightly in response to the breakup of Rodinia supercontinent [98, 99]. Also, basaltic dike swarms were also widely exposed in this area, and the average U-Pb age of zircons from diabase dikes nearby was 773 ± 3 Ma [100, 101]. U-Pb ages of zircons from granodiorites and K-feldspar granites in Quruqtagh were around 630 Ma [22], indicating that the northeastern margin of Tarim also experienced the same geodynamic evolution from subduction to extension as the northwestern Tarim block.

While referring to the southwestern Tarim Basin with close affinity to Australia and SCC that experienced rifting related to Rodinia superplume, substantial analyses have been conducted to research the evolution of the rifts. According to systematic zircon Hf isotope analyses, an 820–800 Ma deviation was noticed and thought to be highly likely due to the effects of Rodinia superplume [1, 14, 74, 102]. Apart from geochemistry, sedimentary sequences in Yutang area, southwest of Tarim Basin, represent a fluvial/alluvial-lacustrine-shallow marine tectonostratigraphic sequence, which is comparable with the deposition of sedimentary basin obeying the evolution path from syn-rifting, late-rifting, transitional to post-rifting stage according to basin analysis [52]. As a result, rifts in southwestern Tarim Basin were believed to be aulacogens caused by mantle plume related to the breakup of Rodinia supercontinent.

During the Nanhua Period, Rodinia supercontinent was just beginning to break up. Tarim block was not separated from Rodinia supercontinent yet. However, Tarim Basin was developing rifts due to the supercontinent breakup (Fig 10A). As analyzed before, there were three sedimentary centers then: Aksu, Quruqtagh and Tiekelike. They were all in pre- and syn-rift period [26, 91, 103]. These three rift sedimentary centers were all conducive to oil and gas generation. Tarim Basin as a whole formed a paleogeographic pattern of two uplifts in the southern and northern Tarim Basin, and one depression in the central Tarim Basin.

During the Sinian Period, Rodinia supercontinent almost finished its breakup, and Tarim block was separated from Gondwana [45, 74]. As a result, Tarim Basin has been affected less by the continental breakup, subduction, and expansion, so rifts no longer developed [15, 52, 104]. Rifts in Tarim Basin have entered the stage of shrinking. Sedimentary centers were developed only in Quruqtagh and Tiekelike. The deposition was ceased in Aksu because of the local uplift caused by extension event. The overall residual thickness in Quruqtagh became smaller, but covered a larger area. Rifts in Yecheng and Hetian were connected, with the residual thickness and coverage area showing a similar pattern to those in Quruqtagh. These two sedimentary centers still favored oil and gas generation [6, 52, 77], worth attention when exploring resources.

### 5.2 The dynamic mechanism of the evolution of the rifts in Tarim Basin in the late Precambrian

From the analysis above, a cognition can be reached that: the rifts in Tarim Basin were divided into two kinds, one in the northern margin (Quruqtagh and Aksu) and the other in the southern margin (Tiekelike). The former was caused by subduction, known as the Neoproterozoic Circum-Rodinia Subduction System [1, 3, 7, 13–15, 18, 21, 28, 36, 78, 104, 105], while the latter was caused by the mantle plume related to the breakup of Rodinia supercontinent [6, 18, 23, 24, 36, 51, 69, 106–108]. Combined with the location of Tarim block in the late Nanhua Period (Fig 7A), this situation can be understood more intuitively. The northern margin of Tarim block was further from the core of the Rodinia supercontinent compared to its southern margin, but close to the Circum-Rodinia Subduction System. Therefore, these two rifts system originated and developed out of two different dynamic mechanisms.

In order to explain this more solidly, the numerical model mention above should be considered. In this model, by applying the boundary conditions simplified from the two tectonic events, which are the southern mantle plume upwelling and the northern subduction, the opening directions of the rifts (Fig 13B) are shown as exactly like that this paper researched in the proto-type Tarim Basin map (Fig 10). As a result, the peripheral tectonic environment of Tarim block in the late Nanhua Period was able to drive the rifts inside Tarim Basin to open and develop as we researched from geological evidence.

Apart from the characteristics of the rifts in Tarim Basin, the differential stress concentration, which could reveal the areas prone to rupture, also coincides with the actual behaviors of Tarim block in the late Nanhua Period. As we can see in Fig 13C, the main stress concentration areas are located in the southern margin and along the rift zone in the northwestern Tarim. Tarim block was undergoing the breakup of the Rodinia supercontinent and was about to be separated from Australia from its southern margin. Thus, the differential stress concentration in the south could help explain this event. Also, the concentration along the rift zone in the northwestern Tarim could explain that, the rifts were more prone to open in the northwest rather than in the middle.

After the late Nanhua Period, the mantle plume upwelling disappeared. Naturally, the ancient stress field, conducive to the rift development, also disappeared. Therefore, in the Sinian Period, the rifts in Tarim Basin had begun to shrink, as shown in the proto-type Tarim Basin map (Fig 10B).

## 6. Conclusion

1. According to previous paleomagnetic research, Tarim block was at the low latitudes, near the equator in the Nanhua Period and Sinian Period, which is conducive to hydrocarbon development. Based on the geological affinity, this paper further narrowed the location of Tarim block in the Rodinia supercontinent in late Precambrian.
2. During 1.0–0.8 Ga, the north and south paleo-Tarim terranes had experienced subduction and collision to finally form a unified Tarim block, which is called Tarim Movement. The time of Tarim Movement was a little different from Grenvillian Movement (1.1–0.9 Ga), which formed the Rodinia supercontinent. The difference may be related to that Tarim block was at the periphery of Rodinia supercontinent rather than in the core.
3. From the Nanhua Period to the Sinian Period, Tarim Basin had experienced a sedimentary evolution from continental facies to marine facies, corresponding with the rift evolution from firstly developing to a peak stage but finally to depression. In the north, the South Tianshan Ocean had just begun to expand in the Nanhua Period, leaving the northern margin of Tarim block a back-arc expansion environment. In the south, Rodinia supercontinent was experiencing a breakup, leading Kudi Ocean and Altyn Ocean to open.
4. Rifts in Tarim Basin were divided into one back-arc basins in the northern margin (Quruqtagh and Aksu) and the other aulacogens in the southern margin (Tiekelike). Combined numerical modelling, the dynamic mechanisms of the evolution of these two rift systems were figured out. The former was believed to be caused by subduction, which is known as the Neoproterozoic Circum-Rodinia Subduction System, while the latter was caused by mantle plume related to breakup of the Rodinia supercontinent.

## Supporting information

S1 File Interpretation of data. (DOCX)

S2 File 10 balanced geological transects this study restored. (ZIP)

S3 File 20 balanced geological transects from Tarim Oilfield Company. (ZIP)

S4 File 10 original seismic profiles. (ZIP)
