# Supplementary material for: Analysis of proto-type Tarim Basin in the late Precambrian and the dynamic mechanism of its evolution
Source: PLoS One. 2023 Jun 7;18(6):e0286849. doi: 10.1371/journal.pone.0286849 (PMC10246823; doi:10.1371/journal.pone.0286849)
Supplement: S1 File — (DOCX) [file pone.0286849.s001.docx]

Interpretation of Data

The shortening/extension amount of Tarim proto-type basin is calculated by the restoration of balanced geological transects.

There are **20 restored balanced geological transects (Fig. 1 for example) obtained from the Tarim Oilfield Company** for us to consult after Silurian. These data were each used to calculate the subsequent shortening/extension amount and to consult to make sure the amount was reasonable, and abandon some unreasonable data.


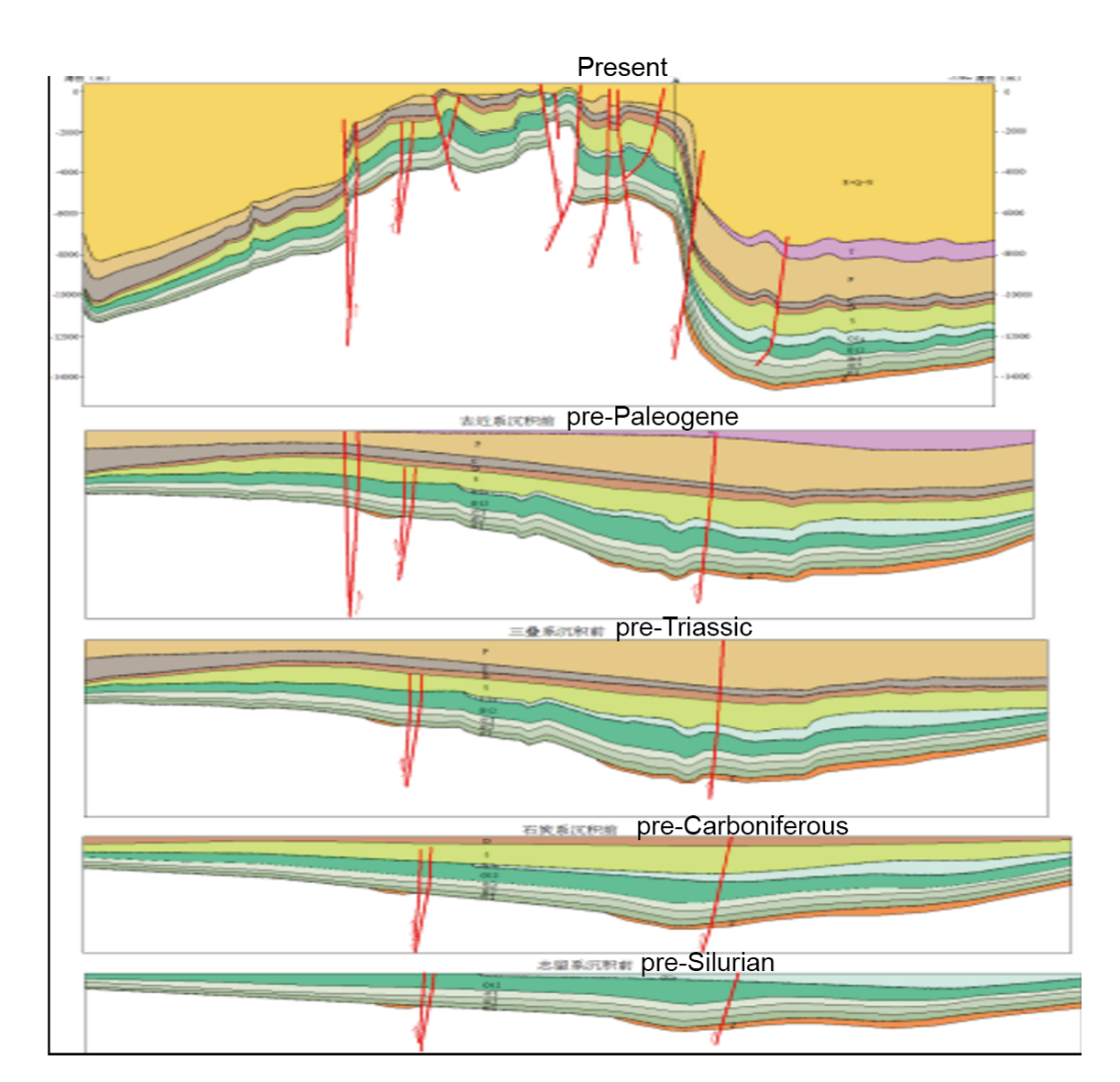


Fig. 1. Balanced geological transect 1 from Tarim Oilfield Company

When it comes to the Precambrian, due to the lack of Precambrian periods restorations in some of these profiles, this study recovers 10 profiles in extra to get a more precise shortening/extension amount of Tarim proto-type basin from the Nanhua Period and Sinian Period to Cenozoic. The shortening/extension amount of Tarim Basin from Cenozoic to present are obtained from Laborde et al., (2019).


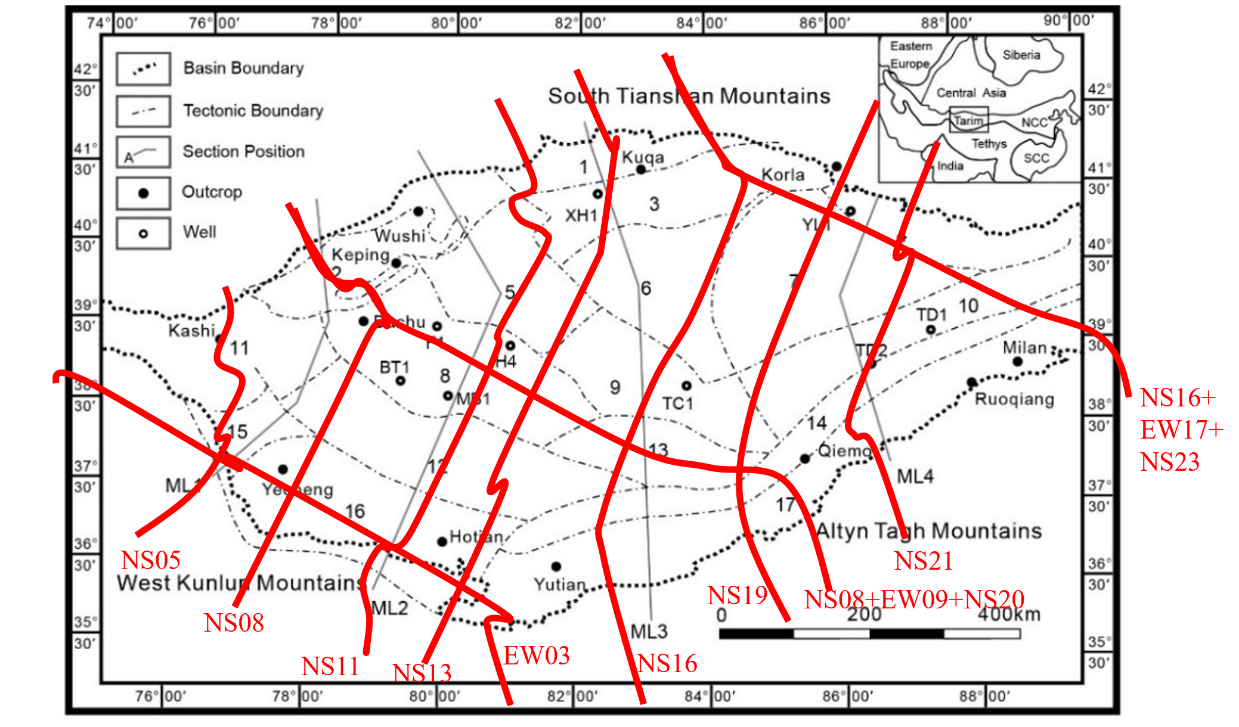


Fig. 2. Distribution of the 10 profiles we chose

We use 2D-MOVE, a software designed to restore the balanced geological transects particularly, to **restore the 10 seismic profiles we chose (Fig. 3 for example)**. During this process, linear balance was chosen as the principle to restore **the balanced geological transects (Fig. 4)** and further calculate the shortening/extension amount.


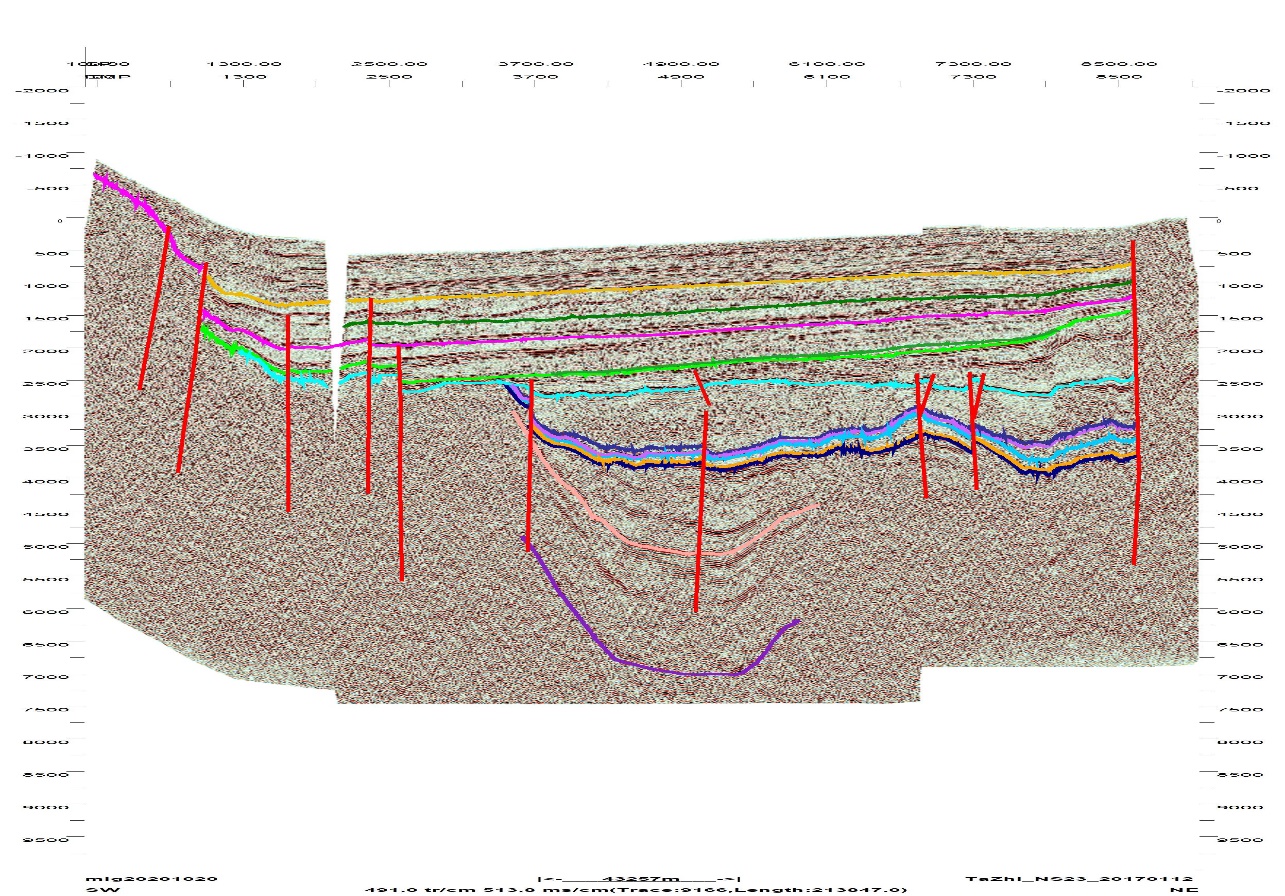


Fig 3. Seismic profile of NS21


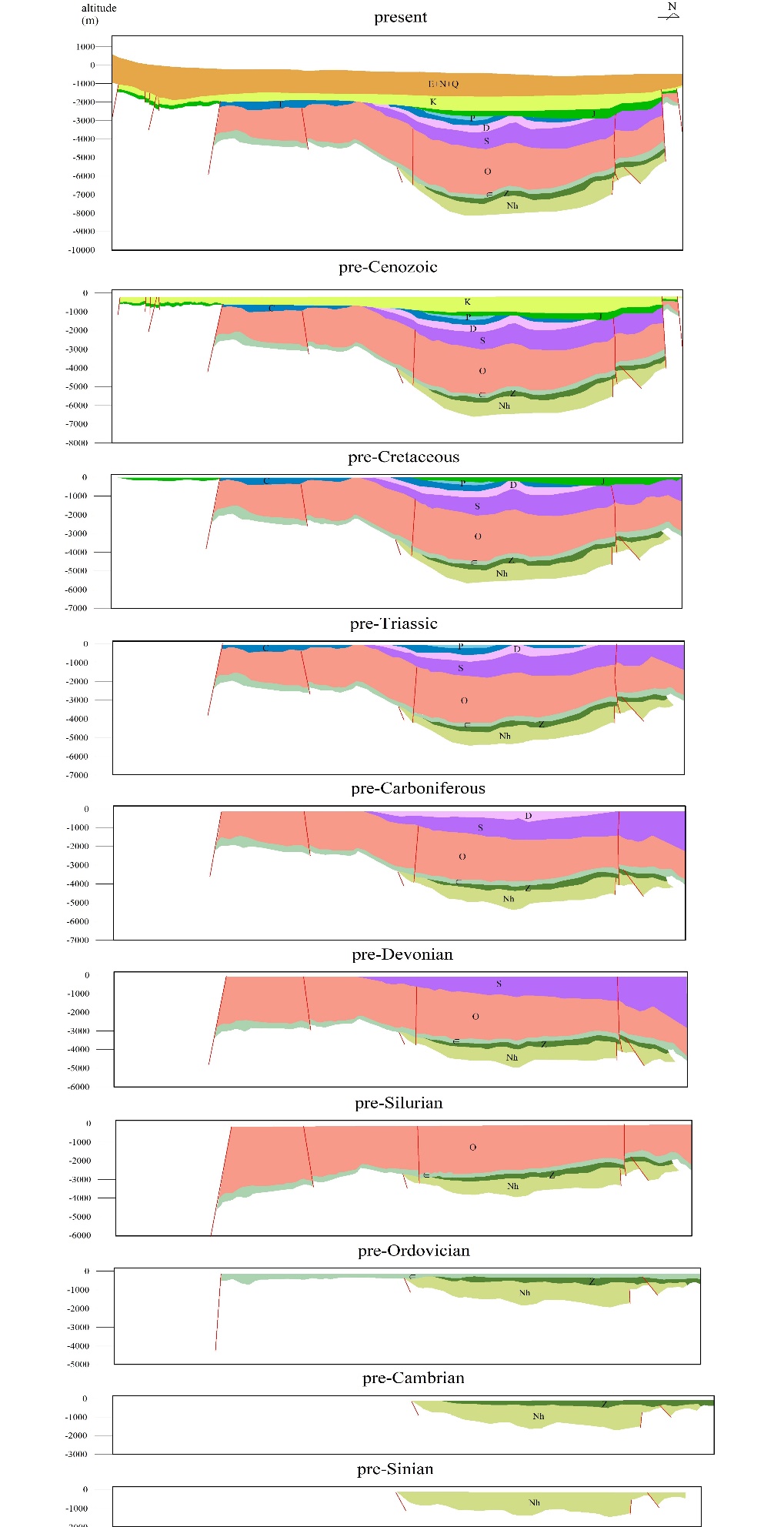


Fig. 4. Restored balanced geological transects of NS21

Since the shortening/extension amount was obtained (Table 1 and 2) directly in the process of restoration in 2D-MOVE, the uneven distribution of the data needs to be relocated to make an even one further. As the deformation mainly happened around the margin rather than inside the basin, this study attributed the shortening/extension amount to four evenly distributed measuring lines in Tarim Basin came up by Laborde et al. (2019), to display the shortening/extension amount reasonably. The shortening/extension amount of these four measuring lines were obtained by assigning more weight to its nearby data and less weight to its distant data to get a weighted average (Table 3).

Table 1. Shortening amount of Tarim Basin between the Cenozoic and Nanhua Period (/km)

| Measuring lines | Shortening amount of the southern margin/km | Shortening amount inside the basin/km | Shortening amount of the northern margin/km |
| --- | --- | --- | --- |
| NS05 | 19 | no data | no data |
| NS08 | 4.4 | 4.8 | no data |
| NS11 | 7.0 | 1.4 | 19.5 |
| NS13 | 5.1 | 15.8 | no data |
| EW03 | 5.1 | no data | 2.3 |
| NS16 | no data | 10.3 | 18.1 |
| NS19 | no data | 8.3 | 5.9 |
| NS08+EW09+NS20 | no data | 2.3 | no data |
| NS21 | 3.6 | 14.4 | no data |
| NS16+EW17+NS23 | 1.9 | 12.0 | 18.1 |

Table 2. Shortening amount of Tarim Basin between the Cenozoic and Sinian Period (/km)

| Measuring lines | Shortening amount of the southern margin/km | Shortening amount inside the basin/km | Shortening amount of the northern margin/km |
| --- | --- | --- | --- |
| NS05 | no data | no data | 20.4 |
| NS08 | 6.9 | 2.2 | 2.9 |
| NS11 | 8.3 | 1.8 | 2.6 |
| NS13 | 67.5 | no data | 9.3 |
| EW03 | 21.4 | no data | 5.2 |
| NS16 | 42.5 | no data | no data |
| NS19 | 15.5 | 26.8 | 4.1 |
| NS08+EW09+NS20 | 15.4 | no data | 2.9 |
| NS21 | 2.1 | 6.2 | 3 |
| NS16+EW17+NS23 | 2.3 | 19.5 | no data |

For example, the shortening amount of the southern margin of NS13 between the Cenozoic and Sinian Period was obviously abandoned, for the consultant of the 20 restored balanced geological transects (Fig. 1 for example) obtained from the Tarim Oilfield Company.

Table 3. Shortening amount of Tarim Basin between the Cenozoic, Sinian, Nanhua Periods and present (/km)

| Distribution of the shortening amount | Cenozoic (Laborde et al., 2019) | Sinian Period (This study) | Nanhua Period (This study) |
| --- | --- | --- | --- |
| Northern margin of ML1 | 36.0 | 46.0 | 42.0 |
| Southern margin of ML1 | 32.0 | 42.0 | 40.0 |
| Northern margin of ML2 | 21.0 | 43.0 | 39.0 |
| Southern margin of ML2 | 35.0 | 41.0 | 40.0 |
| Northern margin of ML3 | 22.0 | 36.0 | 33.0 |
| Southern margin of ML3 | 0.9 | 10.9 | 7.9 |
| Northern margin of ML4 | 0.0 | 17.0 | 9.0 |
| Southern margin of ML4 | 0.3 | 23.3 | 16.3 |

To make it easier to understand the shortening/extension amount of Tarim Basin, two figures (Fig. 5 and Fig. 6) were used to display. The red numbers were the amount from Cenozoic and the black ones were from the Nanhua and Sinian Periods.


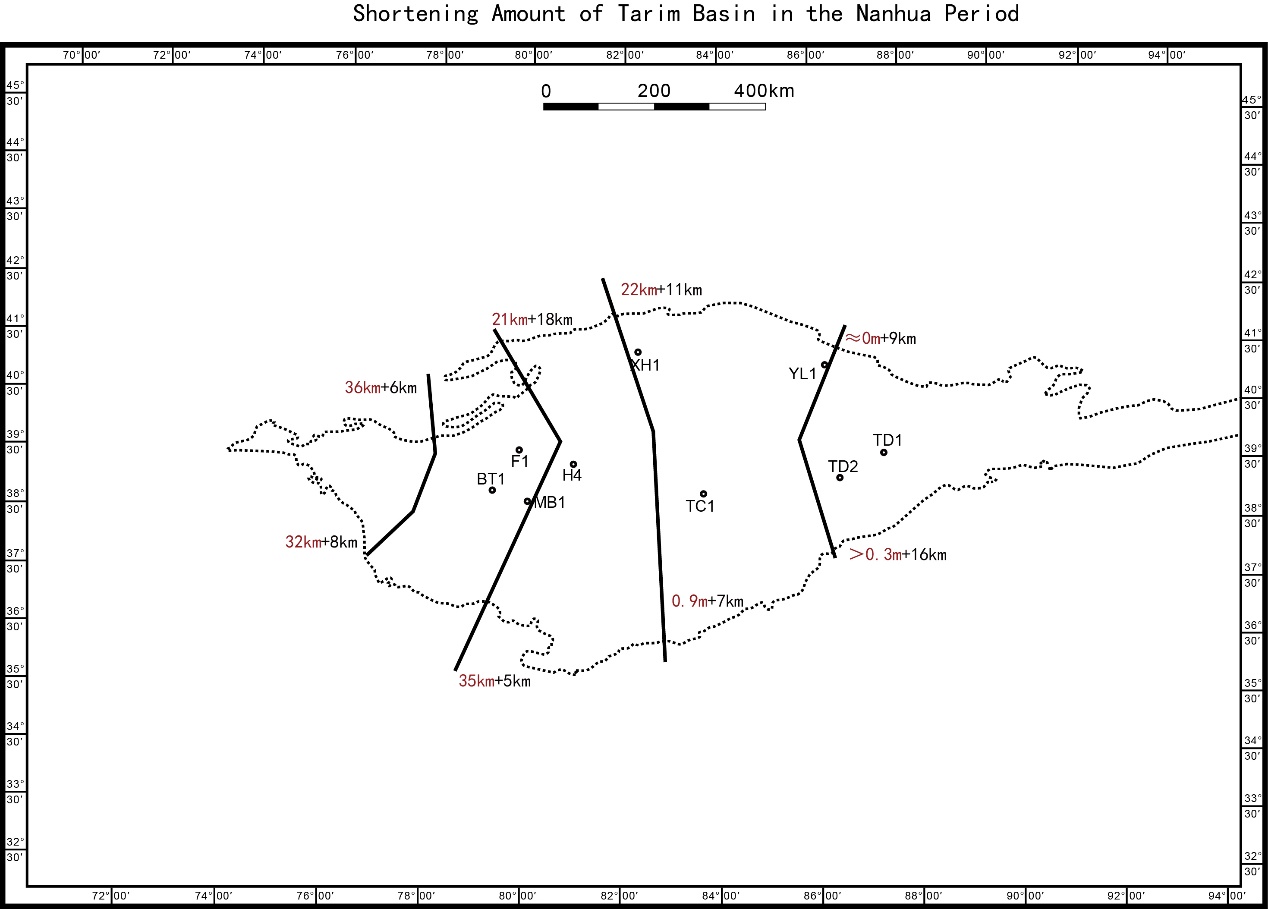


Fig. 5. Shortening amount of Tarim Basin in the Nanhua Period


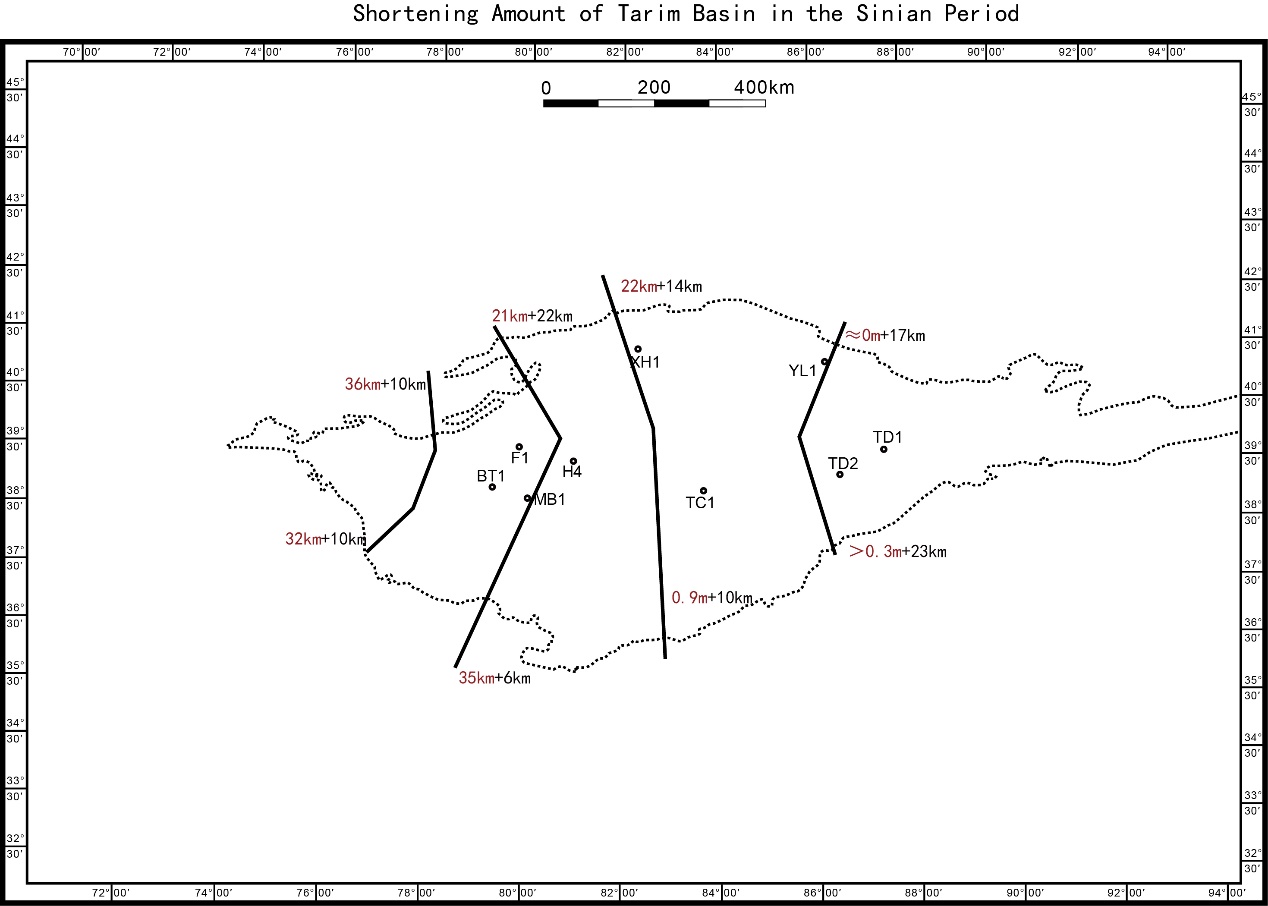


Fig. 6. Shortening amount of Tarim Basin in the Sinian Period
