# Supplementary figures and images for: Analysis of proto-type Tarim Basin in the late Precambrian and the dynamic mechanism of its evolution
Source: PLoS One. 2023 Jun 7;18(6):e0286849. doi: 10.1371/journal.pone.0286849 (PMC10246823; doi:10.1371/journal.pone.0286849)

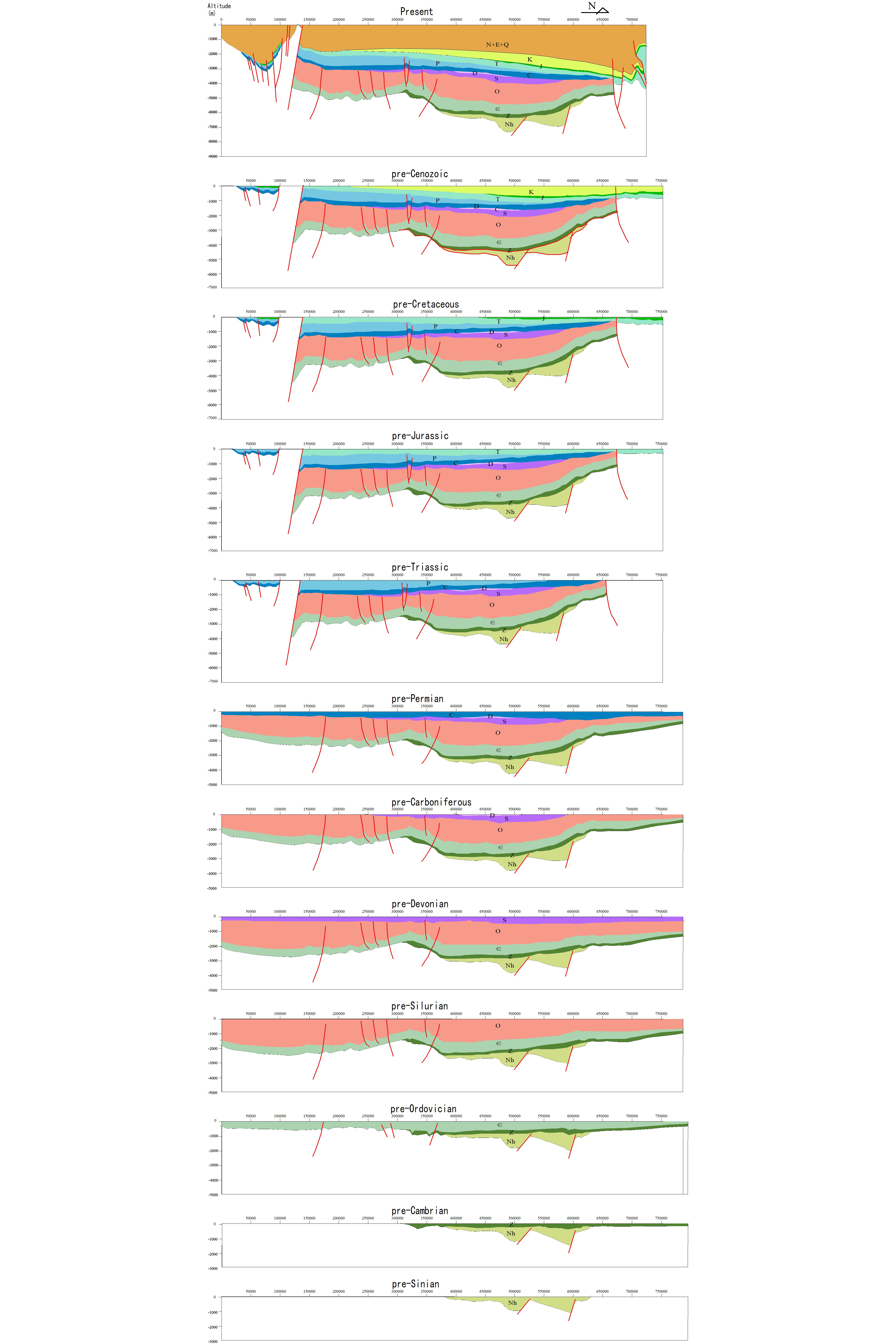

Supplement: S2 File — (ZIP) [file pone.0286849.s002.zip › 10 balanced geological transects this study restored/NS16.jpg]

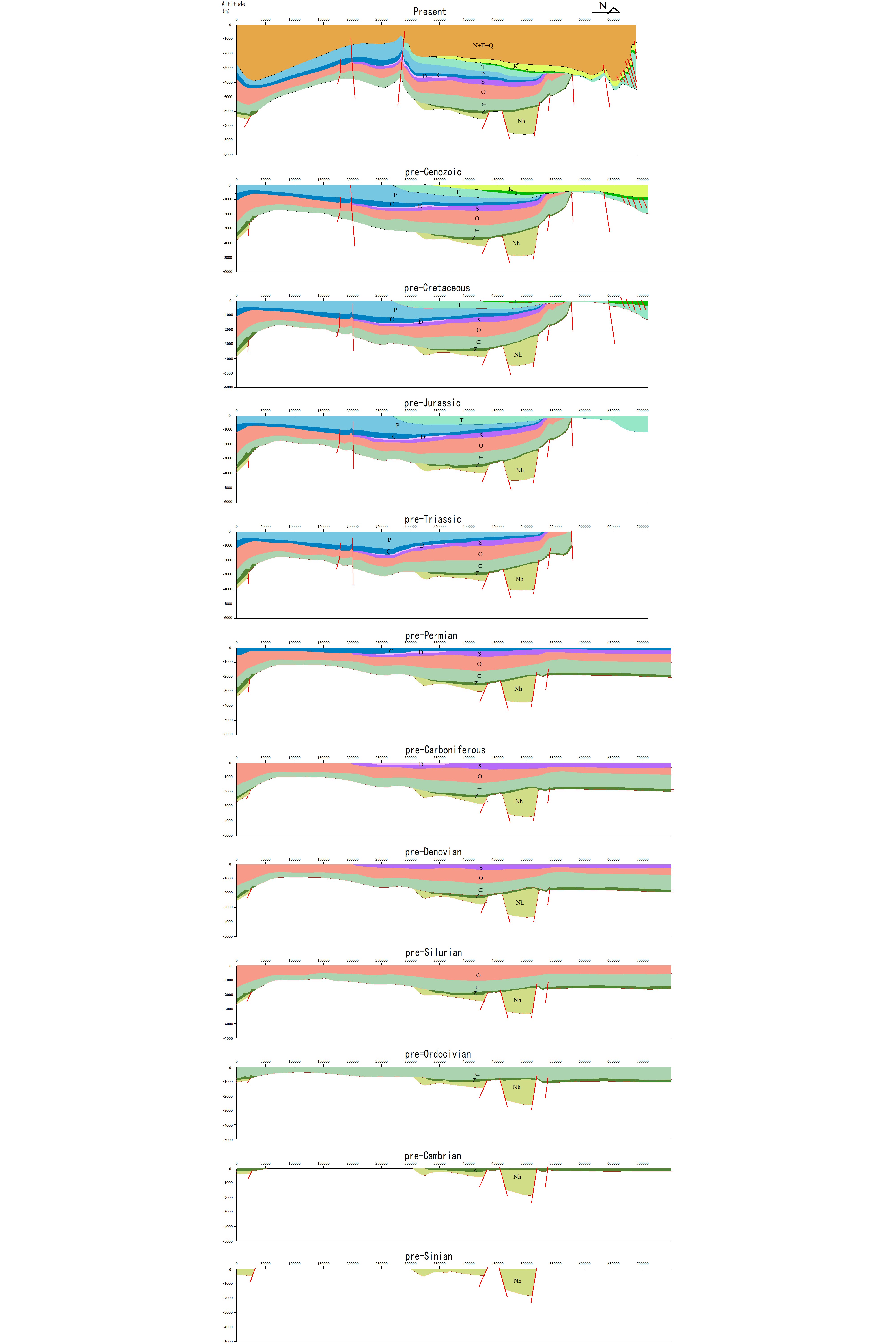

Supplement: S2 File — (ZIP) [file pone.0286849.s002.zip › 10 balanced geological transects this study restored/NS13.jpg]

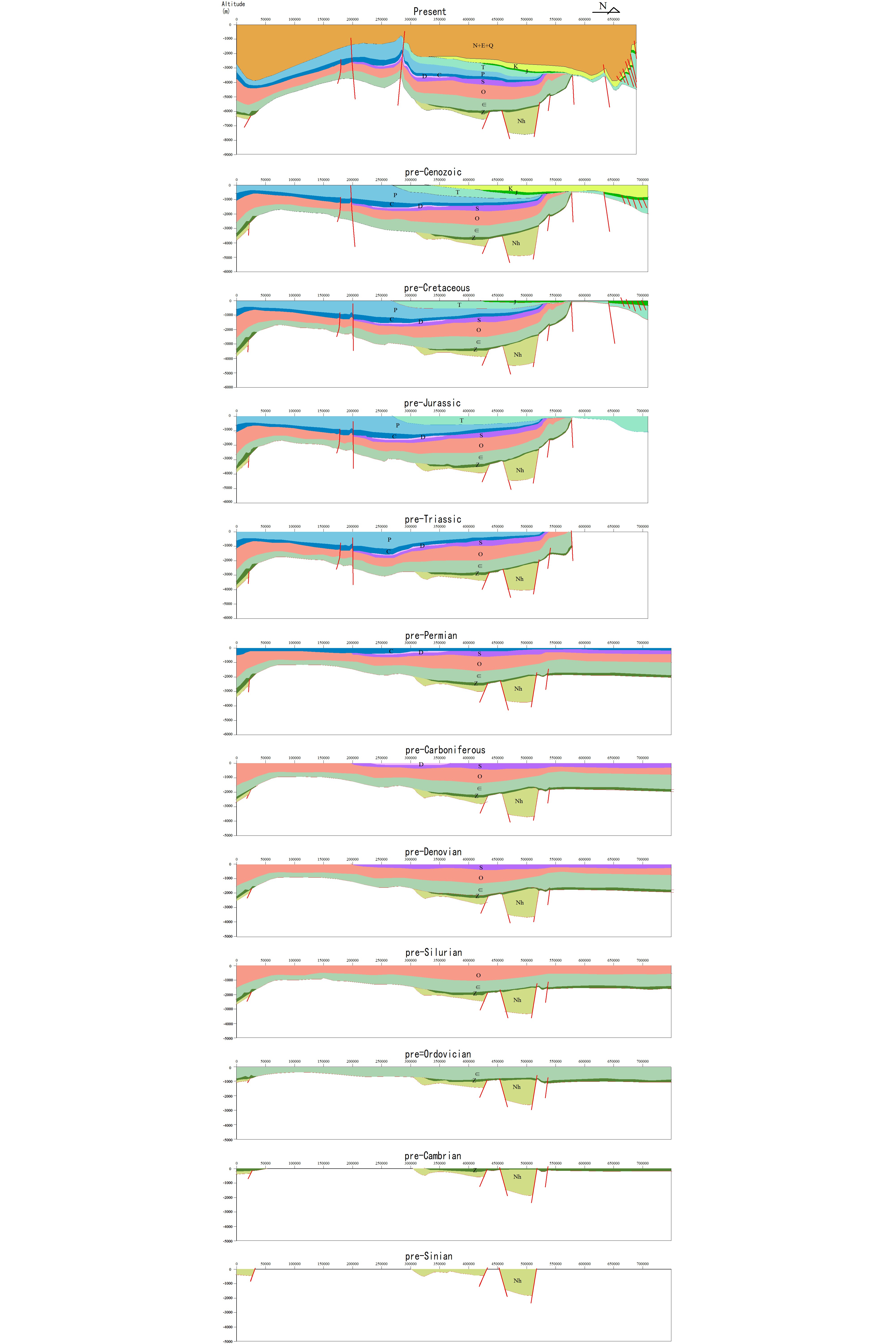

Supplement: S2 File — (ZIP) [file pone.0286849.s002.zip › 10 balanced geological transects this study restored/NS05.jpg]

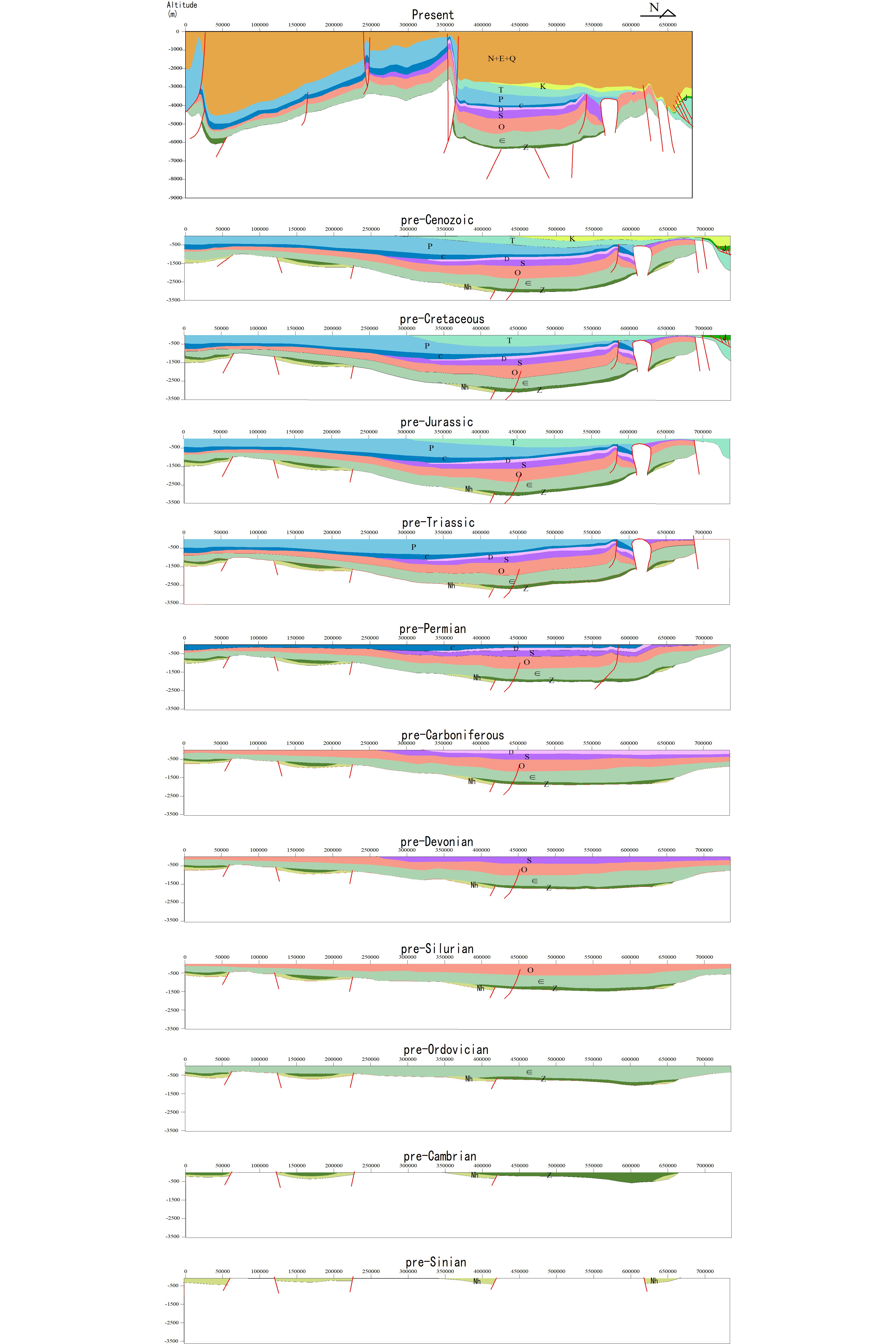

Supplement: S2 File — (ZIP) [file pone.0286849.s002.zip › 10 balanced geological transects this study restored/NS11.jpg]

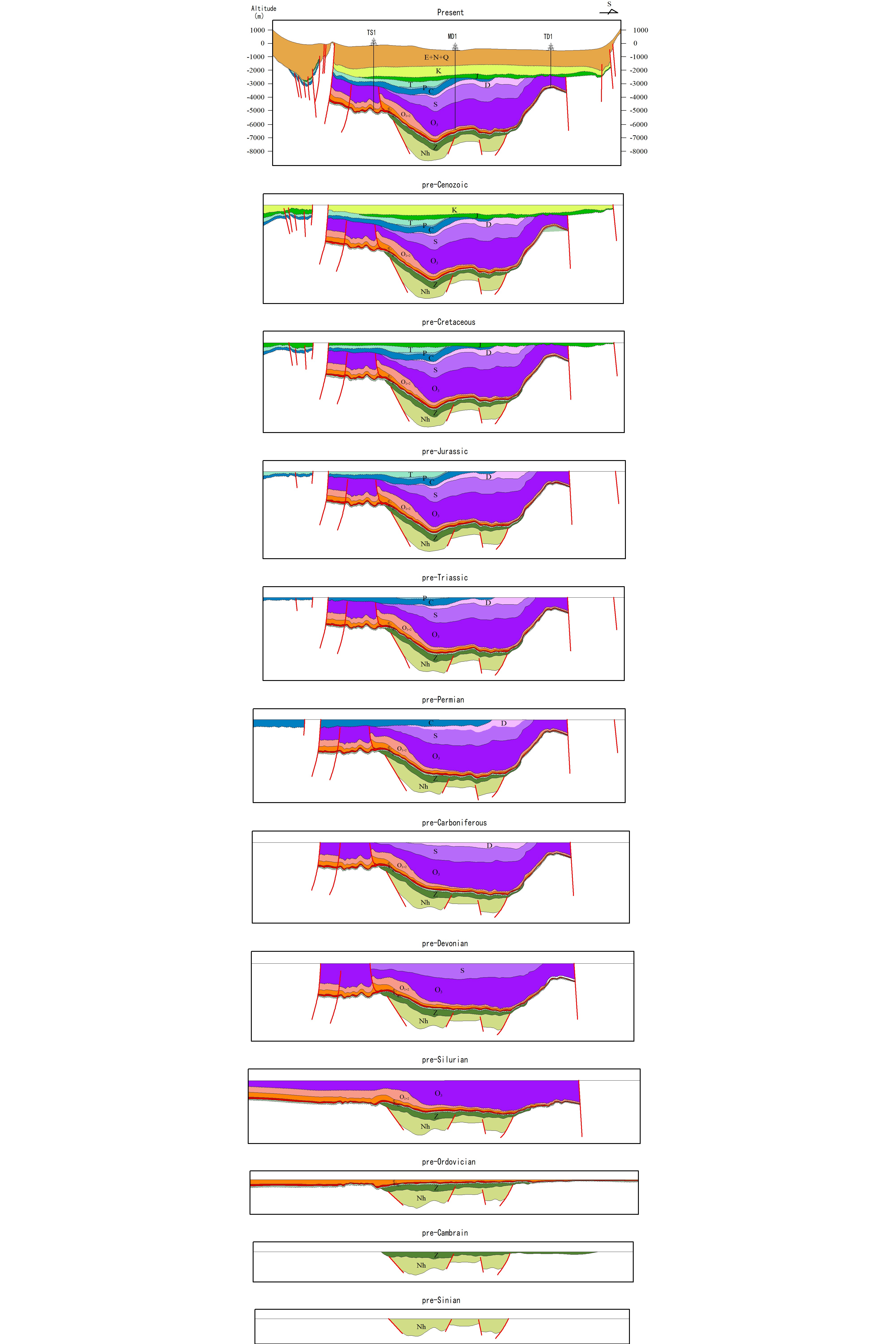

Supplement: S2 File — (ZIP) [file pone.0286849.s002.zip › 10 balanced geological transects this study restored/NS16+EW17+NS23.jpg]

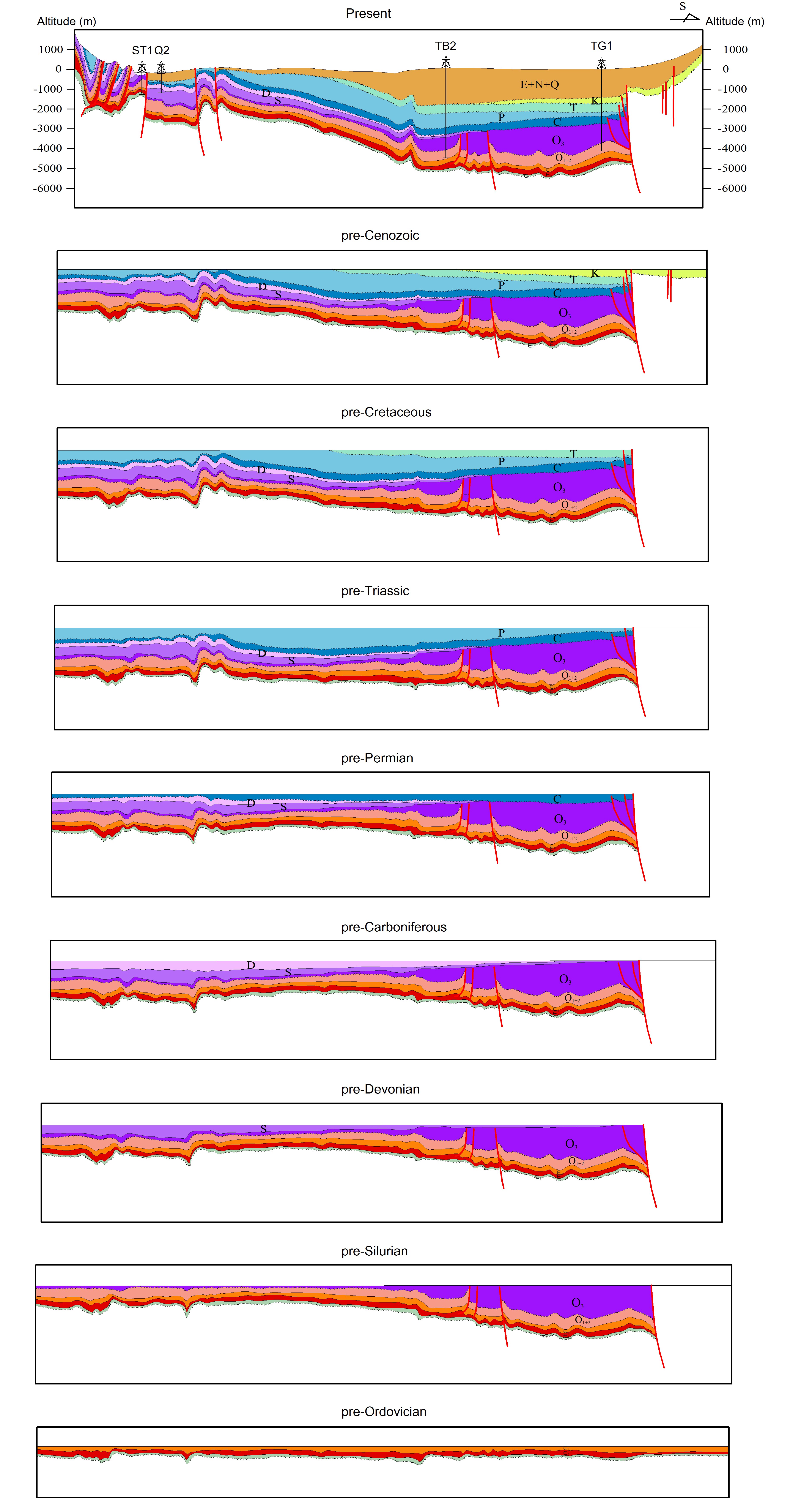

Supplement: S2 File — (ZIP) [file pone.0286849.s002.zip › 10 balanced geological transects this study restored/NS08+EW09+NS20.jpg]

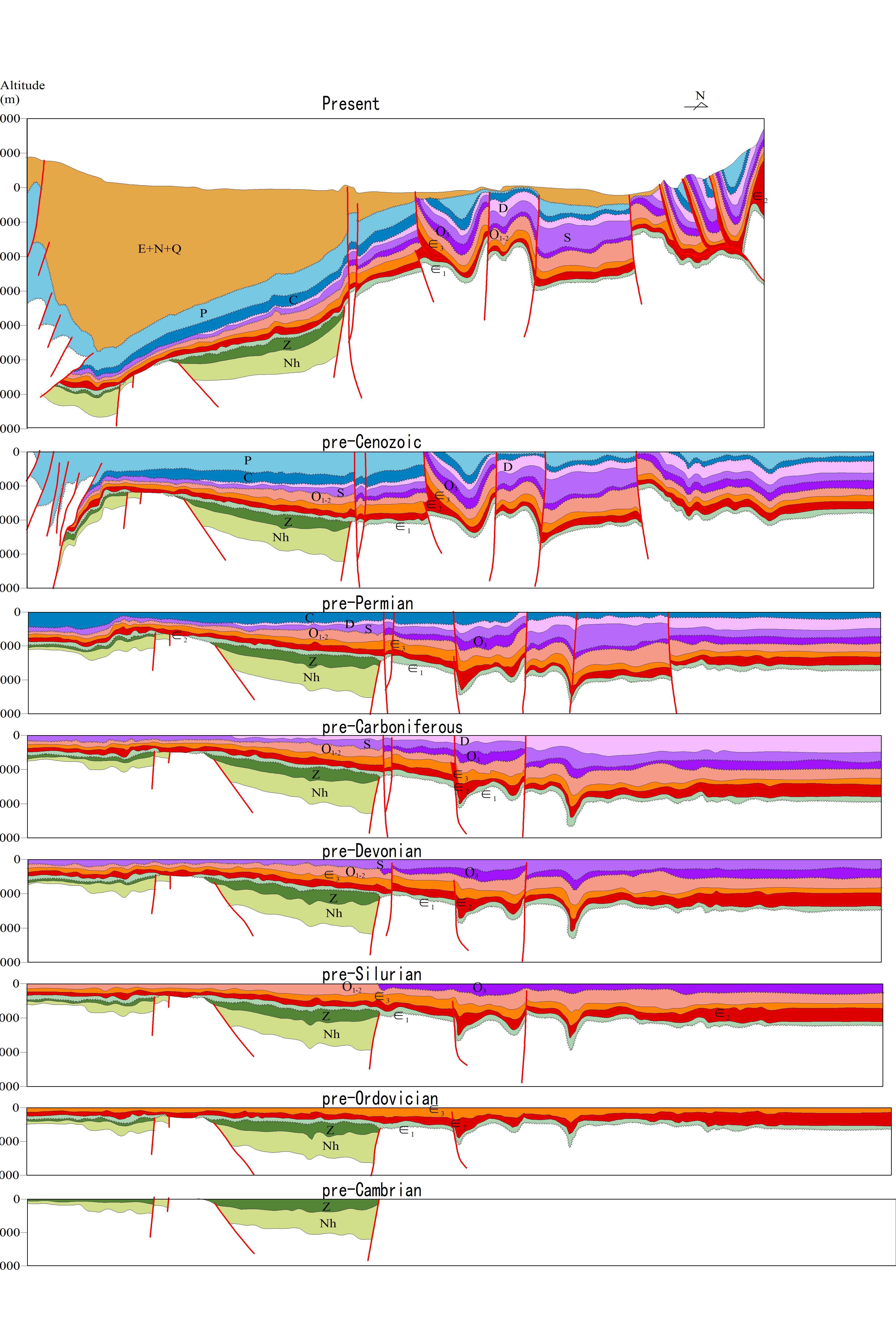

Supplement: S2 File — (ZIP) [file pone.0286849.s002.zip › 10 balanced geological transects this study restored/NS08.jpg]

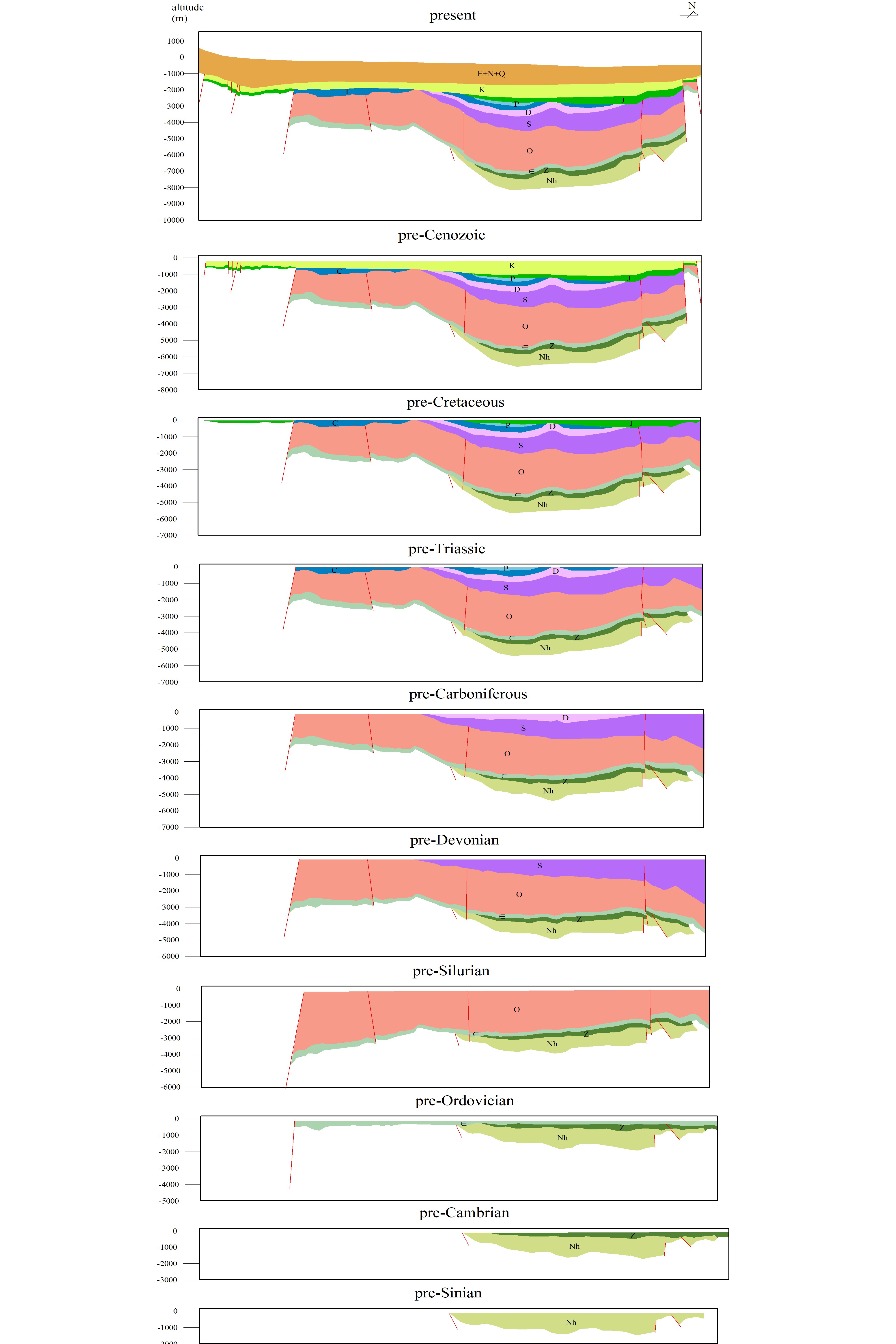

Supplement: S2 File — (ZIP) [file pone.0286849.s002.zip › 10 balanced geological transects this study restored/NS21.jpg]

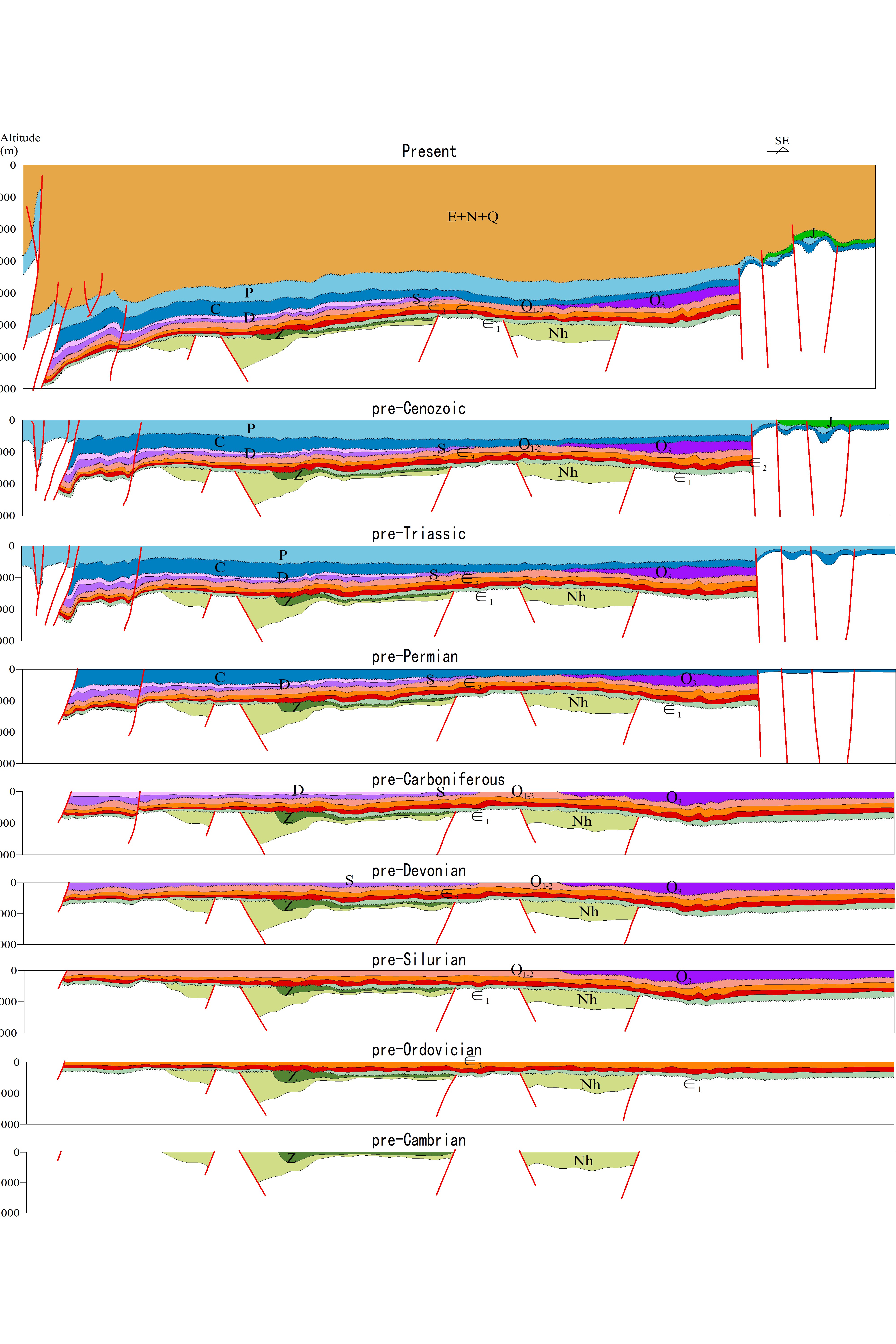

Supplement: S2 File — (ZIP) [file pone.0286849.s002.zip › 10 balanced geological transects this study restored/EW03.jpg]

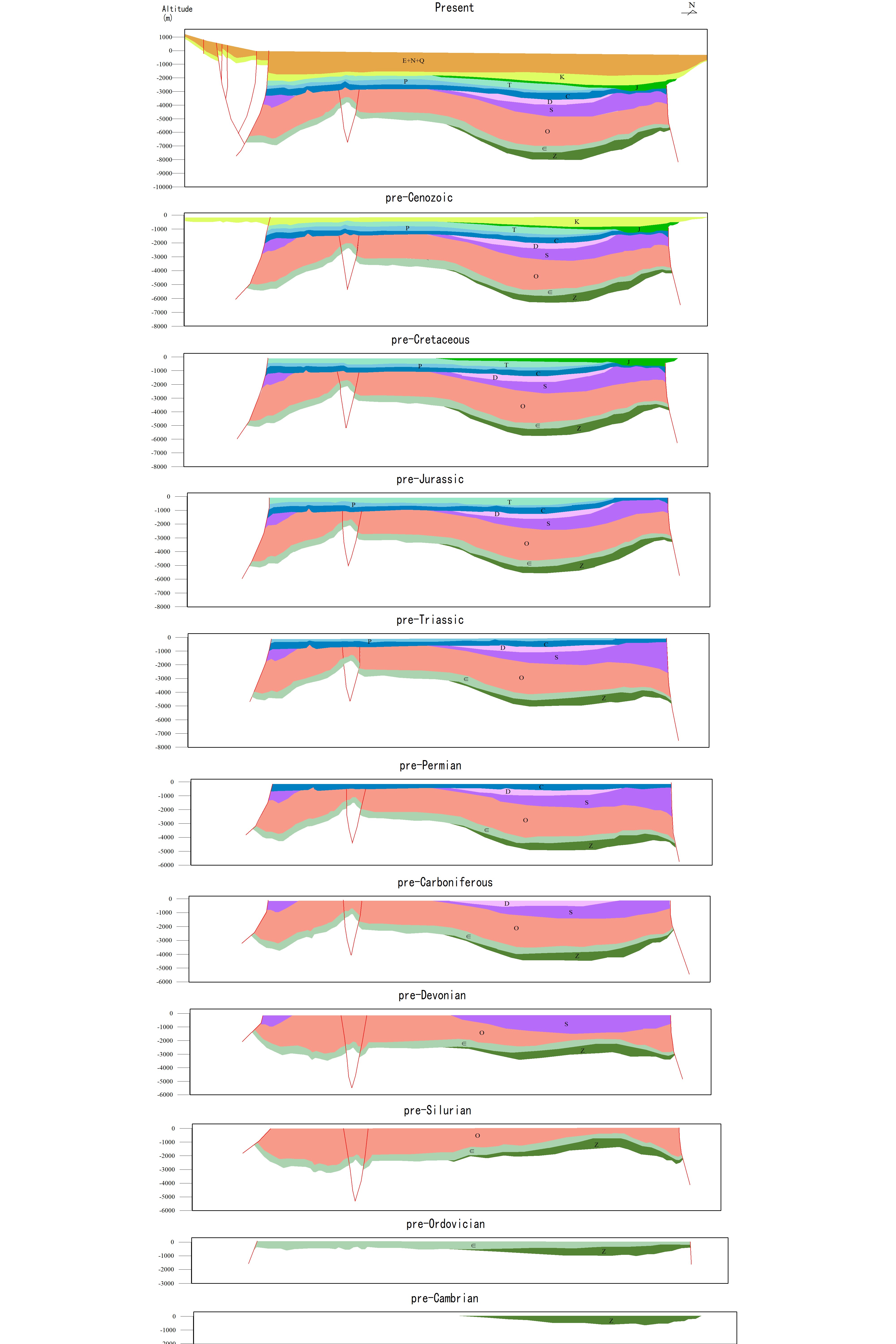

Supplement: S2 File — (ZIP) [file pone.0286849.s002.zip › 10 balanced geological transects this study restored/NS19.jpg]

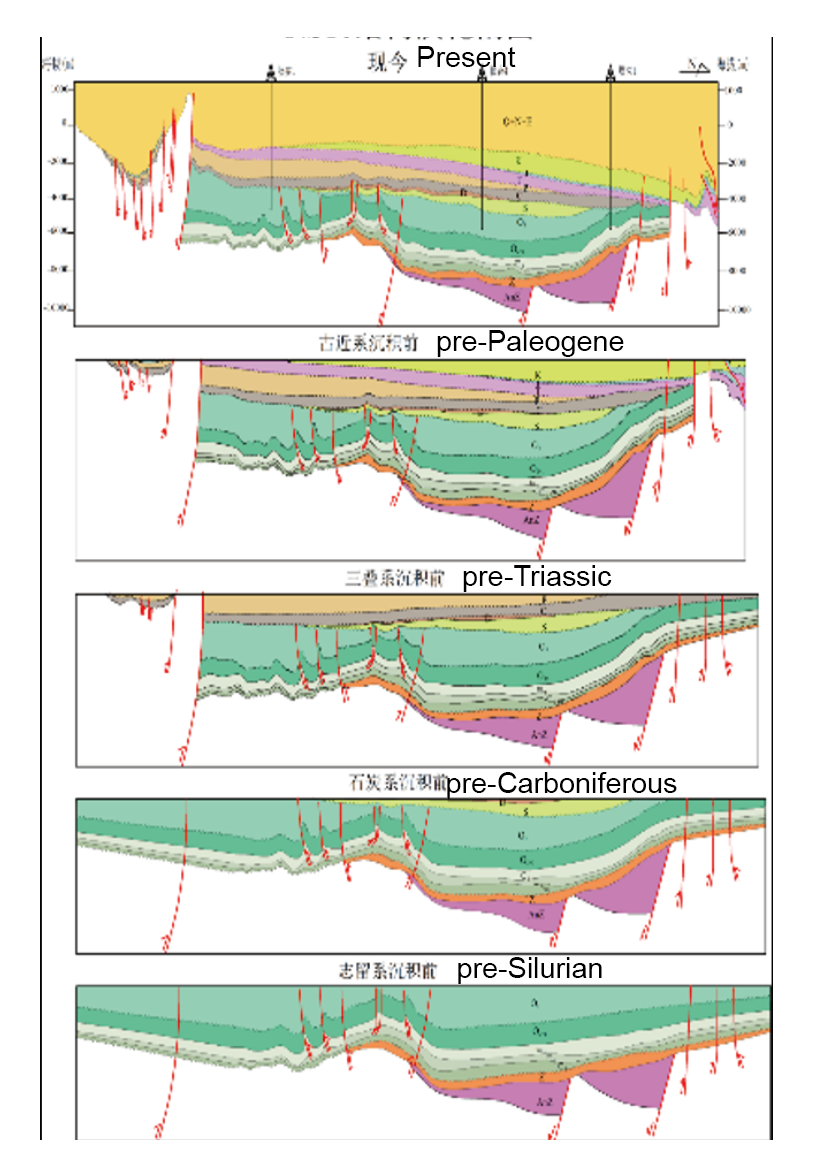

Supplement: S3 File — (ZIP) [file pone.0286849.s003.zip › 20 balanced geological transects from Tarim Oilfield Company/8.png]

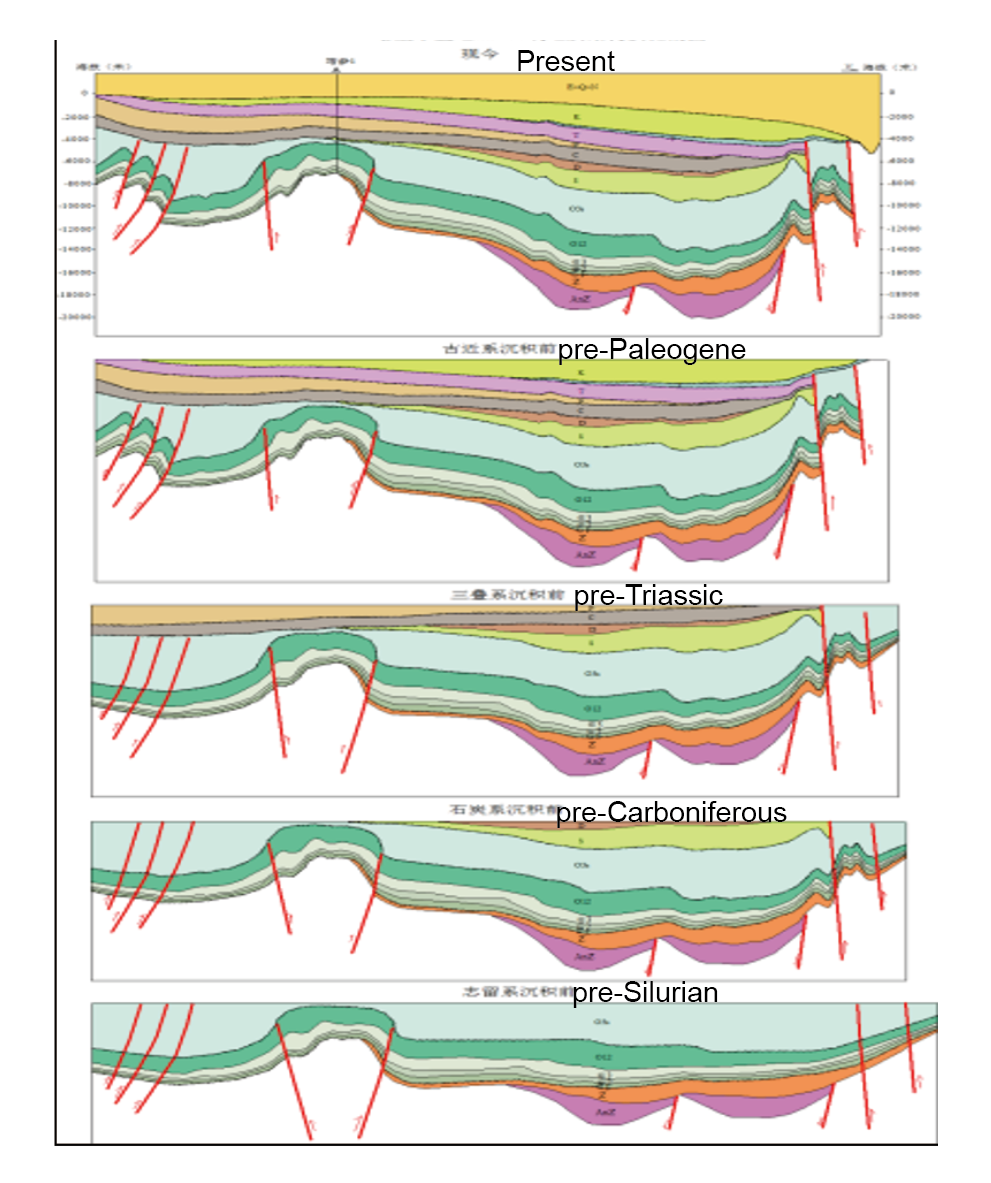

Supplement: S3 File — (ZIP) [file pone.0286849.s003.zip › 20 balanced geological transects from Tarim Oilfield Company/9.png]

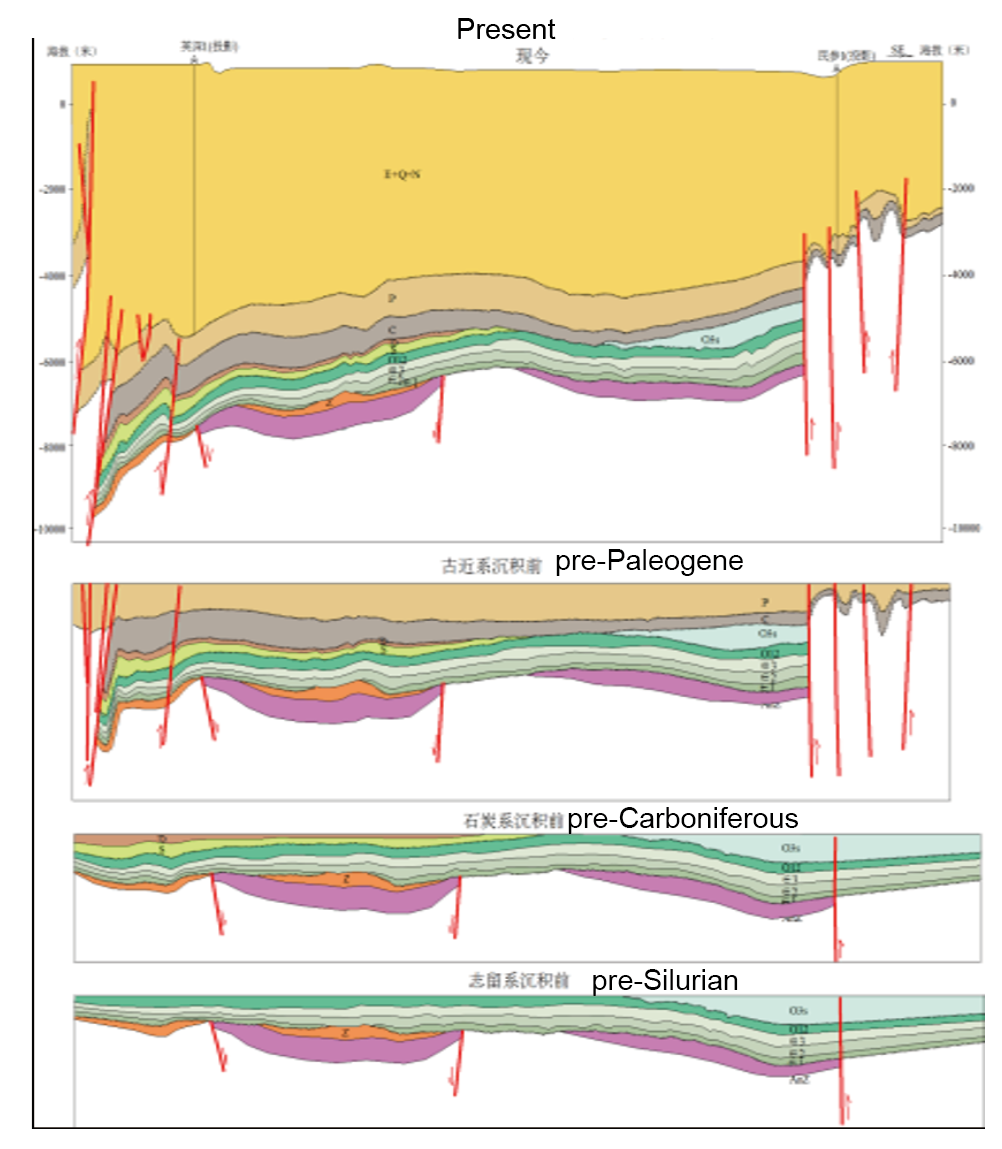

Supplement: S3 File — (ZIP) [file pone.0286849.s003.zip › 20 balanced geological transects from Tarim Oilfield Company/14.png]

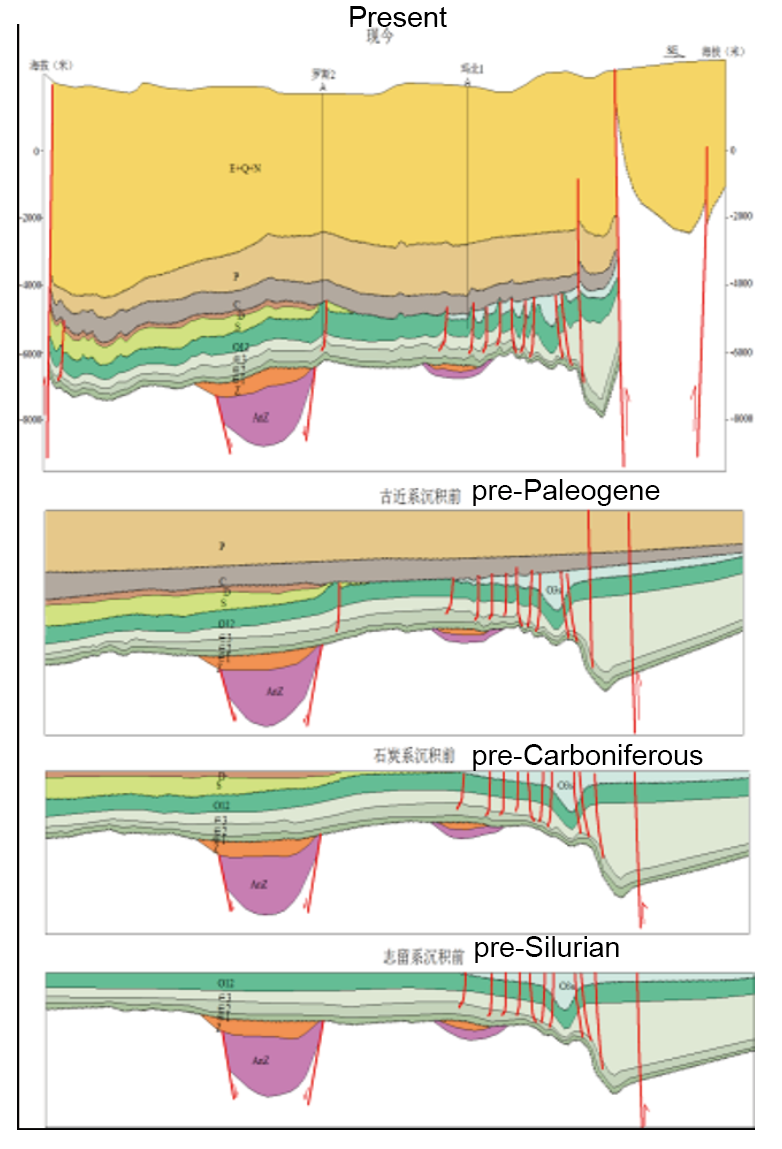

Supplement: S3 File — (ZIP) [file pone.0286849.s003.zip › 20 balanced geological transects from Tarim Oilfield Company/15.png]

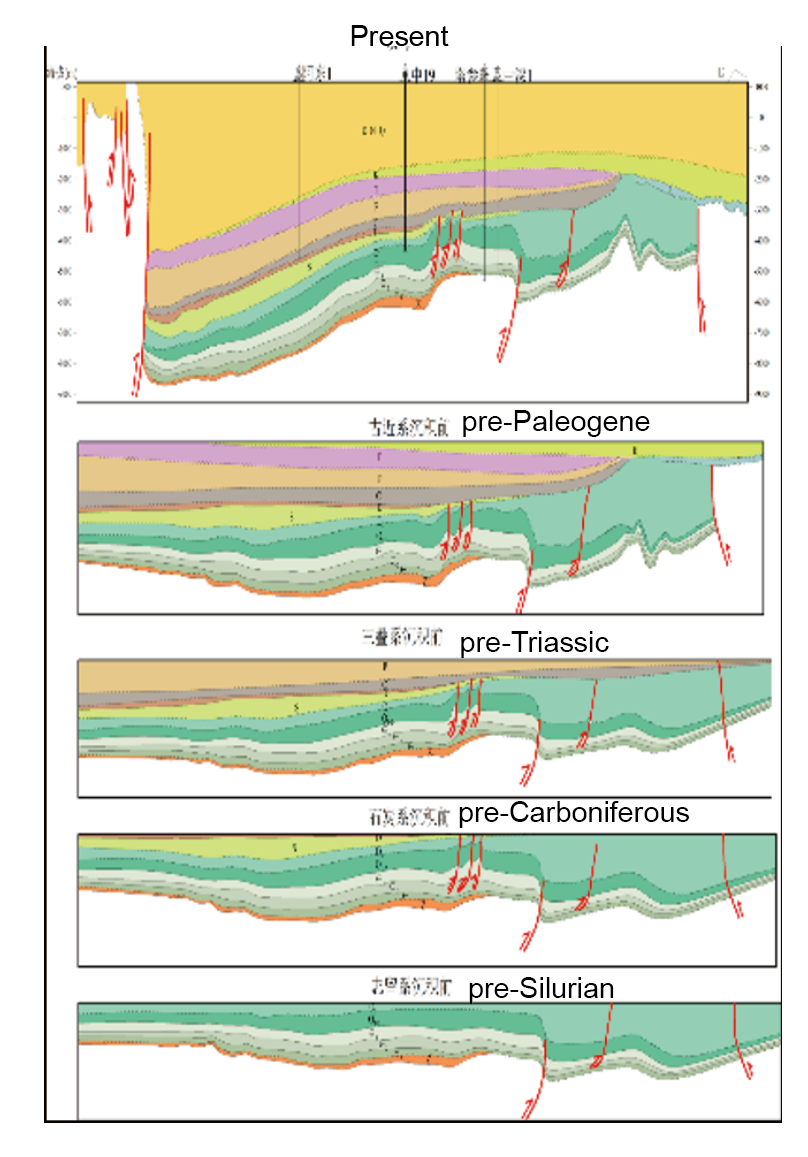

Supplement: S3 File — (ZIP) [file pone.0286849.s003.zip › 20 balanced geological transects from Tarim Oilfield Company/17.png]

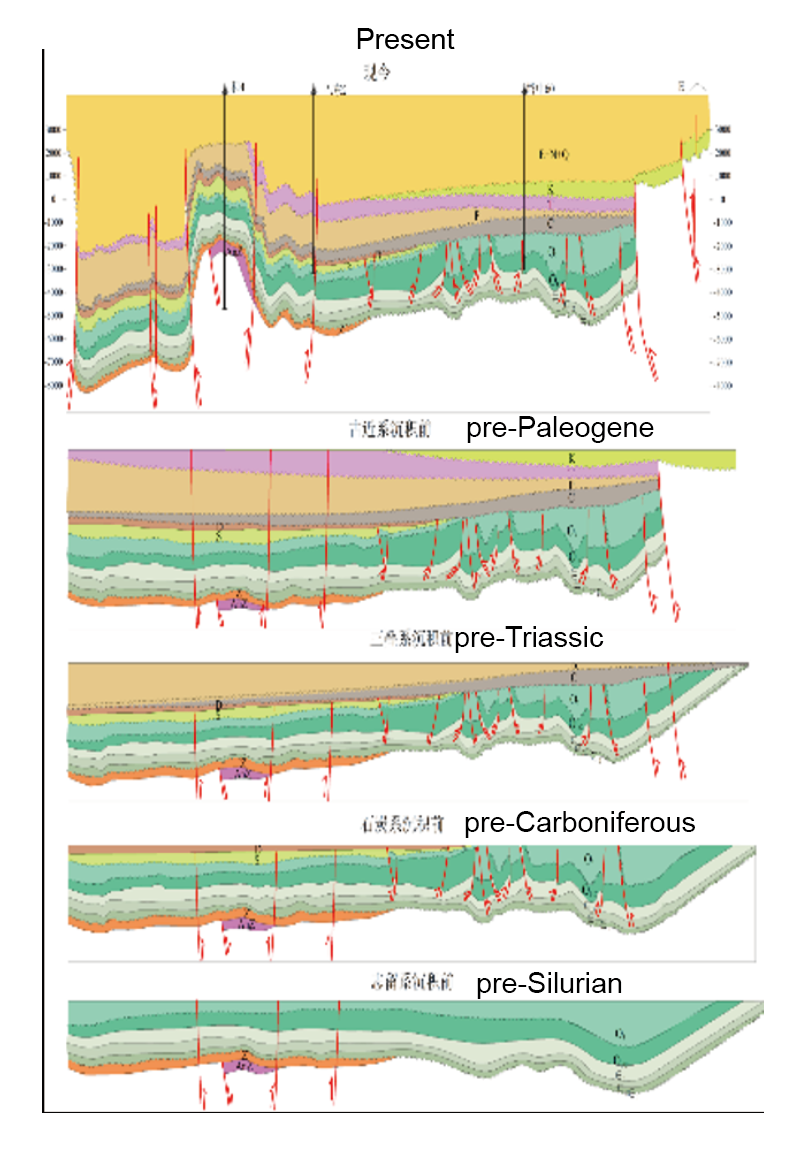

Supplement: S3 File — (ZIP) [file pone.0286849.s003.zip › 20 balanced geological transects from Tarim Oilfield Company/16.png]

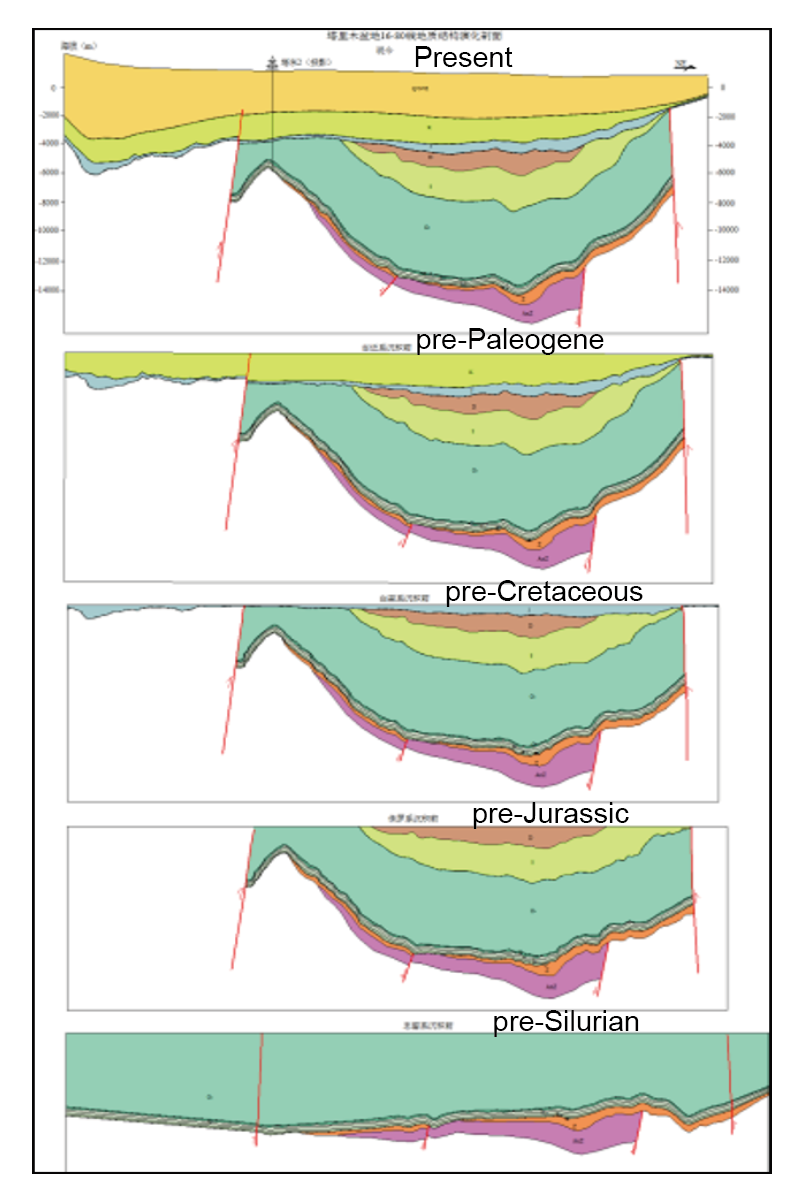

Supplement: S3 File — (ZIP) [file pone.0286849.s003.zip › 20 balanced geological transects from Tarim Oilfield Company/12.png]

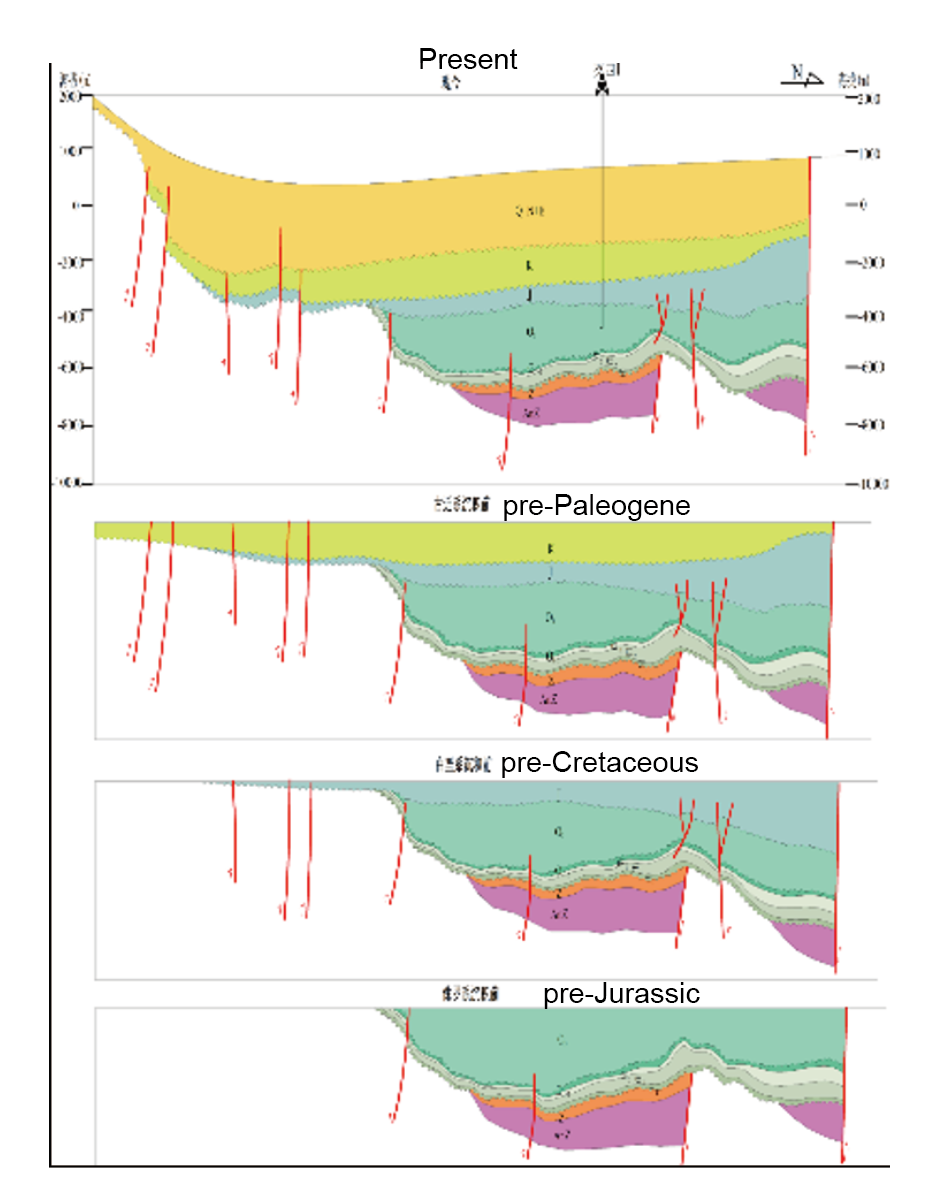

Supplement: S3 File — (ZIP) [file pone.0286849.s003.zip › 20 balanced geological transects from Tarim Oilfield Company/13.png]

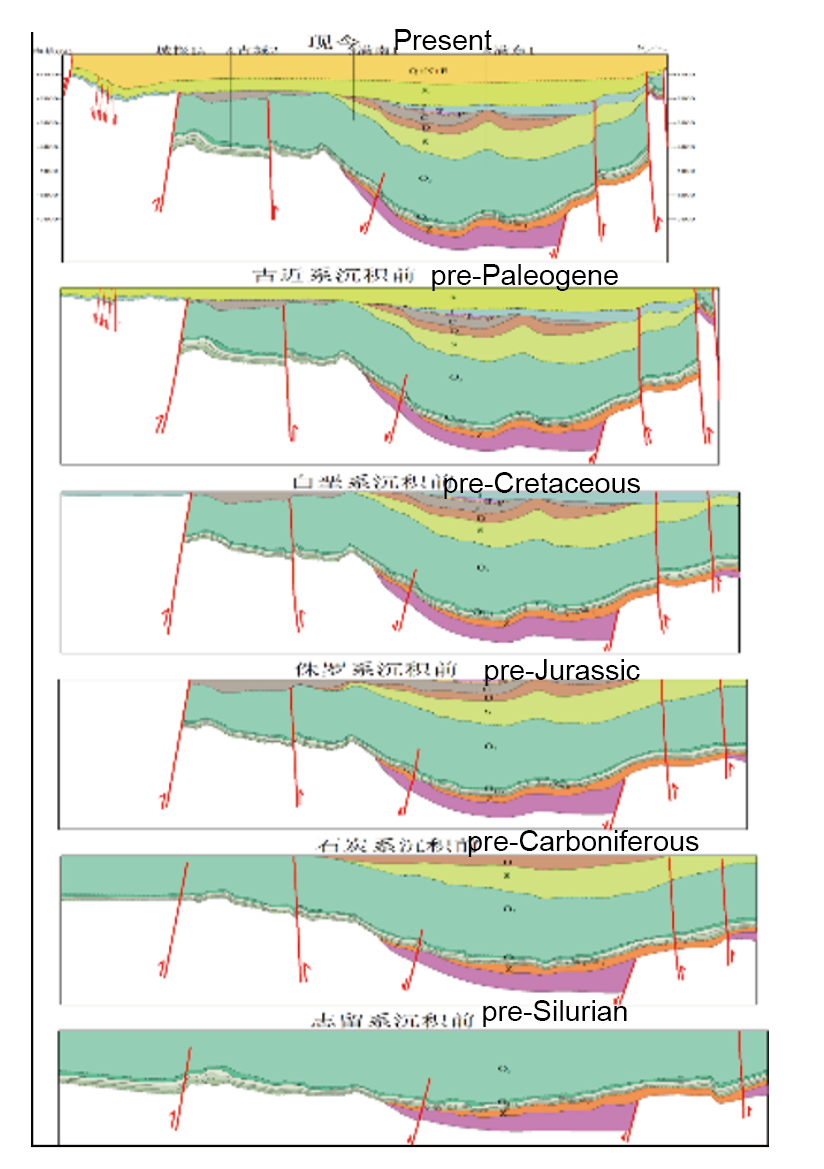

Supplement: S3 File — (ZIP) [file pone.0286849.s003.zip › 20 balanced geological transects from Tarim Oilfield Company/11.png]

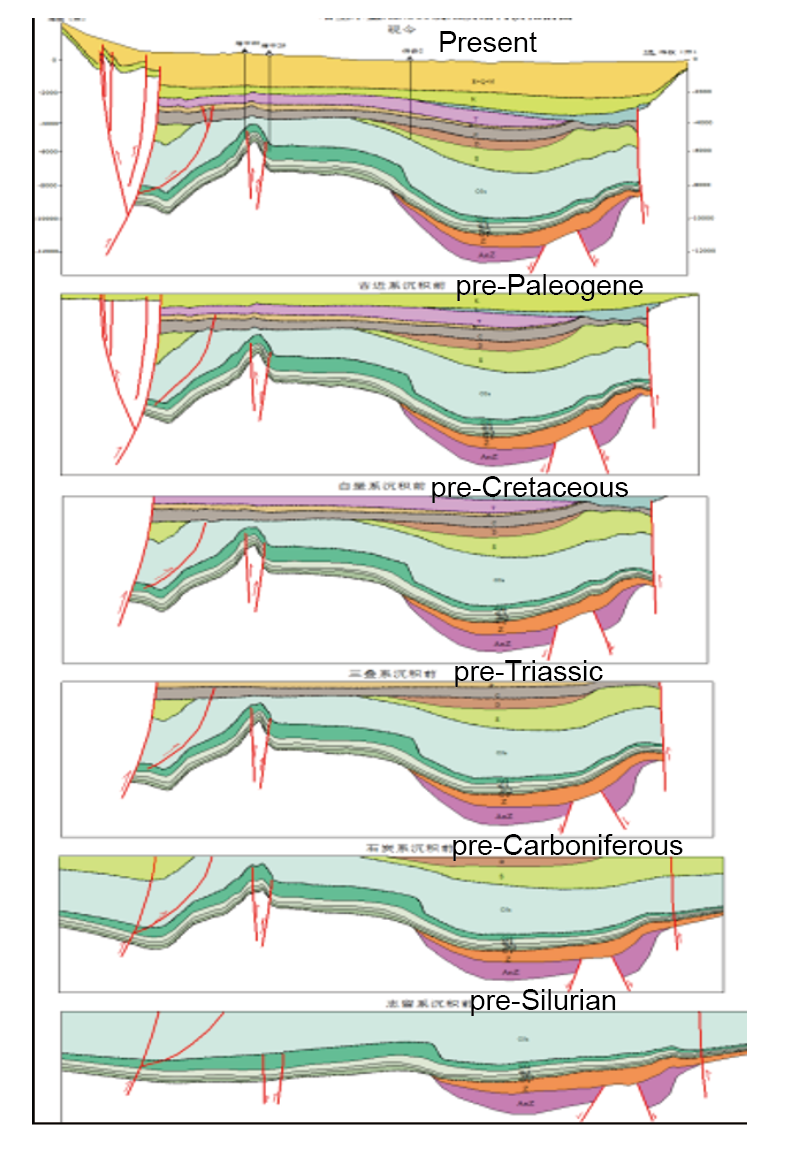

Supplement: S3 File — (ZIP) [file pone.0286849.s003.zip › 20 balanced geological transects from Tarim Oilfield Company/10.png]

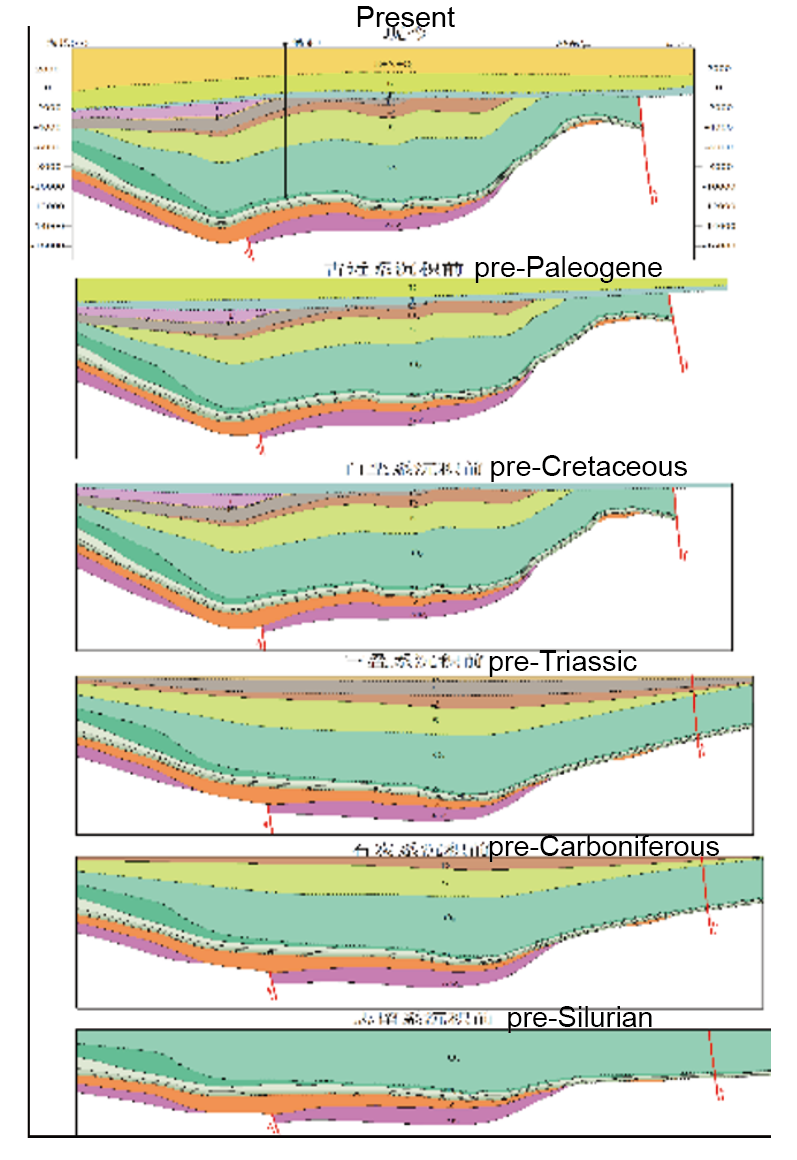

Supplement: S3 File — (ZIP) [file pone.0286849.s003.zip › 20 balanced geological transects from Tarim Oilfield Company/20.png]

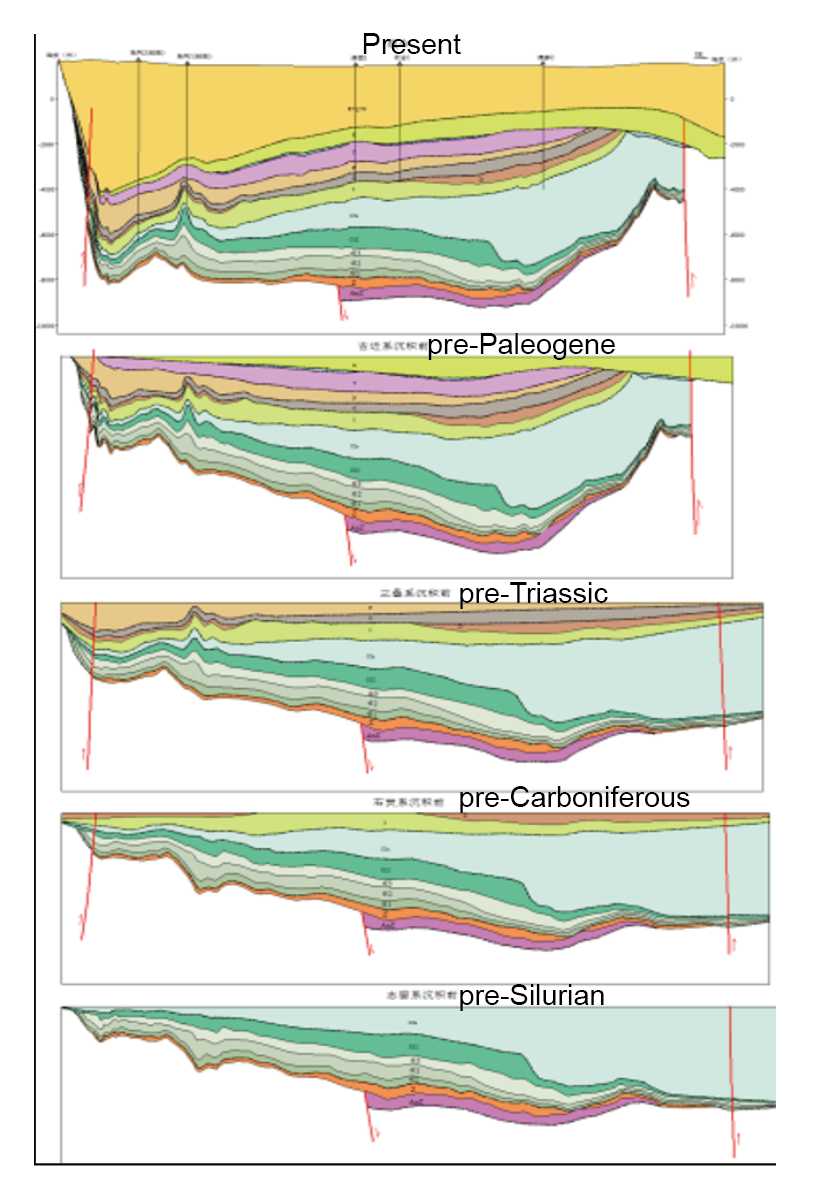

Supplement: S3 File — (ZIP) [file pone.0286849.s003.zip › 20 balanced geological transects from Tarim Oilfield Company/18.png]

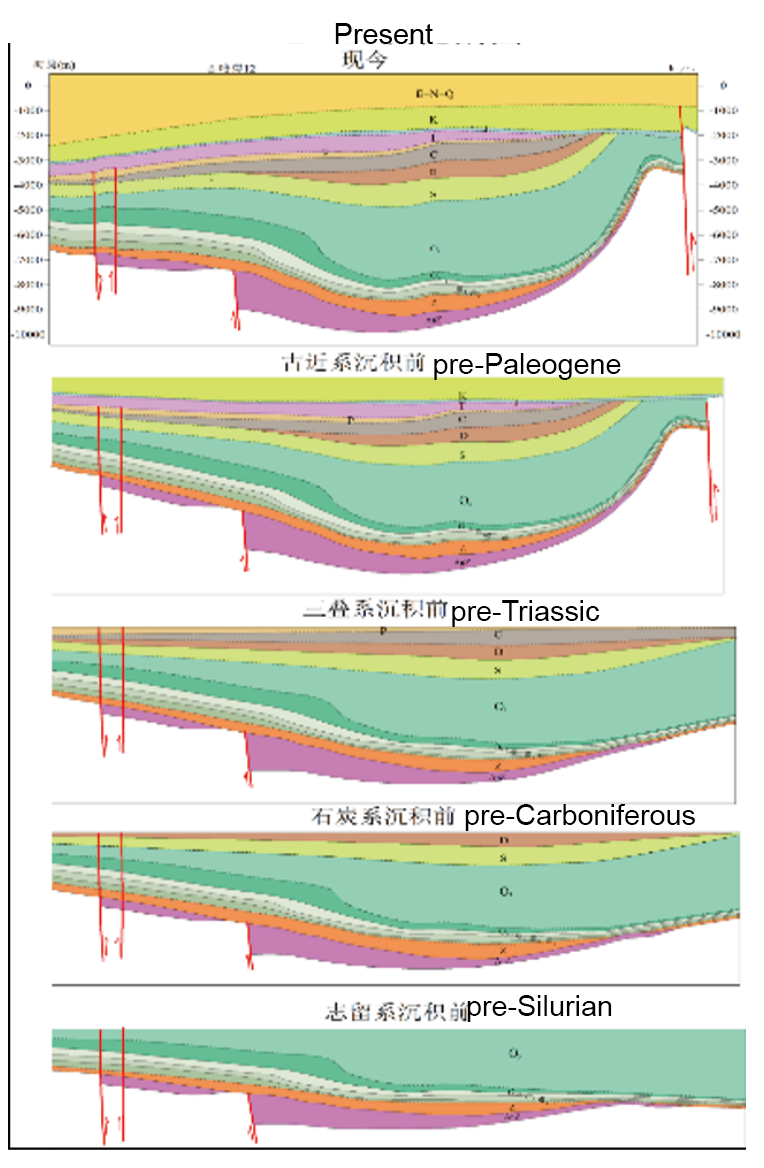

Supplement: S3 File — (ZIP) [file pone.0286849.s003.zip › 20 balanced geological transects from Tarim Oilfield Company/19.png]

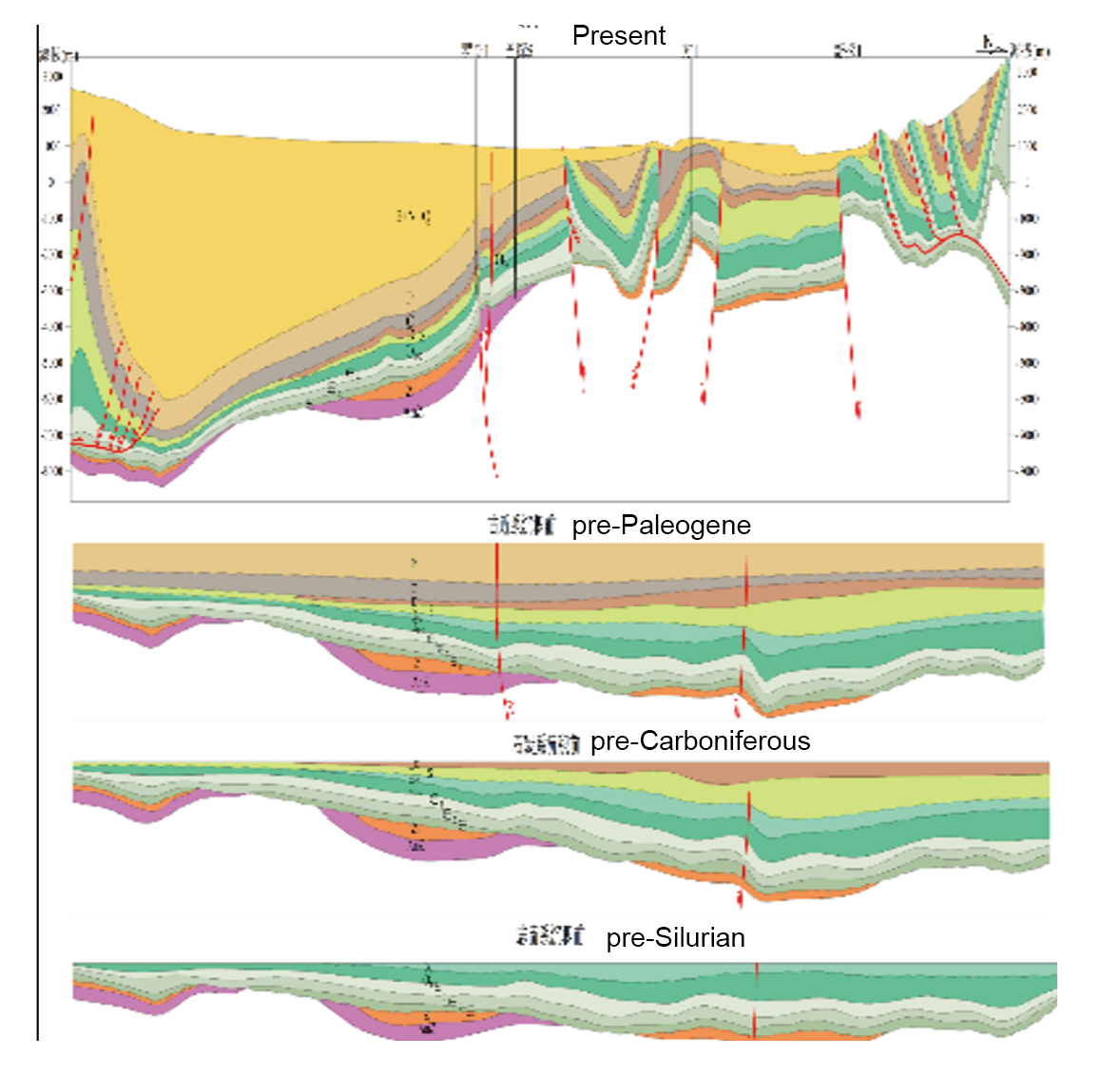

Supplement: S3 File — (ZIP) [file pone.0286849.s003.zip › 20 balanced geological transects from Tarim Oilfield Company/4.png]

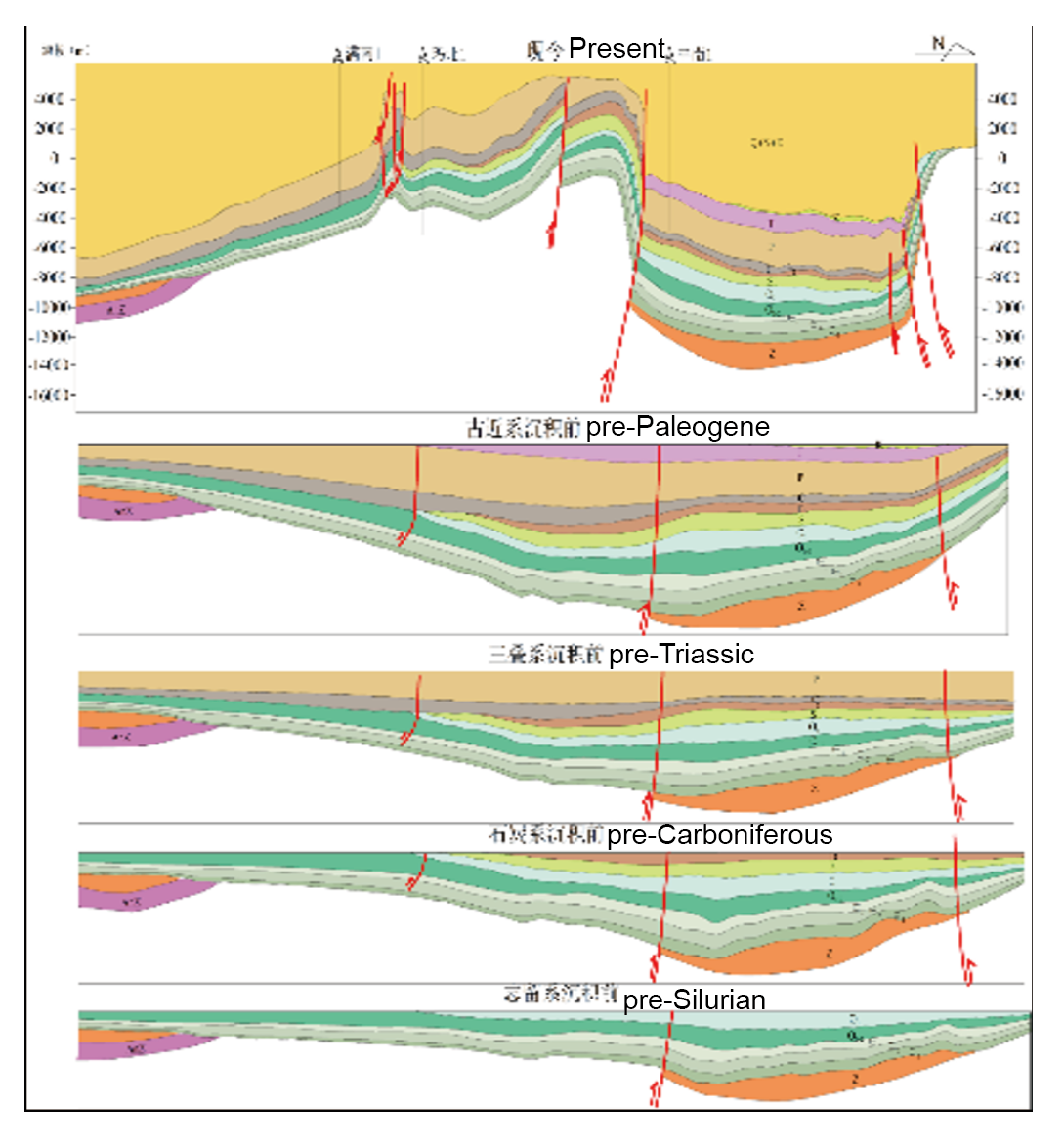

Supplement: S3 File — (ZIP) [file pone.0286849.s003.zip › 20 balanced geological transects from Tarim Oilfield Company/5.png]

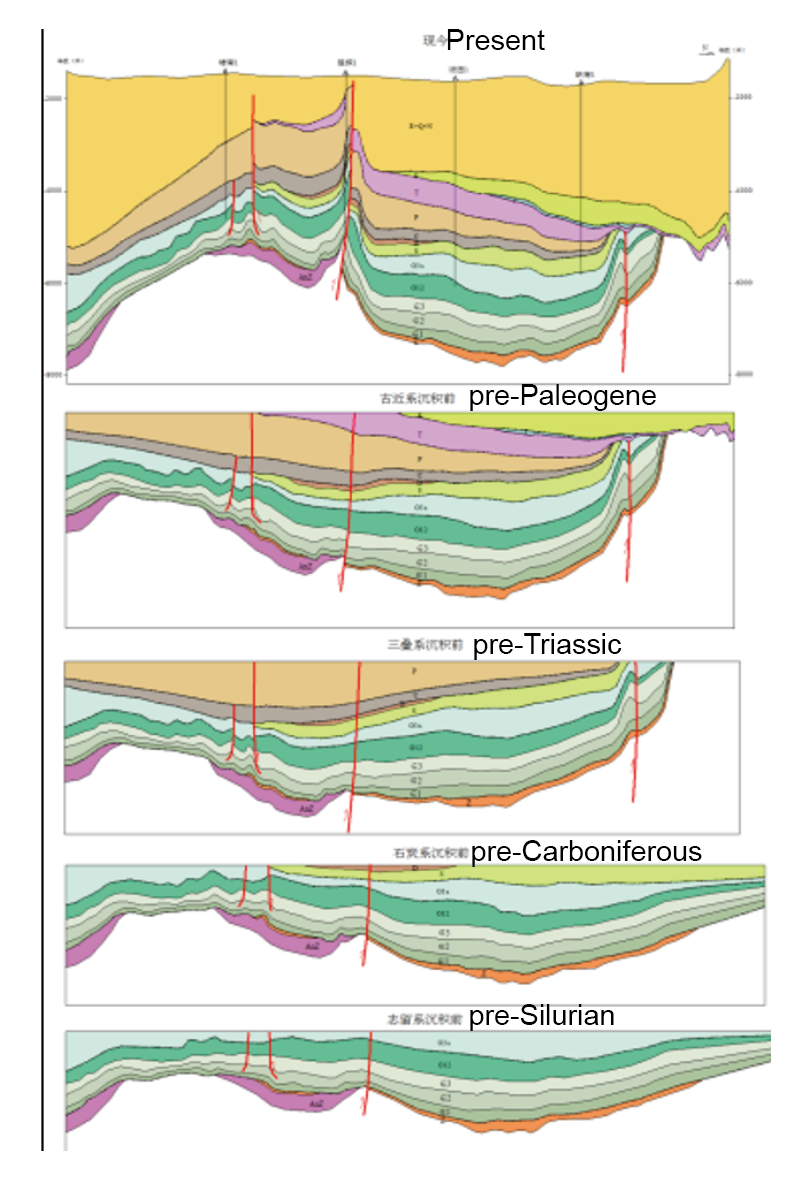

Supplement: S3 File — (ZIP) [file pone.0286849.s003.zip › 20 balanced geological transects from Tarim Oilfield Company/7.png]

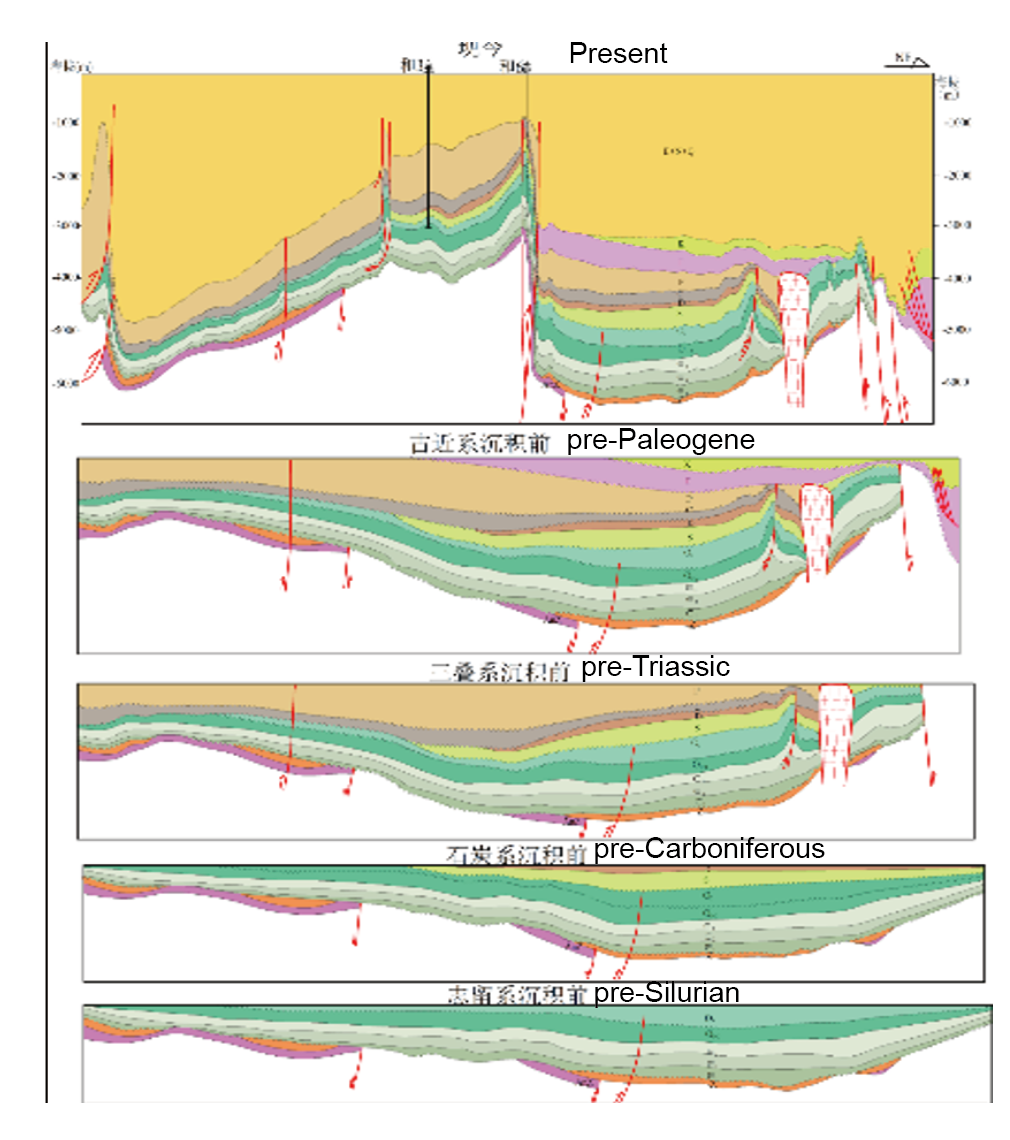

Supplement: S3 File — (ZIP) [file pone.0286849.s003.zip › 20 balanced geological transects from Tarim Oilfield Company/6.png]

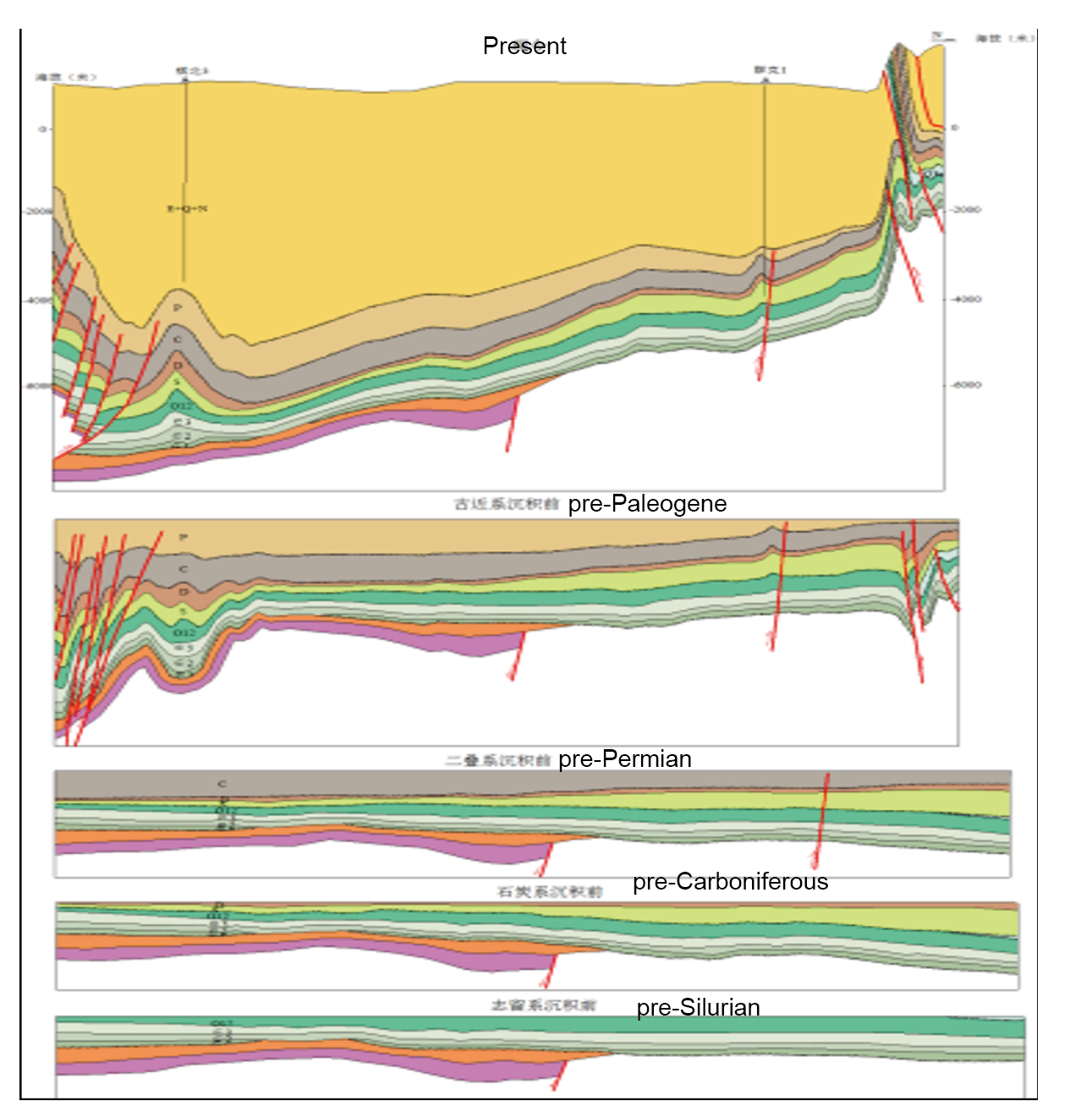

Supplement: S3 File — (ZIP) [file pone.0286849.s003.zip › 20 balanced geological transects from Tarim Oilfield Company/2.png]

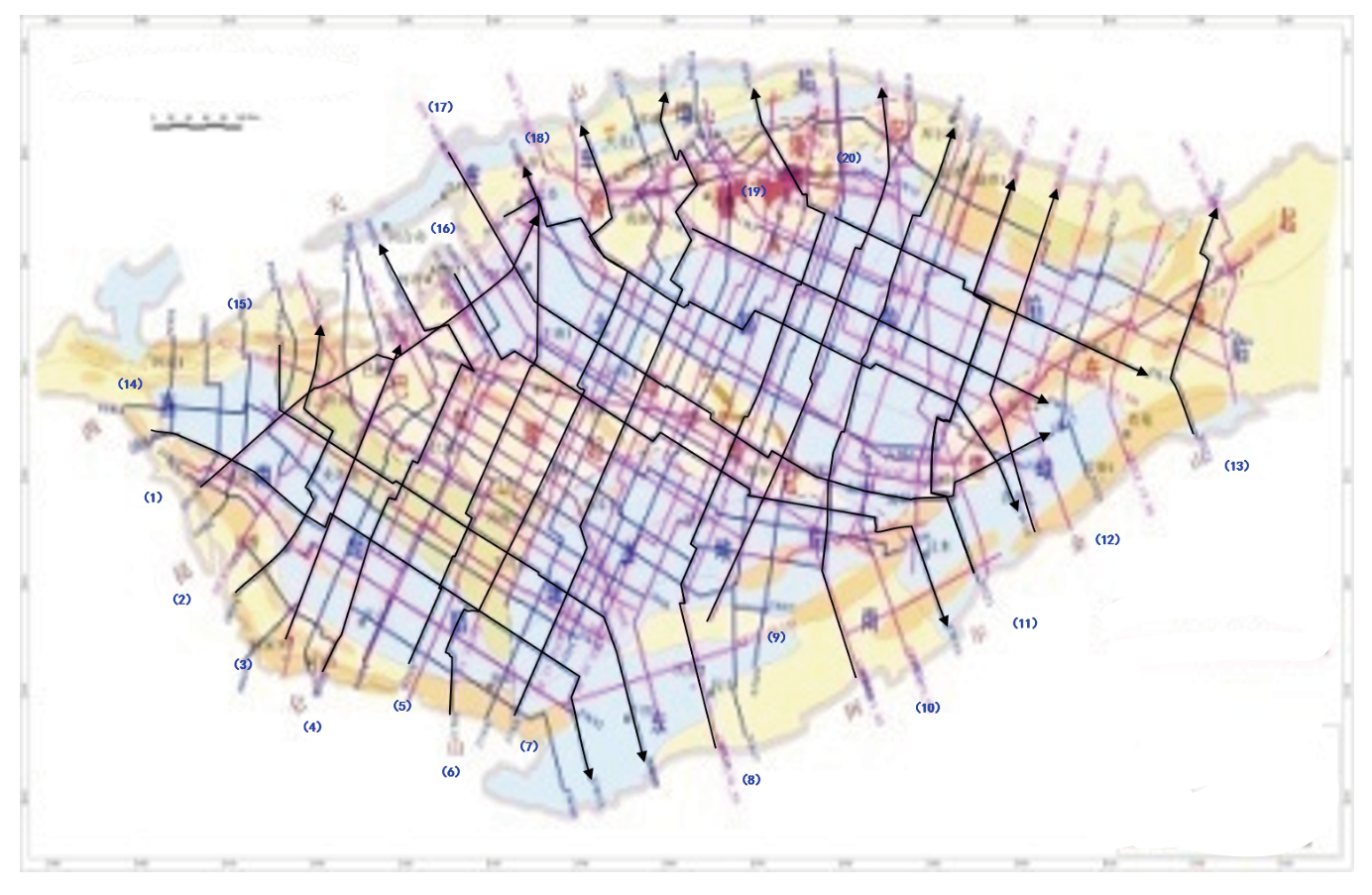

Supplement: S3 File — (ZIP) [file pone.0286849.s003.zip › 20 balanced geological transects from Tarim Oilfield Company/Distribution of the 20 survey lines.png]

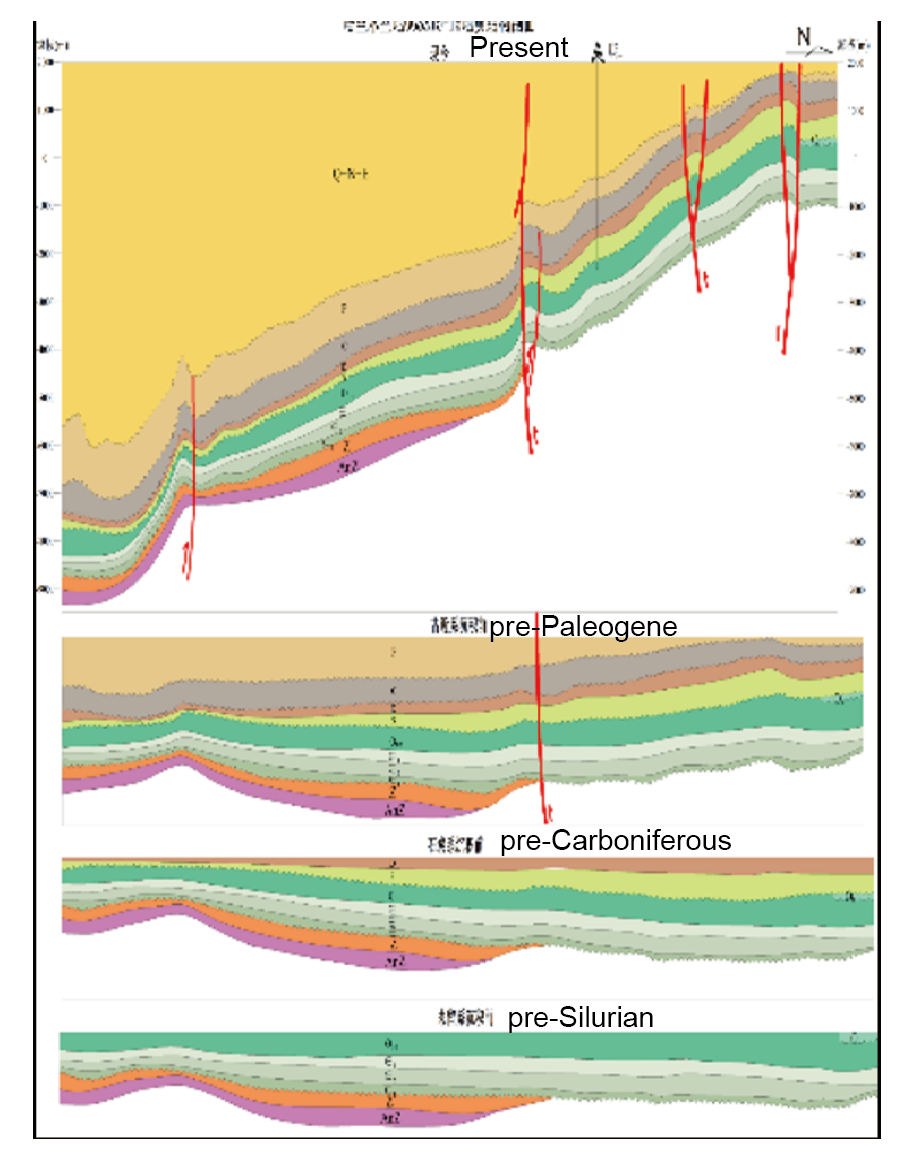

Supplement: S3 File — (ZIP) [file pone.0286849.s003.zip › 20 balanced geological transects from Tarim Oilfield Company/3.png]

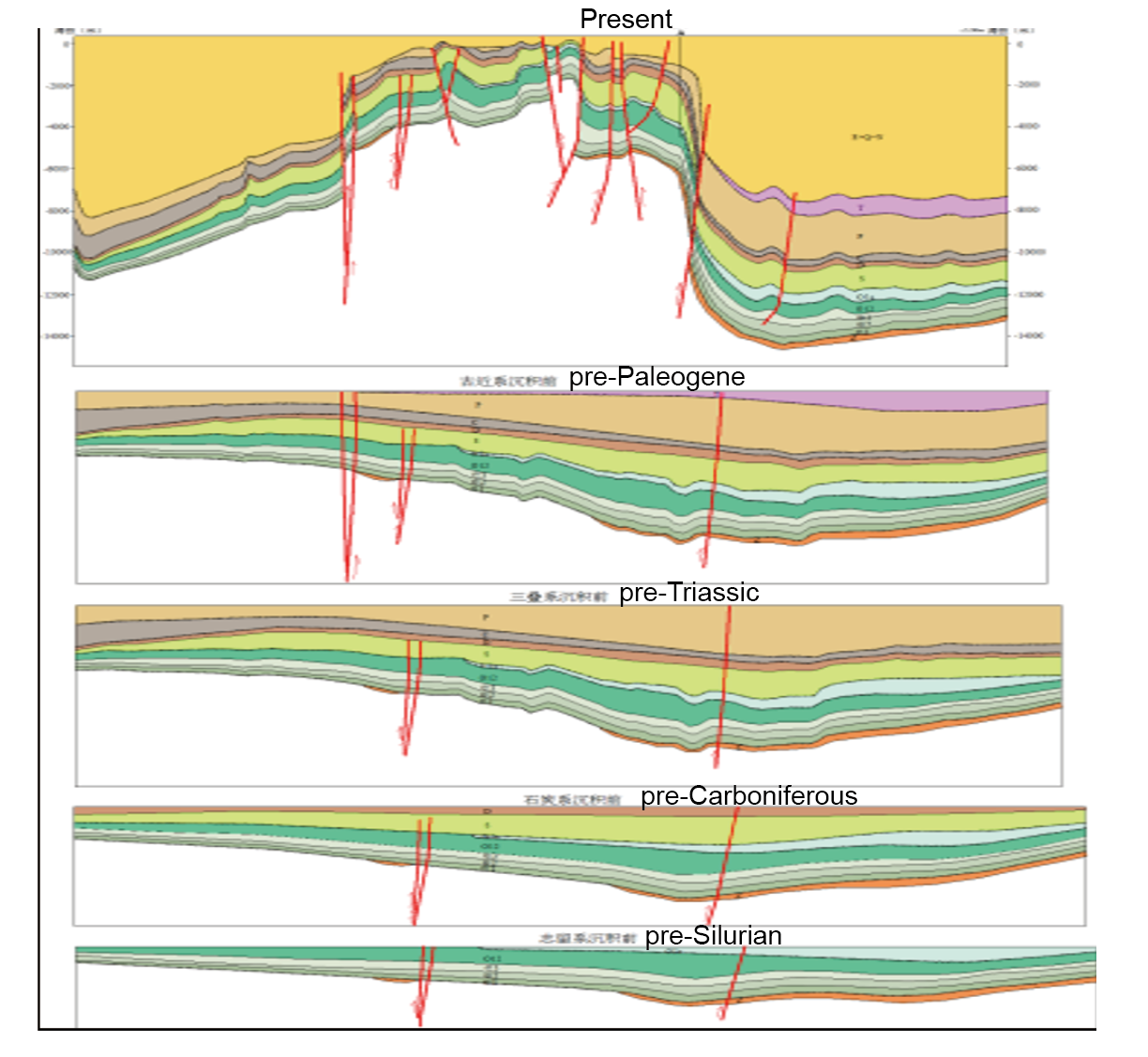

Supplement: S3 File — (ZIP) [file pone.0286849.s003.zip › 20 balanced geological transects from Tarim Oilfield Company/1.png]

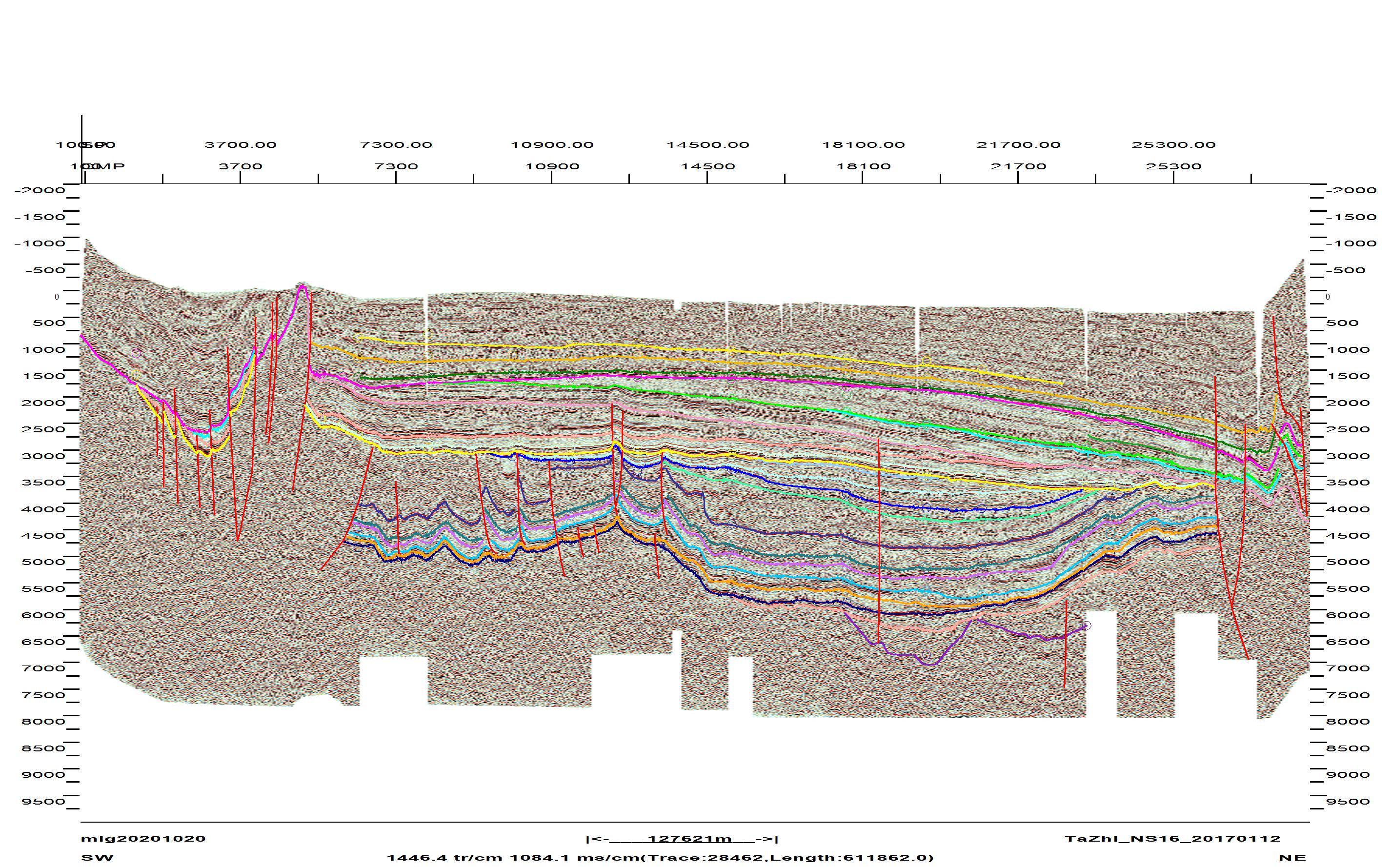

Supplement: S4 File — (ZIP) [file pone.0286849.s004.zip › 10 seismic profiles this study chose to restore the balanced geological transects/NS16.jpg]

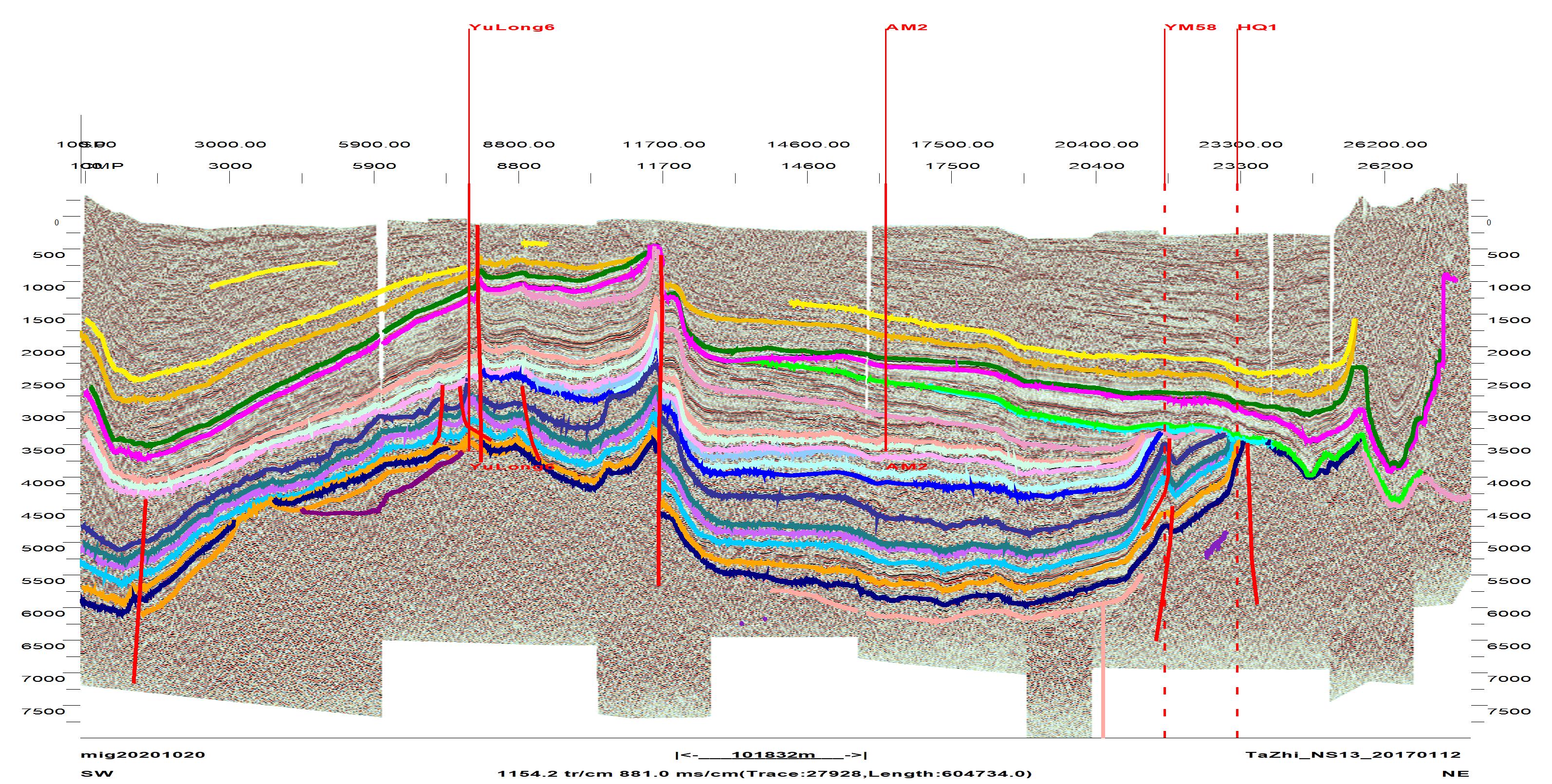

Supplement: S4 File — (ZIP) [file pone.0286849.s004.zip › 10 seismic profiles this study chose to restore the balanced geological transects/NS13.jpg]

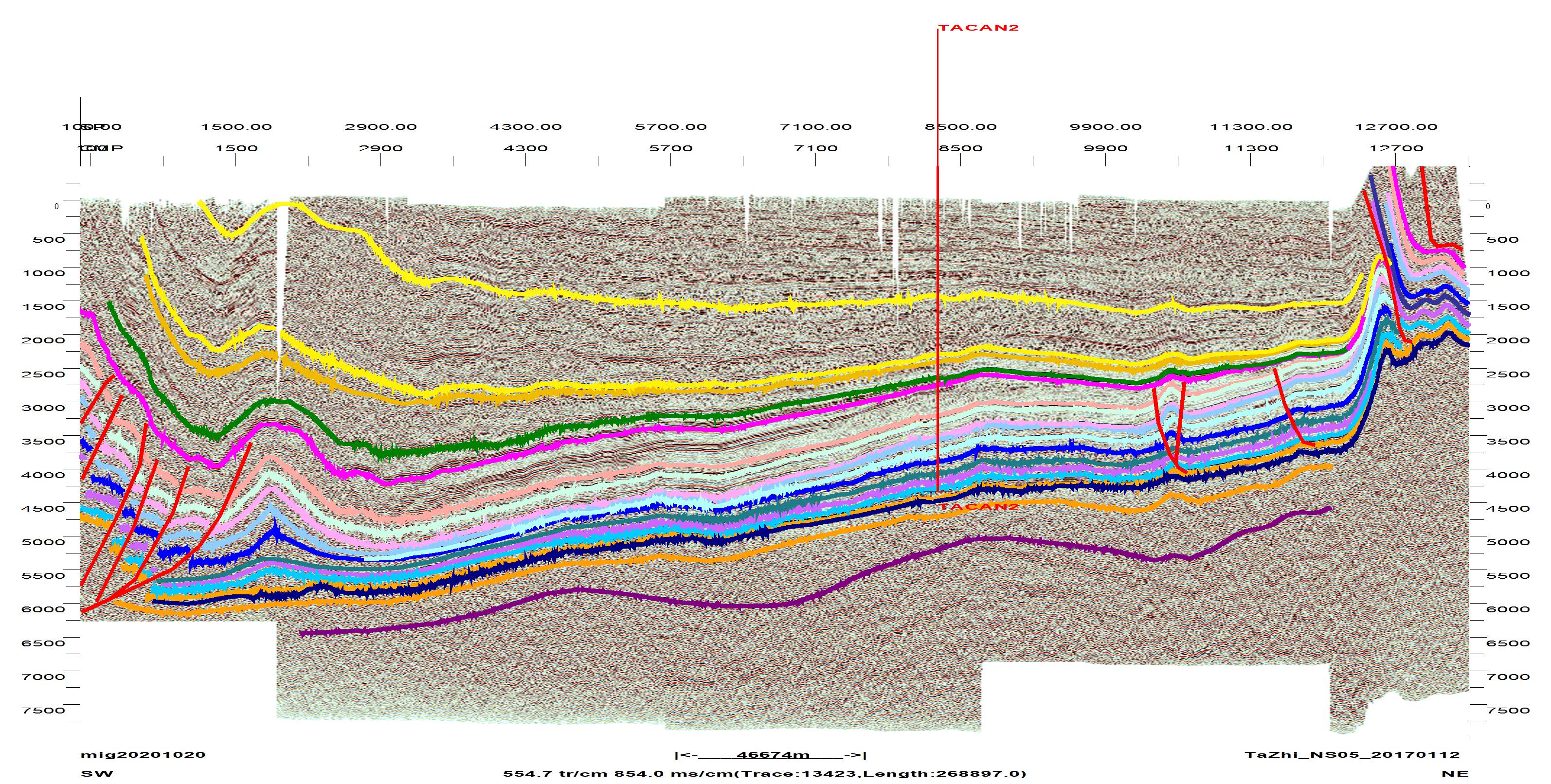

Supplement: S4 File — (ZIP) [file pone.0286849.s004.zip › 10 seismic profiles this study chose to restore the balanced geological transects/NS05.jpg]

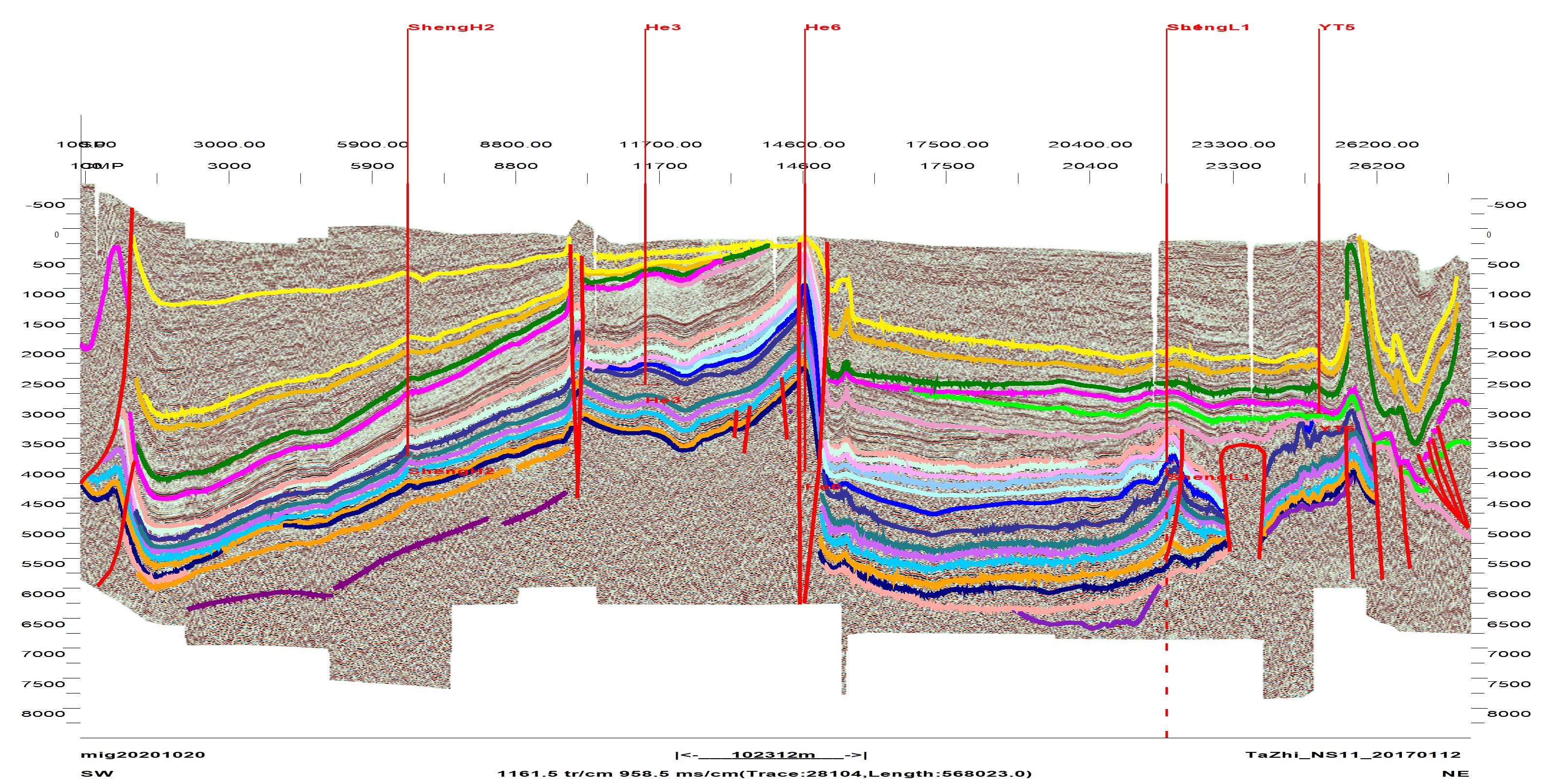

Supplement: S4 File — (ZIP) [file pone.0286849.s004.zip › 10 seismic profiles this study chose to restore the balanced geological transects/NS11.jpg]

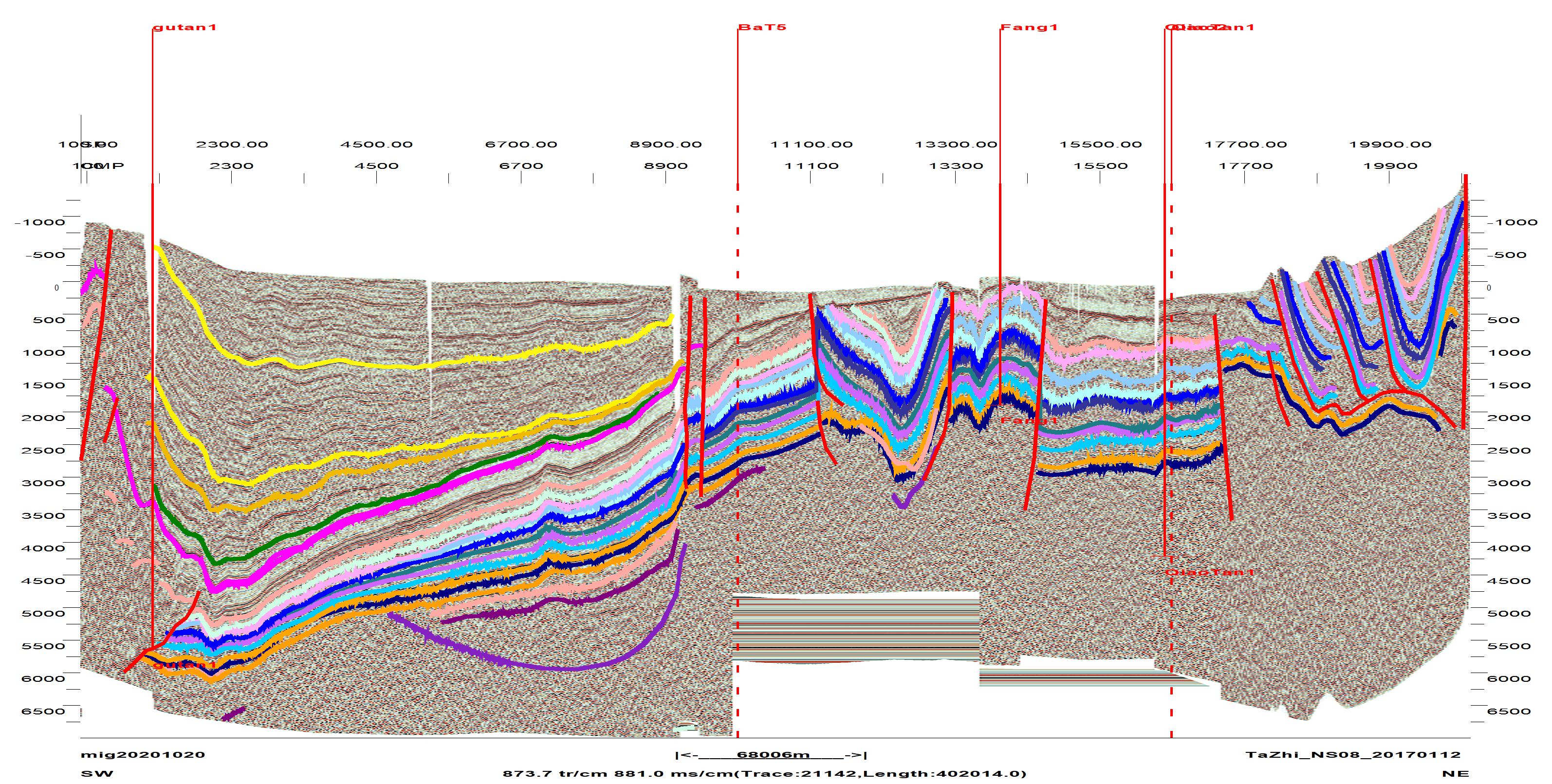

Supplement: S4 File — (ZIP) [file pone.0286849.s004.zip › 10 seismic profiles this study chose to restore the balanced geological transects/NS08.jpg]

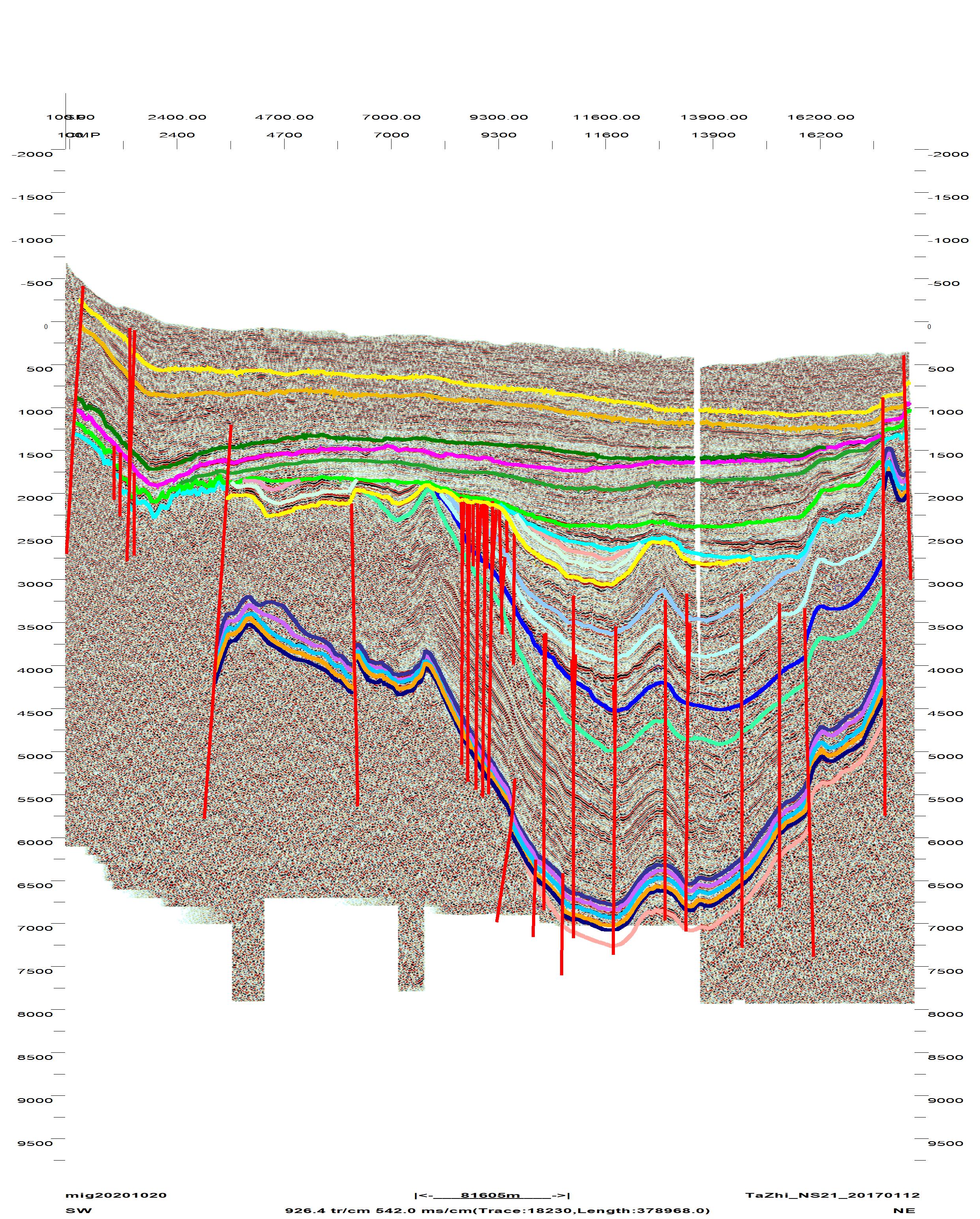

Supplement: S4 File — (ZIP) [file pone.0286849.s004.zip › 10 seismic profiles this study chose to restore the balanced geological transects/NS21.jpg]

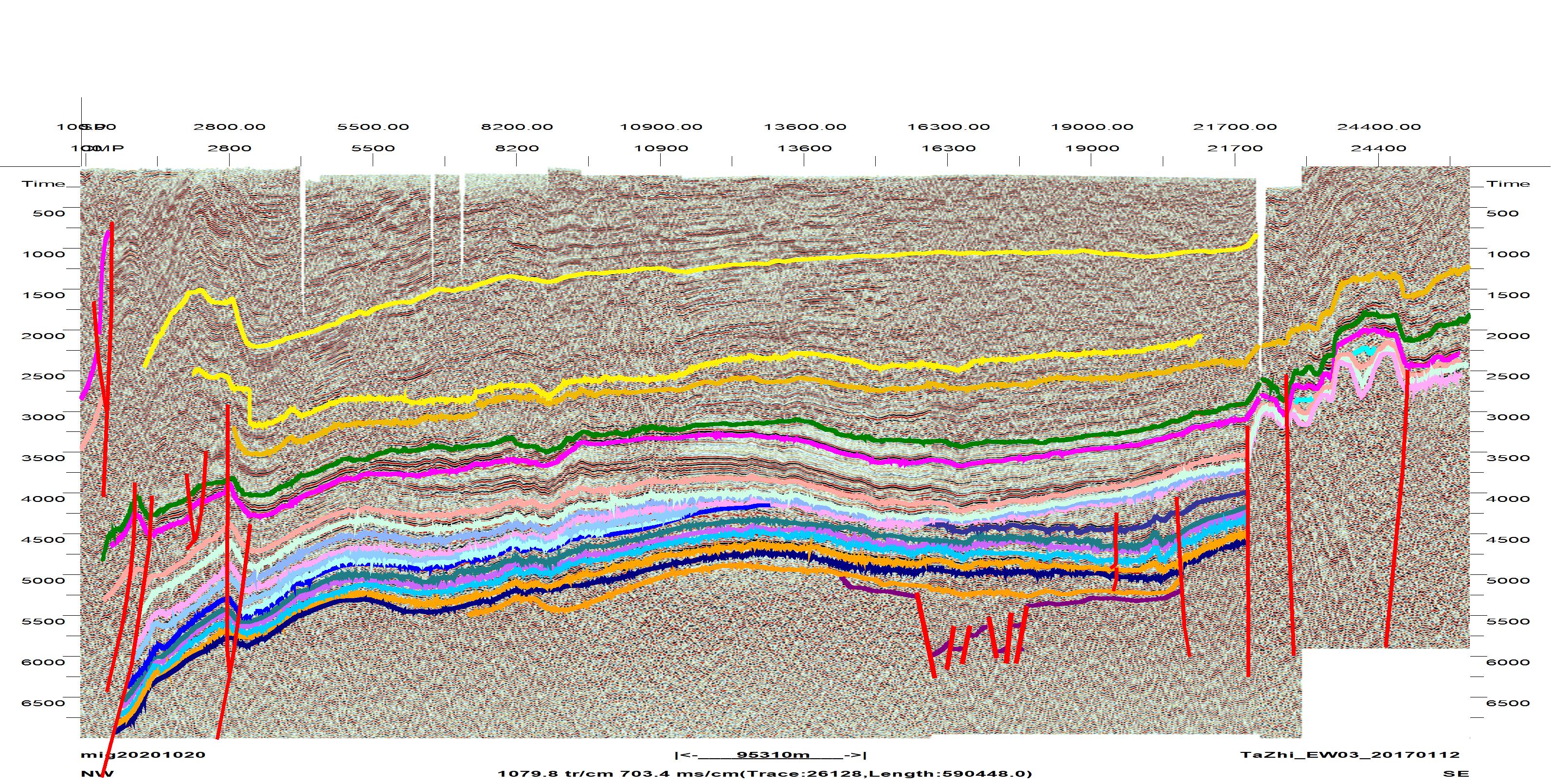

Supplement: S4 File — (ZIP) [file pone.0286849.s004.zip › 10 seismic profiles this study chose to restore the balanced geological transects/EW03.jpg]

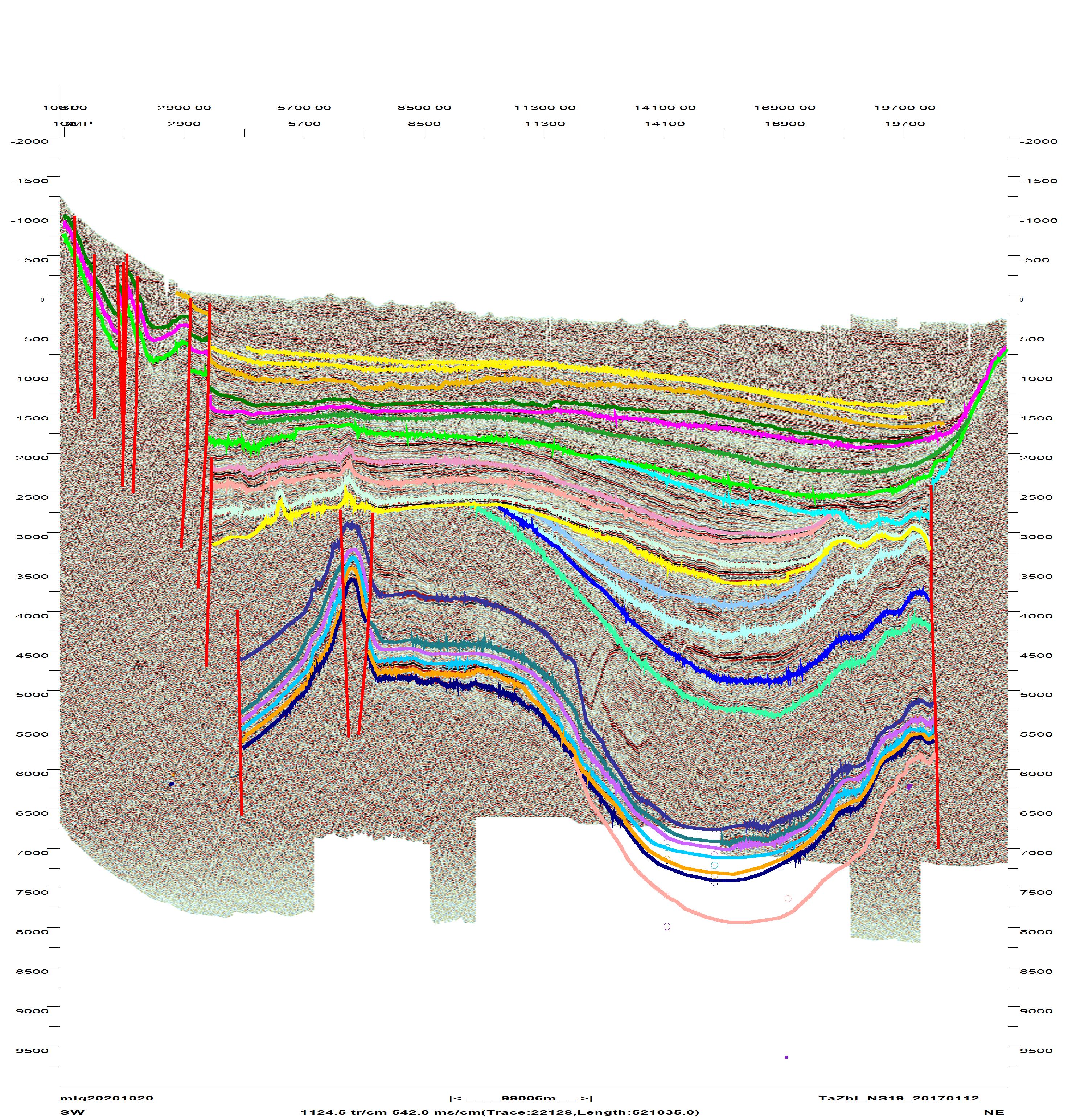

Supplement: S4 File — (ZIP) [file pone.0286849.s004.zip › 10 seismic profiles this study chose to restore the balanced geological transects/NS19.jpg]

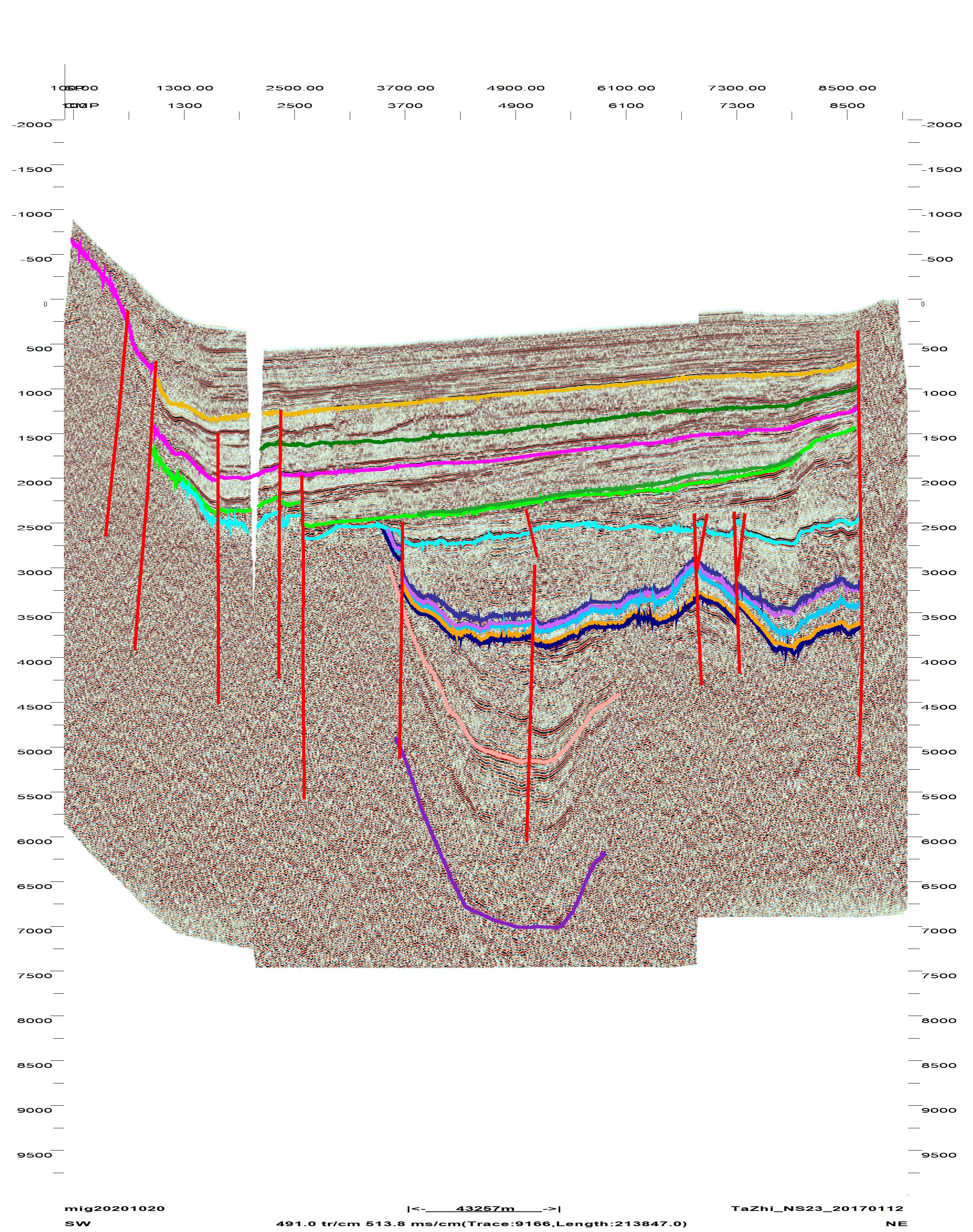

Supplement: S4 File — (ZIP) [file pone.0286849.s004.zip › 10 seismic profiles this study chose to restore the balanced geological transects/NS16+EW17+NS23/NS23.jpg]

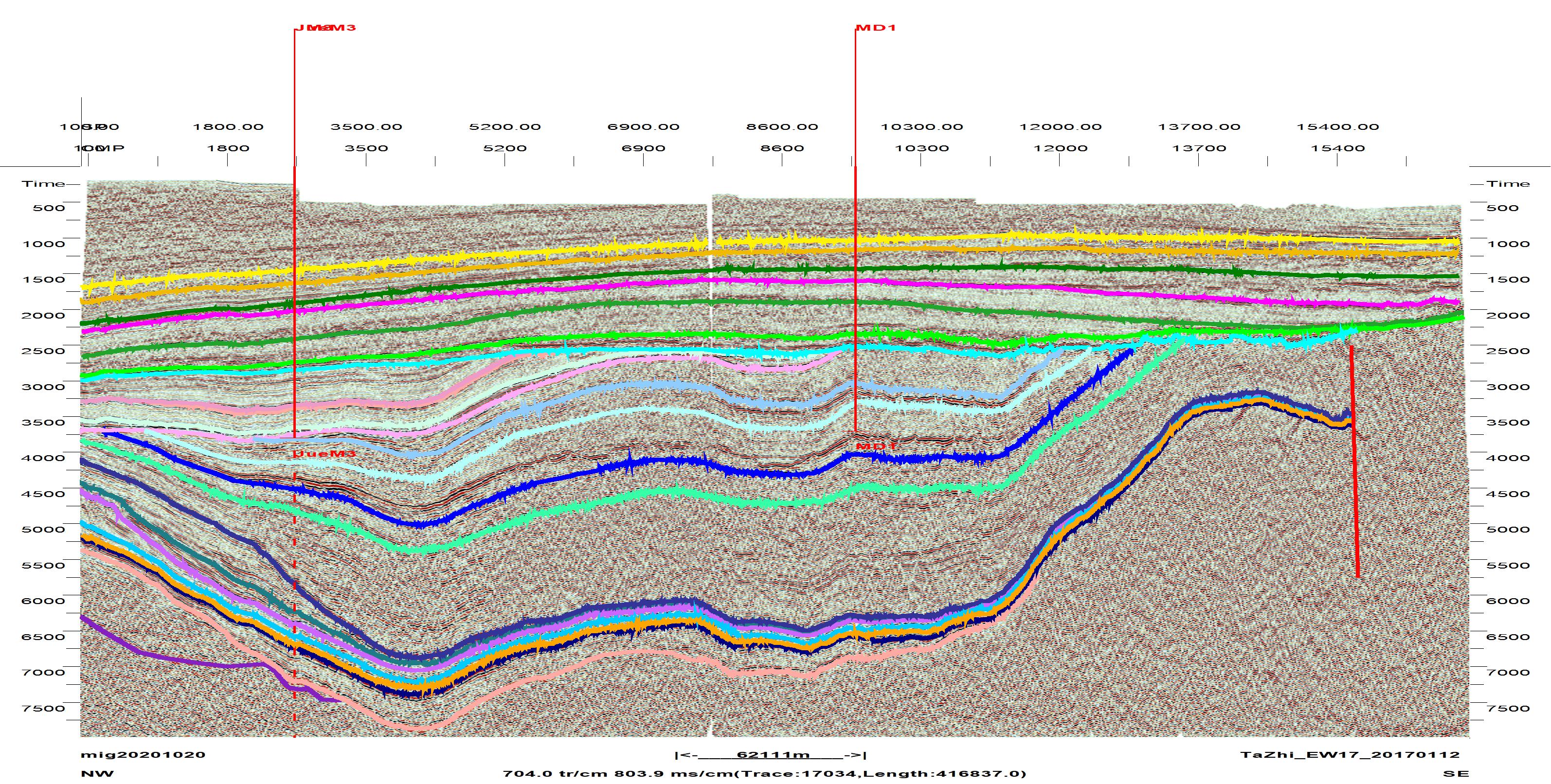

Supplement: S4 File — (ZIP) [file pone.0286849.s004.zip › 10 seismic profiles this study chose to restore the balanced geological transects/NS16+EW17+NS23/EW17.jpg]

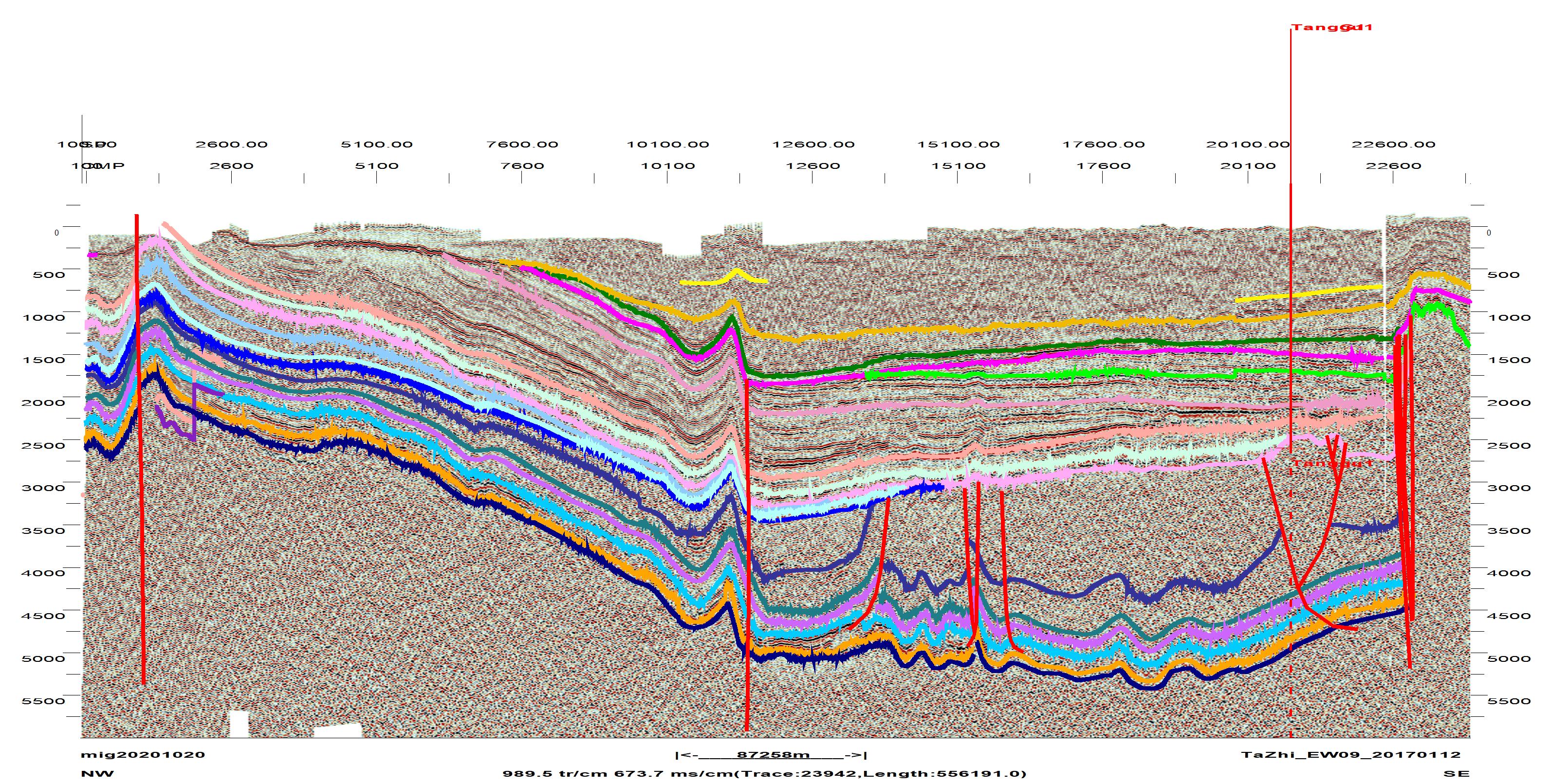

Supplement: S4 File — (ZIP) [file pone.0286849.s004.zip › 10 seismic profiles this study chose to restore the balanced geological transects/NS08+EW09+NS20/EW09.jpg]

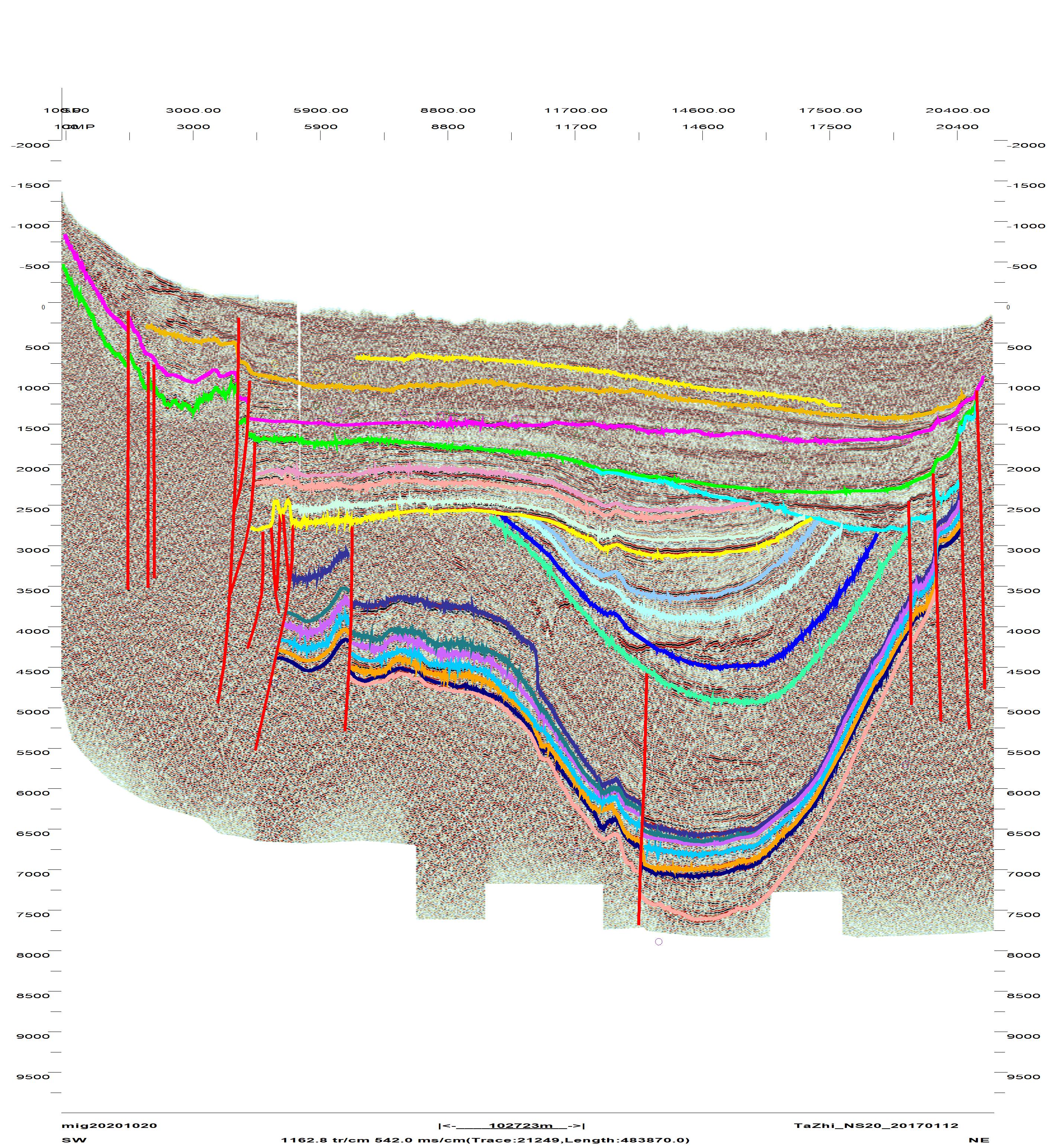

Supplement: S4 File — (ZIP) [file pone.0286849.s004.zip › 10 seismic profiles this study chose to restore the balanced geological transects/NS08+EW09+NS20/NS20.jpg]
